# Discovery of Novel Potential Aphid Repellents: Geranic Acid Esters Containing Substituted Aromatic Rings

**DOI:** 10.3390/molecules27185949

**Published:** 2022-09-13

**Authors:** Shixiang Pan, Wenhao Li, Yaoguo Qin, Zhaokai Yang, Yan Liu, Zhuo Shi, Cheng Qu, Chen Luo, Xinling Yang

**Affiliations:** 1 Innovation Center of Pesticide Research, Department of Applied Chemistry, College of Science, China Agricultural University, Beijing 100193, China; 2 MOA Key Laboratory for Monitoring and Environment-Friendly Control of Crop Pests, Department of Entomology, College of Plant Protection, China Agricultural University, Beijing 100193, China; 3 Institute of Plant Protection, Beijing Academy of Agriculture and Forestry Sciences, Beijing 100097, China

**Keywords:** molecular design, ester, aphids, repel, odorant-binding proteins, molecular docking

## Abstract

Aphids are one of the most damaging agricultural pests. For the sake of novel eco-friendly compounds with good activity for aphid control, a series of novel geranic acid esters containing substituted aromatic rings were designed by inverting ester groups of lead compounds. All compounds were characterized by HRMS, ^1^H-NMR, and ^13^C-NMR. In order to identify the effect of inversion ester groups on activity, a bioassay was conducted. The results showed that the repellent activity against *Acyrthosiphon pisum* (*A. pisum*) and the binding affinity with the odorant-binding protein 9 from *A. pisum* (ApisOBP9) of the compounds were increased after inversion of the ester groups. Particularly, **5f** showed the best repellent activity (repellency proportion: 55.6%) and binding affinity (1/Ki: 0.49 µM). Meanwhile, the structure–activity relationships revealed that the introduction of *meta-*substitution of the benzene ring and halogen atoms, such as Cl and Br, facilitated the biological activity. The further molecular docking results demonstrated that hydrogen bonding interactions and hydrophobic interactions were vital for the binding affinity with ApisOBP9. Additionally, all compounds were predicted to be eco-friendly and their volatile physicochemical properties have been enhanced compared to the leads. The present results provide valuable clues for the further rational design of aphids’ behavioral control agents.

## 1. Introduction

Aphids are one of the most destructive pests in agriculture and horticulture due to their multiplicity of species, rapid reproduction, wide host range, and tendency to develop resistance to insecticides [1,2,3]. The main method currently used for aphids’ control is chemical insecticides, which are fast-acting and provide excellent control of aphid. However, with the long-term unreasonable use of chemical pesticides, some problems have emerged, such as environmental pollution and toxicity to non-target organisms, especially to honeybees [4,5,6]. As a result, many neonicotinoid insecticides that are highly toxic to bees, such as imidacloprid, have limited use in Europe [7,8]. Therefore, the development of novel eco-friendly aphid control agents using new strategies is urgently needed.

**Scheme 1:**
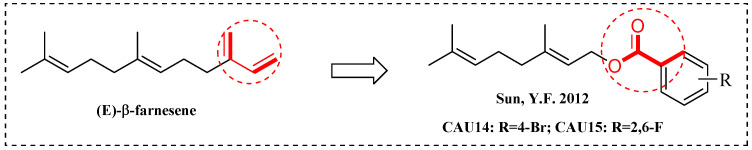
The structure of (E)-β-farnesene and lead compounds [22].

Insect pheromones play an important role in insect feeding, courtship, and aggregation and alarm, and have the advantages of being species-specific and harmless to the environment, which make them a promising eco-friendly agricultural pest management tool [9,10]. For aphids, the use of aphid pheromones might be an alternative way to control their populations by regulating their behavior, and is also considered to be beneficial for ecological conservation [11,12]. The aphid alarm pheromone is a very effective pheromone for controlling aphid populations, and is a mixture of (E)-β-farnesene (EβF), α-pinene, β-pinene, and β-limonene [13,14]; among them, EβF is the main component of the aphid alarm pheromone [15]. EβF regulates the behavior of aphids mainly through odor-binding proteins (OBPs) [16]. Insects have a sensitive olfactory system, where OBPs contribute a lot to the odor recognition process of insects [17,18]. The OBP genes of the *A. pisum* had been identified [19], and OBP3 and OBP7 have been shown to bind to EβF through competitive fluorescence binding assays [20,21,22]. Additionally, in our previous study, EβF was found to have the strongest binding affinity to ApisOBP9 compared to ApisOBP3 and ApisOBP7, implying that ApisOBP9 is the most critical potential target for action [23]. However, the conjugated double bonds in EβF’s structure (Scheme 1) result in its instability and thus hinder its application in the field. Therefore, it is important to develop analogs with both good activity and stability.

**Scheme 2:**
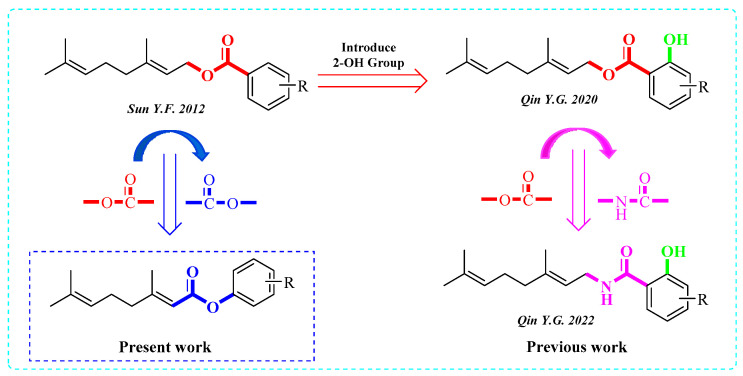
Design strategy of the target compounds [22,23,25].

Owing to compounds containing ester groups that play an important role in the chemical communication of insects [24], we designed and synthesized **CAU14** and **CAU15** by introducing ester groups using substituted benzene rings instead of its conjugated double bonds (Scheme 1). However, the result of the bioassay showed that they were not effective in repelling aphids [22]. Subsequently, we optimized the structure of those compounds by introducing the *ortho-*hydroxyl group and the compounds were evaluated to have an obvious promotion on repellent activities against aphids, but the repellent activities were greatly reduced by replacing the ester groups with the amide groups (Scheme 2) [23,25]. The above results indicated that the ester group was very important for maintaining the repellent activity of aphids.

Scaffold hopping [26,27] and bioisosterism [28] are widely used in the optimization of lead compounds in the pesticide discovery process. In this work, to identify if the activity can be influenced by inverting ester groups, a series of novel geranic acid esters containing substituted aromatic rings were firstly designed by reversing the direction of ester groups in lead compounds of **CAU14** and **CAU15** (Scheme 2). All the compounds were characterized by HRMS, ^1^H-NMR, and ^13^C-NMR. The repellent activities of these compounds were rated against *A. pisum*, and the binding affinities with ApisOBP9 were measured. Furthermore, molecular docking was performed to illustrate their possible binding mechanisms.

## 2. Results and Discussion

### 2.1. Synthesis of Target Compounds **5a–5x**

**Scheme 3:**
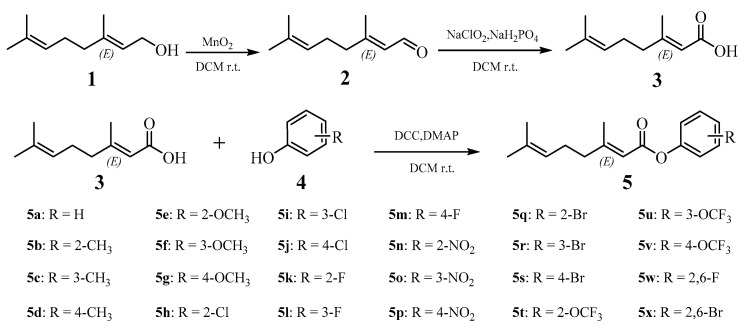
Synthetic route of target compounds **5a–5x**.

The synthetic route for the intermediates and target compounds was exhibited in Scheme 3. Initially, with regard to the synthesis of the key intermediate **2** (geranial, (*E*)-3,7-dimethylocta-2,6-dienal), the Dess-Martin Periodinane (DMP) was used as the oxidizing agent. At first, DMP and geraniol were dissolved separately by dichloromethane (DCM), and the diluted geraniol was slowly added dropwise into DMP under the condition of an ice-salt bath, and then the reactions were quenched with sodium bicarbonate (NaHCO_3_) and sodium thiosulfate after stirring 8 h at room temperature. Then, the filtrate was washed with sodium chloride and NaHCO_3_ after filtration. Finally, geranial was obtained in a medium yield by column chromatography. We found that using DMP as an oxidizer was a complex reaction process with high costs and a low yield. Therefore, we explored an alternative approach using manganese dioxide (MnO_2_) as an oxidizer, which can oxidize allyl alcohol. Firstly, MnO_2_ was added directly into starting material **1** (geraniol, (*E*)-3,7-dimethylocta-2,6-dien-1-ol)—which was dissolved by DCM—stirred at room temperature for 10 h and then filtered. Lastly, geranial was obtained in a high yield by directly spinning off the solvent under reduced pressure from the filtrate. We are pleased to find that using MnO_2_ as an oxidizer is not only a simple reaction with high yield compared to DMP, but also with a much lower cost, which is vital in large-scale production. 

According to the reported method [29], under moderate conditions, key intermediate **3** (geranic acid, (*E*)-3,7-dimethylocta-2,6-dienoic acid) was prepared in a high yield by adding geranial to the reaction system containing sodium chlorite (NaClO_2_), sodium dihydrogen phosphate (NaH_2_PO_4_), and 2-methyl-2-butene via the Pinnick Reaction in a one-pot method. After that, the target compounds were acquired in a 49–76% yield by the esterification of geranic acid with different commercially-available, substituted phenols under the conditions of using dicyclohexylcarbodiimide (DCC) as a condensation agent and 4-dimethylaminopyridine (DMAP) as a catalyst. The synthetic procedure is described in Section 3.2. The structures of all synthesized compounds **5a**–**5x** were confirmed by nuclear magnetic resonance hydrogen spectrum (^1^H-NMR), nuclear magnetic resonance carbon spectrum (^13^C-NMR), and electrospray ionization high-resolution mass spectrometry (ESI-HRMS). Their physical and chemical properties and structure characterization are presented in Section 3.2.3.

### 2.2. Repellent Activity

In our previous work [22], we found that lead compounds had poor repellent activity against aphids, however, the repellent activities of the compounds obtained by introducing an *ortho-*hydroxyl group to the benzene ring of the lead compounds were apparently enhanced [23], indicating that the structure of the lead compounds was a good class of molecular skeletons with repellent activity on aphids that deserved further optimization. Then, in order to investigate the effect of the orientation of the ester group on its activity, the repellent activity of the lead compounds prepared in our previous work [22] and newly-synthesized **5a**–**5x** had been tested against *A. pisum*, an important agricultural aphid. As shown in Table 1, the repellency proportion (RP) of newly-synthesized derivatives varied from 23.4% to 55.6%. 

**Figure 1:**
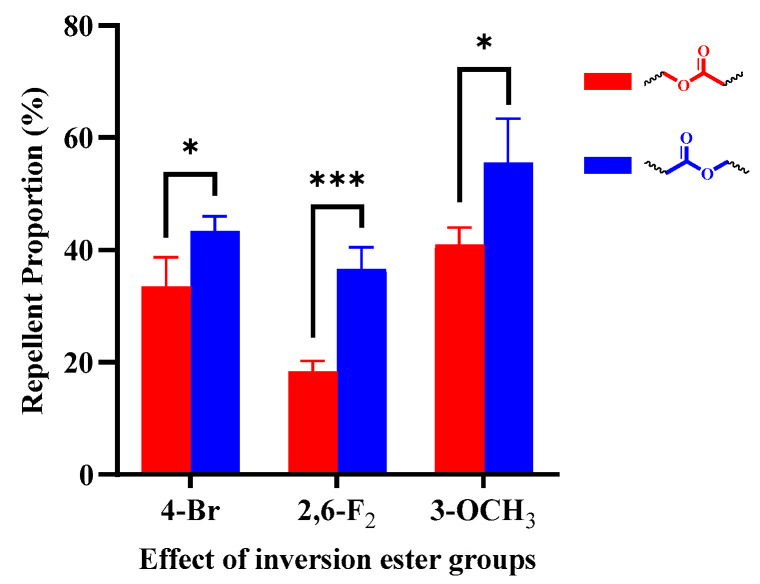
The effect of inversion ester groups on repellent activity against *A. pisum*; Data represent the mean ± standard deviation. Significance calculated using T test (* *p* < 0.05, *** *p* < 0.001).

As shown in Figure 1, the repellent activities of compounds **5s** (43.4%) and **5w** (36.7%) were higher than that of lead **CAU14** (33.5%) and **CAU15** (18.3%), respectively. The difference in structure between them was only the direction of the ester group. At the same time, **CAU13**, a lead compound paired with **5f,** was prepared according to the literature in the Supporting Information [21], and its repellent activity against *A. pisum* was evaluated for the first time in this work. It can be clearly seen from Figure 1 that the repellent activity of **CAU13** (41.0%) was significantly lower than that of **5f** (55.6%). These results indicated that the repellent activity against aphids could be enhanced by the inversion of the ester groups. 

**Figure 2:**
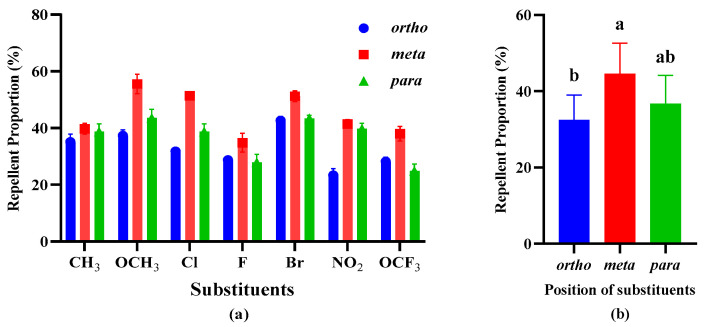
(**a**) The order of the repellency proportion of monosubstituents against *A. pisum*; (**b**) The positional effects of *ortho-*, *meta-*, and *para-*substituents on repellent activity. Data represent the mean ± standard deviation. A one-way analysis of variance (ANOVA) was performed followed by Duncan’s test (Different letters indicate values are significantly different (*p* < 0.05)).

As depicted in Figure 2a,b, the position of the substituents on the benzene ring of both the electron-absorbing substituents and the electron-donating substituents had an obvious effect on the repellent activity, with the *meta-*substituted compounds showing significantly better repellent activity than the *ortho-*substituted ones, but the repellent activity of both the *ortho-*substituted and *meta-*substituted ones were statistically insignificantly different from that of the *para-*substituted one. When halogen atoms are introduced into the benzene ring, the introduction of the F atom seemed to be detrimental to the repellent activity (**5i** ≈ **5r** > **5l; 5f** > **5u**). Furthermore, for the same substituent, disubstituents did not contribute much to the enhancement of repellent activity (**5x** ≈ **5q**–**5s**; **5w** ≈ **5k**–**5m**) in Table 1.

### 2.3. The Binding Affinity to ApisOBP9

Insects were repelled mainly due to their olfactory system sensing odor molecules, given that OBPs play an important role in this process. The ApisOBP9 was identified as a key potential target for the repellent activity to aphids in our previous work [23]. Therefore, in order to figure out the reason for the change in repellent activity after the ester group inversion, the binding affinities of the lead compounds and target compounds to ApisOBP9 were determined by a competitive fluorescence binding experiment using N-Phenyl-1-naphthylamine (1-NPN) as a probe. Their binding curves and binding constant (1/Ki) values were shown in Figure S1 and Table 2, respectively.

**Figure 3:**
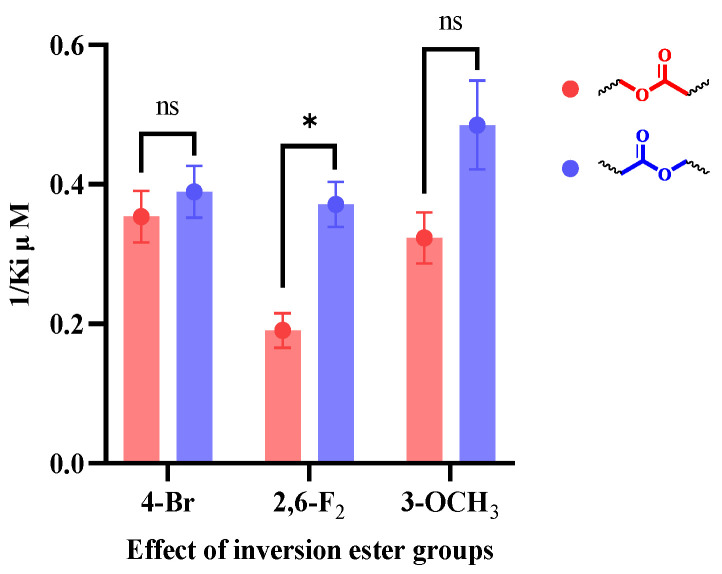
The effect of inversion ester groups on binding affinity with ApisOBP9; Data represent the mean ± standard deviation. Significance calculated using *T* test (* *p* < 0.05; ns *p* > 0.05).

**Figure 4:**
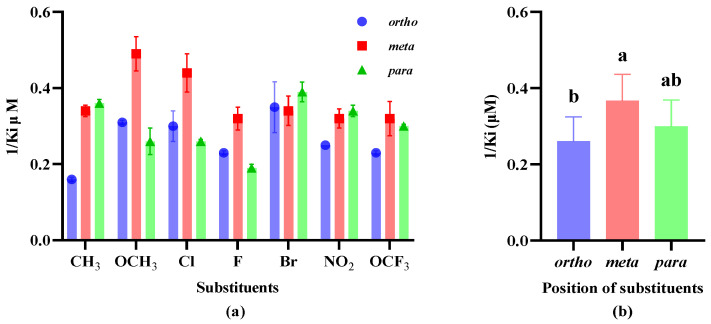
(**a**) The order of the binding affinity of monosubstituents with ApisOBP9; (**b**) The positional effects of *ortho*-, *meta-*, and *para-*substituents on binding affinity. Data represent the mean ± standard deviation. A one-way analysis of variance (ANOVA) was performed followed by Duncan’s test (Different letters indicate values are significantly different (*p* < 0.05)).

The results displayed that all compounds were able to bind ApisOBP9 with 1/Ki values ranging from 0.16–0.49 μM. Similar to the repellent activity, the protein-binding affinity of **5f** (0.49 μM), **5s** (0.39 μM), and **5w** (0.37 μM) were better than that of **CAU13** (0.32 μM), **CAU14** (0.35 μM), and **CAU15** (0.19 μM) after the ester groups’ inversion in Figure 3, respectively. It is suggested that the inversion of the ester groups may be more favorable for binding with ApisOBP9, leading to an increase in repellent activity. As shown in Figure 4a,b, the position of the substituents on the benzene ring also played vital roles in binding affinity, where the binding ability of the *meta-*substituted compounds was also significantly better than that of the *ortho-*substituted compounds. Furthermore, the binding affinity of both the *ortho-*substituted and *meta-*substituted ones were not significantly different from that of the *para*-substituted ones in statistics, which was consistent with the repellent activity. Similarly, when halogen atoms were introduced, the introduction of an F atom was detrimental to the protein-binding affinity (**5i** > **5l**; **5f** > **5u**). In our previous study [23], we also found that the repellent activities and the binding affinities with ApisOBP9 of *meta-*substituted compounds were significantly better than those of compounds substituted at other positions, implying that the *meta-*position was critical for biological activity. 

### 2.4. Molecular Docking of Ligands to ApisOBP9

To further study the molecular mechanisms of compounds binding with ApisOBP9, compounds **CAU15** and **5w** were selected as representative paired compounds to study the effect of ester group reversing, while **5e**–**5g** were selected as representative compounds to explore the position impact of substituents on benzene rings. The structure of the ApisOBP9 protein has been modeled in our previous work using a more accurate method (named trRosetta) for protein structure prediction [30,31].

**Figure 5:**
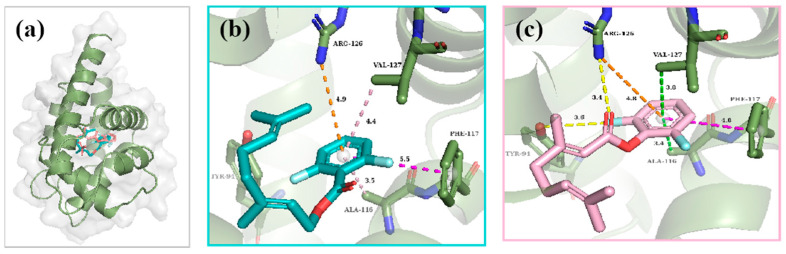
(**a**) Superimposed conformations of the molecular docking ligands; (**b**) The docking conformation of **CAU15**; (**c**) The docking conformation of **5w**.

**CAU15** and **5w** were both located in the central area of the ApisOBP9 binding pocket, and the different conformations are presented in Figure 5a. The hydrophobic long chain of **CAU15** folded toward the center of the pocket and the carbonyl in the ester group extended to the outside of the pocket. **CAU15** formed pi-cation interactions with Arg 126 and pi-alkyl interactions with Ala116 and Val127, which are shown in Figure 5b. As shown in Figure 5c, the hydrophobic long chain of **5w** stretched outward from the center of the binding pocket, while the carbonyl of the ester group stretched toward the center of the pocket to form hydrogen bond interactions with Arg126 and Tyr94, respectively. The molecular docking results indicated that the inversion of the ester groups resulted in a large change in molecular conformation, which could increase the hydrogen bonding interactions with Arg126 and Tyr94, and thus enhance the binding affinity to ApisOBP9.

**Figure 6:**
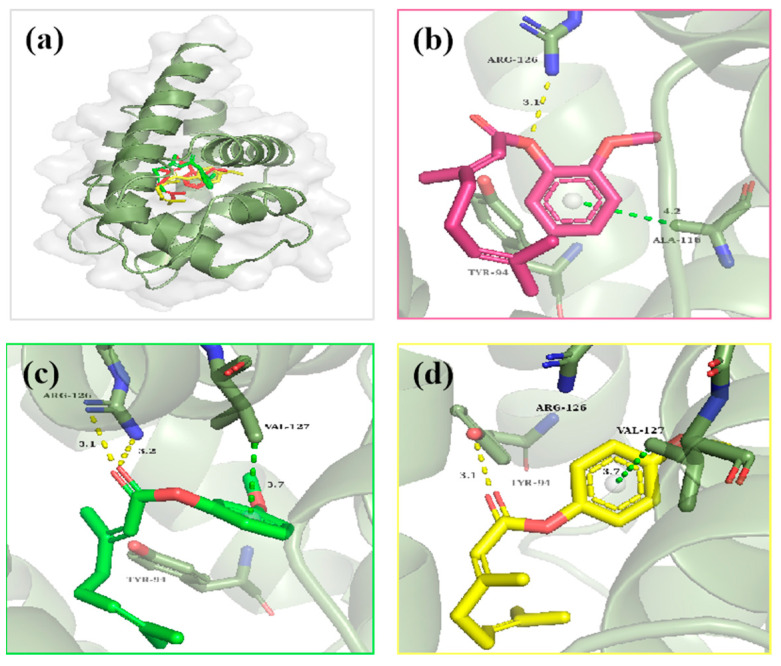
(**a**) Superimposed conformations of the molecular docking ligands; (**b**) The docking conformation of **5e** (*ortho-*); (**c**) The docking conformation of **5f** (*meta-*); (**d**) The docking conformation of **5g** (*para-*).

As illustrated in Figure 6, similar to compound **5w**, the carbonyl groups of compounds **5e**, **5f**, and **5g** all reached toward the center of the binding pocket to form hydrogen bond interactions with Arg126 or Tyr94, where compound **5f** could form two hydrogen bond interactions with Arg126, and thus exhibited a better affinity with ApisOBP9 than the other two. Although both of their hydrophobic geranyl chains extended to the outside of the binding pocket, the long hydrophobic chain of **5e** was folded, while those of **5f** and **5g** were more extended, hence **5f** and **5g** were able to form stronger hydrophobic interactions with ApisOBP9 than **5e**. In summary, we speculated that hydrogen bonding interactions formed by compounds with key amino acids, such as Arg126 or Tyr94, and the stretching of the long hydrophobic chains are important for their binding ability towards ApisOBP9.

### 2.5. Vapor Pressure and Normal Boiling Point Prediction of Lead and Target Compounds

Using the bioisosterism strategy to design molecules not only provides compounds with novel skeletons but also with enhanced physicochemical properties [32]. The level of vapor pressure and boiling point affects the volatility of a molecule. The higher the vapor pressure of a molecule and the lower the boiling point, the more suitable it is to be used as a volatile chemical signal [24]. Therefore, the change in the vapor pressure and boiling point of compounds before and after the inversion of the ester groups were predicted by the CompTox Chemicals Dashboard [33].

The prediction of the vapor pressure results is shown in Table 3. It can be clearly seen that the vapor pressure of the target compounds obtained after inversion of the ester groups was increased. The above data indicated that the volatility of the compounds after ester group reversal was increased to different degrees, where the 2,6-F-substituted compound showed the greatest change, which was also consistent with the results of the repellent activity bioassay. As depicted in Table 3, the normal boiling point of the target compounds were decreased compared with the lead compounds. This means that the volatility of the target compounds was increased to varying degrees to facilitate their conduction as chemical signals. Thus, the inversion of ester groups not only affects the protein-binding affinity, but also changes the physicochemical properties to guide the repellent activity.

### 2.6. Honeybee Toxicity, Carcinogenicity, and Rat Oral Toxicity Prediction of Lead and Target Compounds

To evaluate the safety of all the compounds for beneficial insects and mammals, the honeybee toxicity, carcinogenicity. and rat oral toxicity predictions were performed by admetSAR, ProTox-II, and CompTox Chemicals Dashboard, respectively [33,34,35]. As presented in Table 4, all the synthesized compounds were inactive to carcinogenicity and had low toxicity against honeybees and rats. Accordingly, in the future, compounds **5a**–**5x** could be used as eco-friendly repellents for aphid control in field crops. 

## 3. Materials and Methods

### 3.1. General Information

The melting point of **5p** was determined on a Cole-Parmer apparatus equipped with an uncorrected thermometer (Shanghai precision instrument and Meter Co., Ltd., Shanghai, China). All utilized laboratory reagents (analytical grade) were acquired from Energy Chemical and used without additional purification. Silica gel (200–300 mesh, Puke Corporation, Qingdao, China) was used for column chromatographic purification with petroleum ether and ethyl acetate as eluents. The ^1^H NMR spectra and ^13^C NMR spectra of the compounds, **5a**–**5x,** were determined on an AVANCE NEO 500M spectrometer (Bruker, Bremen, Germany) using chloroform-*d* (CDCl_3_) as a solvent and tetramethylsilane (TMS) as an internal standard. High-resolution mass spectrometry (HRMS) data of compounds **5a**–**5x** were obtained on a 7.0T FTICR-MS instrument (Varian, Palo Alto, CA, USA). The fluorescence binding assay data were obtained with an RF-6000 Spectrofluorophotometer (SHIMADZU, Kyoto, Japan).

### 3.2. Synthesis of Target Compounds **5a–5x**

#### 3.2.1. Procedure for the Preparation of Intermediate **2** (Geranial)

The synthesis of geranial was started by stirring geraniol (Starting materials **1**, 30 mmol) with MnO_2_ (300 mmol) in DCM (80 mL) in an ice bath for 30 min. Then the ice bath was removed, and the reaction was completed by thin-layer chromatography (TLC), which was used to verify the reaction after stirring for 10 h. After that, in the state of suction filtration, we added 15 g of 200–300 mesh silica gel to Buchner funnels with fritted discs, which were slowly dropped into the mixture in the funnel and eluted with 100 mL of DCM and ethyl acetate (EA), respectively. The reaction mixture was evaporated under reduced pressure to remove the solvent to obtain geranial as a light-yellow liquid at a 98.6% yield. ^1^H NMR (500 MHz, Chloroform-d) δ 9.99 (d, J = 8.1 Hz, 1H), 5.88 (dt, J = 8.1, 1.3 Hz, 1H), 5.07 (tt, J = 6.6, 1.6 Hz, 1H), 2.30–2.18 (m, 4H), 2.17 (d, J = 1.3 Hz, 3H), 1.69 (d, J = 1.7 Hz, 3H), 1.61 (d, J = 1.4 Hz, 3H). ^13^C NMR (126 MHz, Chloroform-d) δ 191.3, 163.9, 132.9, 127.4, 122.6, 40.6, 25.7, 25.6, 17.7, 17.6.

#### 3.2.2. Procedure for the Preparation of Intermediate **3** (Geranic Acid)

According to the literature reporting method [29], we added NaClO_2_ (0.123 mol) and NaH_2_PO_4_ (0.143 mol) to 100 mL of water to obtain solution A, and then geranial (16.9 mmol) and 2-methyl-2-butene (27.5 mL) were added into acetone (100 mL). Then, solution A was slowly added into the mixture containing geranial for 30 min. The reaction was completed by TLC, which verified the reaction after stirring for 2 h, and was followed by extraction with EA, drying with anhydrous sodium sulfate, and being subsequently filtered. The organic phase was concentrated under a reduced pressure and purified by column chromatography to obtain geranic acid as a light-yellow liquid at a 90.1% yield. ^1^H NMR (500 MHz, Chloroform-d) δ 5.69 (s, 1H), 5.07 (q, J = 1.7 Hz, 1H), 2.53–1.99 (m, 7H), 1.69 (s, 3H), 1.61 (s, 2H). ^13^C NMR (126 MHz, Chloroform-d) δ 172.3, 163.1, 132.7, 122.8, 115.2, 41.2, 26.0, 25.7, 19.2, 17.7.

#### 3.2.3. General Procedure for the Preparation of Compounds **5a**–**5x**

The synthesis of compound **5a** (Scheme 3) was started by stirring intermediate **3** (5 mmol) with phenol (5 mmol) in DCM (30 mL) in an ice bath. Then dicyclohexylcarbodiimide (DCC, 6 mmol) and 4-dimethylaminopyridine (DMAP, 0.6 mmol) were added into the reaction and stirred for 30 min. After that, it was removed from the ice bath and the mixture was finished by TLC, which detected the compound after stirring for 8 h, and was subsequently washed with saturated aqueous sodium bicarbonate solution 3 times, extracted with DCM, dried with anhydrous sodium sulfate, and then filtered. The organic phase was concentrated under a reduced pressure and purified by column chromatography to obtain **5a** as a colorless liquid at a 42.5% yield. ^1^H NMR (500 MHz, Chloroform-d) δ 7.39–7.36 (m, 2H), 7.23–7.19 (m, 1H), 7.12–7.09 (m, 2H), 5.91 (q, J = 1.2 Hz, 1H), 5.13–5.09 (m, 1H), 2.25–2.21 (m, 7H), 1.71 (d, J = 1.4 Hz, 3H), 1.64 (d, J = 1.4 Hz, 3H). ^13^C NMR (126 MHz, Chloroform-d) δ 165.1, 163.3, 150.8, 132.8, 129.3, 125.5, 122.9, 121.8, 114.7, 41.2, 26.1, 25.7, 19.2, 17.7. HRMS (ESI) *m*/*z* calcd for C_16_H_21_O_2_ [M + H]^+^: 245.1536; found, 245.1539.

Compounds **5b**–**5x** were synthesized using a similar procedure as compound **5a**.

*2-tolyl (E)-3,7-dimethylocta-2,6-dienoate* (**5b**), colorless liquid, 49.5% yield. ^1^H NMR (500 MHz, Chloroform-d) δ 7.24–7.18 (m, 2H), 7.12 (td, J = 7.4, 1.4 Hz, 1H), 7.02 (dd, J = 7.8, 1.4 Hz, 1H), 5.94 (q, J = 1.2 Hz, 1H), 5.14–5.10 (m, 1H), 2.27–2.21 (m, 7H), 2.19 (s, 3H), 1.71 (d, J = 1.5 Hz, 3H), 1.64 (d, J = 1.4 Hz, 3H). ^13^C NMR (126 MHz, Chloroform-d) δ 164.9, 163.2, 149.4, 132.8, 131.0, 130.4, 126.8, 125.8, 122.9, 122.2, 114.5, 41.2, 26.1, 25.7, 19.2, 17.8, 16.3. HRMS (ESI) *m*/*z* calcd for C_17_H_23_O_2_ [M + H]^+^: 259.1693; found, 259.1697.

*3-tolyl (E)-3,7-dimethylocta-2,6-dienoate* (**5c**), light-yellow liquid, 60.2% yield. ^1^H NMR (500 MHz, Chloroform-d) δ 7.24 (d, J = 7.6 Hz, 1H), 7.02 (d, J = 7.6 Hz, 1H), 6.93–6.88 (m, 2H), 5.90 (s, 1H), 5.14–5.09 (m, 1H), 2.35 (s, 3H), 2.22 (d, J = 7.0 Hz, 7H), 1.71 (s, 3H), 1.63 (s, 3H). ^13^C NMR (126 MHz, Chloroform-d) δ 165.2, 163.1, 150.7, 139.5, 132.7, 129.1, 126.3, 122.9, 122.4, 118.8, 114.8, 41.2, 26.1, 25.7, 21.3, 19.2, 17.7. HRMS (ESI) *m*/*z* calcd for C_17_H_23_O_2_ [M + H]^+^: 259.1693; found, 259.1697.

*4-tolyl (E)-3,7-dimethylocta-2,6-dienoate* (**5d**), light-yellow liquid, 49.3% yield. ^1^H NMR (500 MHz, Chloroform-d) δ 7.17 (d, J = 8.2 Hz, 2H), 6.98 (d, J = 8.4 Hz, 2H), 5.89 (q, J = 1.3 Hz, 1H), 5.13–5.09 (m, 1H), 2.34 (s, 3H), 2.24–2.21 (m, 7H), 1.71 (d, J = 1.3 Hz, 3H), 1.63 (d, J = 1.3 Hz, 3H). ^13^C NMR (126 MHz, Chloroform-d) δ 165.3, 163.0, 148.5, 135.1, 132.7, 129.8, 122.9, 121.5, 114.8, 41.2, 26.1, 25.7, 20.9, 19.2, 17.7. HRMS (ESI) *m*/*z* calcd for C_17_H_23_O_2_ [M + H]^+^: 259.1693; found, 259.1694.

*2-methoxyphenyl (E)-3,7-dimethylocta-2,6-dienoate* (**5e**), colorless liquid, 58.5% yield. ^1^H NMR (500 MHz, Chloroform-d) δ 7.21–7.17 (m, 1H), 7.06 (dt, J = 7.9, 1.3 Hz, 1H), 6.99–6.93 (m, 2H), 5.99–5.94 (m, 1H), 5.14–5.10 (m, 1H), 3.83 (s, 3H), 2.25–2.20 (m, 7H), 1.71 (s, 3H), 1.63 (s, 3H). ^13^C NMR (126 MHz, Chloroform-d) δ 164.6, 163.0, 151.4, 139.7, 132.7, 126.6, 123.2, 123.0, 120.8, 114.4, 112.4, 55.9, 41.2, 26.1, 25.7, 19.2, 17.7. HRMS (ESI) *m*/*z* calcd for C_17_H_23_O_3_ [M + H]^+^: 275.1642; found, 275.1641.

*3-methoxyphenyl (E)-3,7-dimethylocta-2,6-dienoate* (**5f**), colorless liquid, 58.0% yield. ^1^H NMR (500 MHz, Chloroform-d) δ 7.28 (d, J = 8.2 Hz, 1H), 6.77 (dd, J = 8.4, 2.5 Hz, 1H), 6.71 (dd, J = 8.0, 2.1 Hz, 1H), 6.67 (t, J = 2.3 Hz, 1H), 5.89 (s, 1H), 5.13–5.10 (m, 1H), 3.80 (s, 3H), 2.25–2.22 (m, 7H), 1.71 (s, 3H), 1.63 (s, 3H). ^13^C NMR (126 MHz, Chloroform-d) δ 165.0, 163.4, 160.5, 151.7, 132.8, 129.7, 122.9, 114.7, 114.1, 111.5, 107.8, 55.4, 41.2, 26.1, 25.7, 19.2, 17.7. HRMS (ESI) *m*/*z* calcd for C_17_H_23_O_3_ [M + H] ^+^: 275.1642; found, 275.1642.

*4-methoxyphenyl (E)-3,7-dimethylocta-2,6-dienoate* (**5g**), colorless liquid, 56.1% yield. ^1^H NMR (500 MHz, Chloroform-d) δ 7.03–7.01 (m, 2H), 6.90–6.88 (m, 2H), 5.89 (s, 1H), 5.13–5.10 (m, 1H), 3.80 (s, 3H), 2.24–2.21 (m, 7H), 1.71 (s, 3H), 1.63 (s, 3H). ^13^C NMR (126 MHz, Chloroform-d) δ 165.5, 163.0, 157.1, 144.2, 132.7, 122.9, 122.6, 114.7, 114.4, 55.6, 41.2, 26.1, 25.7, 19.2, 17.7. HRMS (ESI) *m*/*z* calcd for C_17_H_23_O_3_ [M + H] ^+^: 275.1642; found, 275.1641.

*2-chlorophenyl (E)-3,7-dimethylocta-2,6-dienoate* (**5h**), colorless liquid, 60.9% yield. ^1^H NMR (500 MHz, Chloroform-d) δ 7.44 (dd, J = 8.0, 1.6 Hz, 1H), 7.28 (td, J = 7.7, 1.6 Hz, 1H), 7.17 (ddd, J = 13.9, 7.8, 1.6 Hz, 2H), 5.97 (q, J = 1.3 Hz, 1H), 5.14–5.09 (m, 1H), 2.29–2.21 (m, 7H), 1.71 (d, J = 1.4 Hz, 3H), 1.64 (d, J = 1.4 Hz, 3H). ^13^C NMR (126 MHz, Chloroform-d) δ 164.4, 164.0, 147.1, 132.9, 130.2, 127.7, 127.2, 126.7, 124.0, 122.8, 114.0, 41.2, 26.1, 25.7, 19.3, 17.8. HRMS (ESI) *m*/*z* calcd for C_16_H_20_ClO_2_ [M + H] ^+^: 279.1146; found, 279.1146.

*3-chlorophenyl (E)-3,7-dimethylocta-2,6-dienoate* (**5i**), colorless liquid, 54.2% yield. ^1^H NMR (500 MHz, Chloroform-d) δ 7.30 (t, J = 8.1 Hz, 1H), 7.20 (ddd, J = 8.1, 2.0, 1.0 Hz, 1H), 7.15 (t, J = 2.1 Hz, 1H), 7.02 (ddd, J = 8.2, 2.2, 1.0 Hz, 1H), 5.88 (q, J = 1.2 Hz, 1H), 5.11 (tq, J = 5.5, 1.5 Hz, 1H), 2.28–2.21 (m, 7H), 1.71 (d, J = 1.4 Hz, 3H), 1.64 (d, J = 1.4 Hz, 3H). ^13^C NMR (126 MHz, Chloroform-d) δ 159.8, 159.5, 146.5, 129.8, 128.1, 125.3, 121.0, 118.0, 117.8, 115.5, 109.5, 36.5, 21.3, 21.0, 14.5, 13.0. HRMS (ESI) *m*/*z* calcd for C_16_H_20_ClO_2_ [M + H] ^+^: 279.1146; found, 279.1146.

*4-chlorophenyl (E)-3,7-dimethylocta-2,6-dienoate* (**5j**), colorless liquid, 59.1% yield. ^1^H NMR (500 MHz, Chloroform-d) δ 7.35–7.32 (m, 2H), 7.07–7.04 (m, 2H), 5.88 (q, J = 1.3 Hz, 1H), 5.13–5.08 (m, 0H), 2.28–2.20 (m, 7H), 1.71 (d, J = 1.5 Hz, 3H), 1.63 (d, J = 1.4 Hz, 3H). ^13^C NMR (126 MHz, Chloroform-d) δ 156.0, 159.3, 144.5, 128.1, 126.1, 118.4, 118.0, 109.6, 36.5, 21.3, 21.0, 14.5, 13.0. HRMS (ESI) *m*/*z* calcd for C_16_H_20_ClO_2_ [M + H] ^+^: 279.1146; found, 279.1145.

*2-fluorophenyl (E)-3,7-dimethylocta-2,6-dienoate* (**5k**), colorless liquid, 57.1% yield. ^1^H NMR (500 MHz, Chloroform-d) δ 7.20–7.13 (m, 4H), 5.95 (q, J = 1.3 Hz, 1H), 5.14–5.09 (m, 1H), 2.28–2.22 (m, 7H), 1.71 (d, J = 1.5 Hz, 3H), 1.64 (d, J = 1.3 Hz, 3H). ^13^C NMR (126 MHz, Chloroform-d) δ 164.5, 163.9, 154.4 (d, J = 249.5 Hz), 138.2 (d, J = 13.4 Hz), 132.9, 126.8 (d, J = 7.5 Hz), 124.4 (d, J = 4.3 Hz), 124.1, 122.8, 116.6 (d, J = 19.1 Hz), 113.8, 41.2, 26.0, 25.7, 19.3, 17.7. HRMS (ESI) *m*/*z* calcd for C_16_H_20_FO_2_ [M + H] ^+^: 263.1442; found, 263.1446.

*3-fluorophenyl (E)-3,7-dimethylocta-2,6-dienoate* (**5l**), colorless liquid, 63.9% yield. ^1^H NMR (500 MHz, Chloroform-d) δ 7.33 (td, J = 8.3, 6.5 Hz, 1H), 6.95–6.90 (m, 2H), 6.88 (dt, J = 9.6, 2.3 Hz, 1H), 5.88 (q, J = 1.2 Hz, 1H), 5.13–5.09 (m, 1H), 2.29–2.20 (m, 7H), 1.71 (d, J = 1.5 Hz, 3H), 1.64 (d, J = 1.4 Hz, 3H). ^13^C NMR (126 MHz, Chloroform-d) δ 164.5, 164.2, 162.9 (d, J = 246.5 Hz), 151.7 (d, J = 11.3 Hz), 132.8, 130.0 (d, J = 9.1 Hz), 122.7, 117.6 (d, J = 3.3 Hz), 114.3, 112.5 (d, J = 21.4 Hz), 109.9 (d, J = 24.8 Hz), 41.2, 26.0, 25.7, 19.2, 17.7. HRMS (ESI) *m*/*z* calcd for C_16_H_19_FNaO_2_ [M + Na] ^+^: 285.1261; found, 285.1264.

*4-fluorophenyl (E)-3,7-dimethylocta-2,6-dienoate* (**5m**), colorless liquid, 68.6% yield. ^1^H NMR (500 MHz, Chloroform-d) δ 7.07–7.05 (m, 4H), 5.89 (q, J = 1.2 Hz, 1H), 5.13–5.09 (m, 1H), 2.25–2.21 (m, 7H), 1.71 (d, J = 1.4 Hz, 3H), 1.63 (d, J = 1.4 Hz, 3H). ^13^C NMR (126 MHz, Chloroform-d) δ 165.1, 163.8, 160.1 (d, J = 244.1 Hz), 146.5, 132.8, 123.2 (d, J = 8.1 Hz), 122.8, 115.9 (d, J = 23.7 Hz), 114.4, 41.2, 26.0, 25.7, 19.2, 17.7. HRMS (ESI) *m*/*z* calcd for C_16_H_19_FNaO_2_ [M + Na]^+^: 285.1261; found, 285.1267.

*2-nitrophenyl (E)-3,7-dimethylocta-2,6-dienoate* (**5n**), yellow liquid, 62.3% yield. ^1^H NMR (500 MHz, Chloroform-d) δ 8.08 (dd, J = 8.2, 1.6 Hz, 1H), 7.65 (td, J = 7.8, 1.6 Hz, 1H), 7.38 (ddd, J = 8.5, 7.5, 1.4 Hz, 1H), 7.26 (dd, J = 8.1, 1.4 Hz, 1H), 5.96 (q, J = 1.3 Hz, 1H), 5.14–5.09 (m, 1H), 2.29–2.21 (m, 6H), 1.72 (d, J = 1.5 Hz, 3H), 1.64 (d, J = 1.4 Hz, 3H). ^13^C NMR (126 MHz, Chloroform-d) δ 165.8, 163.8, 144.2, 142.3, 134.5, 133.0, 126.2, 125.6, 125.5, 122.7, 113.6, 41.3, 26.0, 25.7, 19.5, 17.8. HRMS (ESI) *m*/*z* calcd for C_16_H_18_NO_4_ [M − H]^−^: 288.1241; found, 288.1238.

*3-nitrophenyl (E)-3,7-dimethylocta-2,6-dienoate* (**5o**), yellow liquid, 75.8% yield. ^1^H NMR (500 MHz, Chloroform-d) δ 8.10 (ddd, J = 8.1, 2.2, 1.0 Hz, 1H), 8.02 (t, J = 2.2 Hz, 1H), 7.56 (t, J = 8.2 Hz, 1H), 7.47 (ddd, J = 8.1, 2.3, 1.1 Hz, 1H), 5.92 (q, J = 1.3 Hz, 1H), 5.13–5.09 (m, 1H), 2.29–2.22 (m, 7H), 1.72 (d, J = 1.4 Hz, 3H), 1.64 (d, J = 1.3 Hz, 3H). ^13^C NMR (126 MHz, Chloroform-d) δ 165.6, 164.1, 151.2, 148.8, 133.0, 129.9, 128.4, 122.6, 120.4, 117.6, 113.8, 41.3, 26.0, 25.7, 19.4, 17.8. HRMS (ESI) *m*/*z* calcd for C_16_H_18_NO_4_ [M − H]^−^: 288.1241; found, 288.1238.

*4-nitrophenyl (E)-3,7-dimethylocta-2,6-dienoate* (**5p**), white solid, m.p. 61.8-62.2 °C 5.8% yield. ^1^H NMR (500 MHz, Chloroform-d) δ 8.31–8.24 (m, 2H), 7.34–7.27 (m, 2H), 5.91 (q, J = 1.2 Hz, 1H), 5.13–5.09 (m, 1H), 2.29–2.22 (m, 7H), 1.72 (d, J = 1.4 Hz, 3H), 1.64 (d, J = 1.4 Hz, 3H). ^13^C NMR (126 MHz, Chloroform-d) δ 165.8, 163.7, 155.8, 145.1, 133.0, 125.1, 122.6, 122.6, 113.83, 41.3, 26.0, 25.7, 19.4, 17.8. HRMS (ESI) *m*/*z* calcd for C_16_H_18_NO_4_ [M − H]^−^: 288.1241; found, 288.1236.

*2-bromophenyl (E)-3,7-dimethylocta-2,6-dienoate* (**5q**), colorless liquid, 55.2% yield. ^1^H NMR (500 MHz, Chloroform d) δ 7.60 (dd, J = 8.0, 1.5 Hz, 1H), 7.31 (td, J = 7.7, 1.5 Hz, 1H), 7.14 (dd, J = 8.1, 1.5 Hz, 1H), 7.09 (td, J = 7.7, 1.6 Hz, 1H), 5.96 (q, J = 1.3 Hz, 1H), 5.13–5.10 (m, 1H), 2.28–2.21 (m, 5H), 1.71 (d, J = 1.5 Hz, 3H), 1.63 (d, J = 1.6 Hz, 3H). ^13^C NMR (126 MHz, Chloroform-d) δ 164.5, 164.0, 148.4, 133.3, 132.8, 128.4, 127.0, 124.1, 122.8, 116.5, 114.1, 41.3, 26.1, 25.7, 19.4, 17.8. HRMS (ESI) *m*/*z* calcd for C_16_H_19_BrNaO_2_ [M + Na]^+^: 345.0461; found, 345.0465.

*3-bromophenyl (E)-3,7-dimethylocta-2,6-dienoate* (**5r**), colorless liquid, 63.7% yield. ^1^H NMR (500 MHz, Chloroform-d) δ 7.35 (ddd, J = 8.0, 1.9, 1.0 Hz, 1H), 7.31 (t, J = 2.0 Hz, 1H), 7.23 (d, J = 8.1 Hz, 1H), 7.06 (ddd, J = 8.2, 2.2, 1.0 Hz, 1H), 5.88 (q, J = 1.2 Hz, 1H), 5.11 (tt, J = 5.5, 1.4 Hz, 1H), 2.26–2.21 (m, 4H), 1.71 (d, J = 1.4 Hz, 3H), 1.63 (d, J = 1.4 Hz, 3H). ^13^C NMR (126 MHz, Chloroform-d) δ 164.5, 164.3, 151.3, 132.8, 130.4, 128.7, 125.3, 122.7, 122.3, 120.7, 114.2, 41.2, 26.0, 25.7, 19.3, 17.8. HRMS (ESI) *m*/*z* calcd for C_16_H_19_BrNaO_2_ [M + Na]^+^: 345.0461; found, 345.0461.

*4-bromophenyl (E)-3,7-dimethylocta-2,6-dienoate* (**5s**), colorless liquid, 60.4% yield. ^1^H NMR (500 MHz, Chloroform-d) δ 7.50–7.47 (m, 2H), 7.02–6.98 (m, 2H), 5.88 (q, J = 1.2 Hz, 1H), 5.13–5.08 (m, 1H), 2.27–2.20 (m, 7H), 1.71 (d, J = 1.4 Hz, 3H), 1.63 (d, J = 1.4 Hz, 3H). ^13^C NMR (126 MHz, Chloroform-d) δ 164.6, 164.1, 149.8, 132.8, 132.4, 123.7, 122.8, 118.5, 114.3, 41.2, 26.0, 25.7, 19.3, 17.8. HRMS (ESI) *m*/*z* calcd for C_16_H_19_BrNaO_2_ [M + Na]^+^: 345.0461; found, 345.0464.

*2-(trifluoromethoxy)phenyl (E)-3,7-dimethylocta-2,6-dienoate* (**5t**), colorless liquid, 60.2% yield. ^1^H NMR (500 MHz, Chloroform-d) δ 7.34–7.30 (m, 2H), 7.26–7.21 (m, 2H), 5.93 (q, J = 1.3 Hz, 1H), 5.13–5.09 (m, 1H), 2.27–2.21 (m, 7H), 1.71 (d, J = 1.4 Hz, 3H), 1.64 (d, J = 1.3 Hz, 3H). ^13^C NMR (126 MHz, Chloroform-d) δ 164.6, 163.9, 142.9, 141.0, 132.9, 127.6, 126.5, 124.5, 122.7, 122.4, 120.5 (d, J = 257.8 Hz), 113.8, 41.2, 26.0, 25.7, 19.3, 17.7. HRMS (ESI) *m*/*z* calcd for C_17_H_18_F_3_O_3_ [M − H]^−^: 327.1214; found, 327.1207.

*3-(trifluoromethoxy)phenyl (E)-3,7-dimethylocta-2,6-dienoate* (**5u**), colorless liquid, 57.8% yield. ^1^H NMR (500 MHz, Chloroform-d) δ 7.39 (t, J = 8.2 Hz, 1H), 7.10–7.07 (m, 2H), 7.04–7.02 (m, 1H), 5.89 (q, J = 1.3 Hz, 1H), 5.13–5.09 (m, 1H), 2.27–2.21 (m, 7H), 1.71 (d, J = 1.4 Hz, 3H), 1.64 (d, J = 1.5 Hz, 3H). ^13^C NMR (126 MHz, Chloroform-d) δ 164.5, 164.4, 151.5, 149.6, 132.9, 130.0, 122.7, 120.4 (d, J = 257.4 Hz), 120.4, 117.8, 115.2, 114.2, 41.2, 26.0, 25.7, 19.3, 17.7. HRMS (ESI) *m*/*z* calcd for C_17_H_18_F_3_O_3_ [M − H]^−^: 327.1214; found, 327.1209.

*4-(trifluoromethoxy)phenyl (E)-3,7-dimethylocta-2,6-dienoate* (**5v**), colorless liquid, 62.1% yield. ^1^H NMR (500 MHz, Chloroform-d) δ 7.24–7.22 (m, 2H), 7.15–7.12 (m, 2H), 5.90 (q, J = 1.2 Hz, 1H), 5.13–5.09 (m, 1H), 2.26–2.21 (m, 7H), 1.71 (d, J = 1.5 Hz, 3H), 1.64 (d, J = 1.4 Hz, 3H). ^13^C NMR (126 MHz, Chloroform-d) δ 164.7, 164.3, 149.0, 146.3, 132.9, 123.1, 122.7, 122.0, 120.5 (d, J = 257.6 Hz), 114.3, 41.2, 26.0, 25.7, 19.3, 17.7. HRMS (ESI) *m*/*z* calcd for C_17_H_18_F_3_O_3_ [M - H]^−^: 327.1214; found, 327.1209.

*2,6-difluorophenyl (E)-3,7-dimethylocta-2,6-dienoate* (**5w**), colorless liquid, 55.6% yield. ^1^H NMR (500 MHz, Chloroform-d) δ 7.18–7.12 (m, 1H), 7.00–6.94 (m, 2H), 5.98 (q, J = 1.2 Hz, 1H), 5.13–5.09 (m, 1H), 2.29–2.22 (m, 7H), 1.71 (d, J = 1.4 Hz, 3H), 1.63 (d, J = 1.4 Hz, 3H). ^13^C NMR (126 MHz, Chloroform-d) δ 165.6, 162.8, 155.6 (dd, J = 250.7, 4.4 Hz), 133.0, 127.3 (t, J = 15.9 Hz), 126.0 (t, J = 9.1 Hz), 122.7, 113.1, 112.1 (d, J = 4.6 Hz), 111.9 (d, J = 4.5 Hz), 41.3, 26.0, 25.7, 19.4, 17.7. HRMS (ESI) *m*/*z* calcd for C_16_H_18_F_2_NaO_2_ [M + Na]^−^: 303.1167; found, 303.1171.

*2,6-dibromophenyl (E)-3,7-dimethylocta-2,6-dienoate* (**5x**), colorless liquid, 49.5% yield. ^1^H NMR (500 MHz, Chloroform-d) δ 7.54 (d, J = 8.1 Hz, 2H), 6.96 (t, J = 8.0 Hz, 1H), 6.00 (q, J = 1.2 Hz, 1H), 5.14–5.09 (m, 1H), 2.29–2.22 (m, 7H), 1.71 (d, J = 1.5 Hz, 3H), 1.63 (d, J = 1.6 Hz, 3H). ^13^C NMR (126 MHz, Chloroform-d) δ 165.39, 162.60, 146.47, 132.91, 132.32, 127.85, 122.7, 118.2, 113.6, 41.3, 26.0, 25.7, 19.5, 17.8. HRMS (ESI) *m*/*z* calcd for C_16_H_18_Br_2_NaO_2_ [M + Na]^−^: 422.9566; found, 422.9566.

### 3.3. Repellent Activity Test of Compounds **5a**–**5x**

The behavioral response of *A. pisum* to **5a**–**5x** was investigated with a glass two-way olfactometer [36], and the aphid-repellent activity was carried out according to our previously published method [37]. The aphids were reared in a growth chamber (at 23 ± 1 °C, with a 16L:8D photoperiod and 70% relative humidity) at China Agricultural University. The compounds were weighed, dissolved in n-hexane, and prepared into a test solution with a concentration of 2000 mg/L using n-hexane as a blank control. About 20 wingless *A. pisum* were placed through the release port and each arm was pumped with 0.2 L/min of activated carbon and distilled water. One arm was chosen as the “treatment” (T) arm and another was the “control” (C) arm. For the “T” arm, 2.5 µL of test solutions were applied to filter paper strips (1 cm^2^ diameter), and 2.5 µL of n-hexane was applied for the “C” arm.

The number of aphids in each direction was recorded for 15 min at 2 cm from the center of the horizontal arm. The olfactometer was washed with ethanol, the filter paper was replaced, and the two arms were switched and the experiment was repeated four times for each treatment. The repellent activity was evaluated using RP as the formula RP = (C − T)/(C+T) × 100%, where T and C mean the number of aphids in the treatment and control arm, respectively. The RP values of the derivatives were statistically analyzed with SPSS 24.0 (SPSS Inc., Chicago, IL, USA) using a one-way analysis of variance (ANOVA), followed by a Duncan’s test with a significance set at *p* < 0.05.

### 3.4. Competitive Fluorescence Binding Assays

The protein of ApisOBP9 was expressed and purified in our previous work and used to determine binding affinity with title compounds using fluorescence competitive binding assays, which were performed according to our previously reported method [23]. An RF-6000 Spectrofluorophotometer (SHIMADZU, Kyoto, Japan) was used to determine the results of the binding assay and N-phenyl-1-naphthylamine (1-NPN) was chosen as the fluorescent probe. The excitation wavelength was 337 nm and the emission spectrum was recorded between 350 nm and 500 nm. The recombinant proteins prepared in Tris-HCl (50 mM, pH 8.0) were titrated with aliquots of 1 mM of 1-NPN to final concentrations ranging from 2 to 20 µM to measure the binding affinity. To further measure the binding affinity of ligands to ApisOBP9, the proteins and fluorescent probe at 2 µM were titrated with aliquots of 1 mM compounds. The binding constant (K_1-NPN_) of 1-NPN to ApisOBP9 was calculated by GraphPad Prism 9 software (GraphPad Software Inc, San Diego, CA, USA) according to the equation Ki = [IC_50_]/(1 + [1-NPN]/K_1-NPN_), where [1-NPN] is the free concentration of 1-NPN and K_1-NPN_ is the dissociation constant of the complex protein/1-NPN. The binding affinity of the target compounds were compared with that of SPSS Statistics version 24.0 using one way analysis of variance (ANOVA) followed by Duncan’s test at a significance of *p* < 0.05.

### 3.5. Molecular Docking

The structure of ApisOBP9 was modelled in our previous study [31]. Molecular docking studies between the ligands and ApisOBP9 were performed using the Surflex-Dock algorithm in Sybyl 7.3 software [38]. The representative compounds, **5e**, **5f**, **5g**, and **5w**, and the lead compound, **CAU15**, were selected as ligands. A suitable putative conformationof the ligand, named protomol, was rapidly generated under the Hammerhead scoring function with a surface-based molecular similarity mode and was visually analyzed by PyMOL (version 1.9.0) (http://www.pymol.org/, open access, 22 August 2022).

## 4. Conclusions

In summary, a series of novel geranic acid esters containing substituted aromatic rings were designed by inverting the ester groups of lead compounds. The key intermediates **2** and **3** were obtained by mild oxidation conditions. Then, all the target compounds were synthesized via condensation of intermediate **3** with substituted phenols. The results of the bioassay showed that the repellent activity and protein-binding affinity of the compounds were increased after ester group inversion. Particularly, compounds **5f** and **5i** showed good repellent activity against *A. pisum* and good binding affinity with ApisOBP9. Meanwhile, the structure–activity relationship revealed that the introduction of *meta-*substituents on the benzene ring facilitates the biological activity, and when halogens were introduced on the benzene ring, the introduction of Cl and Br was superior to the introduction of F for the improvement of biological activity. The further molecular docking study exhibited that the hydrogen bond formation and hydrophobic interactions were vital for the binding affinity with ApisOBP9. In addition, all the target compounds were predicted to be eco-friendly and more conducive to act as volatile chemical signals between aphids compared to the lead compounds. The present work provides valuable clues for the rational design of aphid behavioral control agents.

## Figures and Tables

**Table 1:** Repellent activity of compounds **5a–5x**.

Compd.	R	RP (%) *	Compd.	R	RP (%) *
**5a**	H	38.1 ± 2.38	**5o**	3-NO_2_	41.5 ± 1.49
**5b**	2-CH_3_	35.2 ± 2.65	**5p**	4-NO_2_	39.8 ± 1.95
**5c**	3-CH_3_	39.8 ± 1.95	**5q**	2-Br	42.7 ± 1.36
**5d**	4-CH_3_	38.7 ± 2.75	**5r**	3-Br	51.2 ± 1.94
**5e**	2-OCH_3_	37.4 ± 2.08	**5s**	4-Br	43.4 ± 1.13
**5f**	3-OCH_3_	55.6 ± 3.40	**5t**	2-OCF_3_	28.2 ± 1.55
**5g**	4-OCH_3_	43.6 ± 3.09	**5u**	3-OCF_3_	38.0 ± 2.54
**5h**	2-Cl	31.8 ± 1.31	**5v**	4-OCF_3_	24.9 ± 2.52
**5i**	3-Cl	51.4 ± 1.20	**5w**	2,6-F	36.7 ± 1.67
**5j**	4-Cl	38.7 ± 2.75	**5x**	2,6-Br	43.9 ± 2.09
**5k**	2-F	28.8 ± 1.31	**CAU13**	3-OCH_3_	41.0 ± 1.33
**5l**	3-F	34.9 ± 3.30	**CAU14**	4-Br	33.5 ± 2.55
**5m**	4-F	27.9 ± 2.85	**CAU15**	2,6-F	18.3 ± 0.84
**5n**	2-NO_2_	23.4 ± 2.35			

*: The repellent activity was estimated by the repellent proportion (RP), calculated by the formula RP = (C − T)/(C + T) × 100%, where C means those in the control arm and T indicates the number of aphids in the treatment arm. All the values are mean ± standard deviation.

**Table 2:** Binding affinity of target compounds with ApisOBP9.

Compd.	R	1/Ki (μM) *	Compd.	R	1/Ki (μM) *
**5a**	H	0.18 ± 0.011	**5o**	3-NO_2_	0.32 ± 0.018
**5b**	2-CH_3_	0.16 ± 0.004	**5p**	4-NO_2_	0.34 ± 0.011
**5c**	3-CH_3_	0.34 ± 0.011	**5q**	2-Br	0.35 ± 0.048
**5d**	4-CH_3_	0.36 ± 0.007	**5r**	3-Br	0.34 ± 0.028
**5e**	2-OCH_3_	0.31 ± 0.004	**5s**	4-Br	0.39 ± 0.018
**5f**	3-OCH_3_	0.49 ± 0.032	**5t**	2-OCF_3_	0.23 ± 0.004
**5g**	4-OCH_3_	0.26 ± 0.025	**5u**	3-OCF_3_	0.34 ± 0.032
**5h**	2-Cl	0.30 ± 0.028	**5v**	4-OCF_3_	0.30 ± 0.004
**5i**	3-Cl	0.44 ± 0.035	**5w**	2,6-F	0.37 ± 0.023
**5j**	4-Cl	0.26 ± 0.004	**5x**	2,6-Br	0.40 ± 0.006
**5k**	2-F	0.23 ± 0.004	**CAU 1** **3**	3-OCH_3_	0.32 ± 0.035
**5l**	3-F	0.32 ± 0.021	**CAU 1** **4**	4-Br	0.35 ± 0.026
**5m**	4-F	0.19 ± 0.010	**CAU 15**	2,6-F	0.19 ± 0.012
**5n**	2-NO_2_	0.25 ± 0.000			

* The binding affinity of lead compounds and **5a**–**5x** to ApisOBP9 were estimated by the Ki, calculated by the formula Ki = [IC_50_]/(1 + [1-NPN]/K_1-NPN_), where [1-NPN] is the free concentration of 1-NPN, and K_1-NPN_ is the dissociation constant of the complex protein/1-NPN.

**Table 3:** Effect of inversion of ester groups on vapor pressure and boiling point.

Substitution Groups	Vapor Pressure (mmHg)			Boiling Point (°C)		
	-O-C=O	O=C-O-	Delta (%) *	-O-C=O	O=C-O-	Delta (%) *
4-Br	7.081 × 10^−6^	7.846 × 10^−6^	+10.8	368.9	354.2	−4.0
2,6-F	1.874 × 10^−5^	3.513 × 10^−5^	+87.5	356.1	334.6	−6.1
3-OCH_3_	5.484 × 10^−6^	5.964 × 10^−6^	+8.8	373.3	334.7	−10.3

* Delta: rate of change of vapor pressure/boiling point before and after ester groups inversion, which calculated by the formula: (the value of **O=C-O-** − the value of **-O-C=O**)/the value of **-O-C=O** × 100%. “+” and “−” indicate increase and decrease, respectively.

**Table 4:** Honeybee toxicity, carcinogenicity, and rat oral toxicity prediction of lead compounds and the synthesized compounds.

Compd.	R	Honeybee Toxicity *^a^*	Carcinogenicity *^b^*	Oral Rat *^cd^* (LD_50_ mg/kg)
**CAU13**	-	low	inactive	8412.41
**CAU14**	-	low	inactive	1169.27
**CAU15**	-	low	inactive	304.10
**5a**	H	low	inactive	4175.84
**5b**	2-CH_3_	low	inactive	3196.92
**5c**	3-CH_3_	low	inactive	3216.81
**5d**	4-CH_3_	low	inactive	3256.71
**5e**	2-OCH_3_	low	inactive	6074.43
**5f**	3-OCH_3_	low	inactive	6139.49
**5g**	4-OCH_3_	low	inactive	5607.48
**5h**	2-Cl	low	inactive	3201.75
**5i**	3-Cl	low	inactive	3368.84
**5j**	4-Cl	low	inactive	3097.63
**5k**	2-F	low	inactive	2902.62
**5l**	3-F	low	inactive	3899.37
**5m**	4-F	low	inactive	3869.39
**5n**	2-NO_2_	low	inactive	2847.36
**5o**	3-NO_2_	low	inactive	2664.53
**5p**	4-NO_2_	low	inactive	3612.89
**5q**	2-Br	low	inactive	970.05
**5r**	3-Br	low	inactive	1061.77
**5s**	4-Br	low	inactive	1000.49
**5t**	2-OCF_3_	low	inactive	- *^e^*
**5u**	3-OCF_3_	low	inactive	- *^e^*
**5v**	4-OCF_3_	low	inactive	- *^e^*
**5w**	2,6-F	low	inactive	248.35
**5x**	2,6-Br	low	inactive	- *^e^*

*^a^* Predicted by the admetSAR. *^b^* Predicted by the ProTox-II. *^c^* Predicted by CompTox Chemicals Dashboard [the US Environmental Protection Agency (EPA)]. *^d^* US EPA pesticide toxicity classification standards (LD_50_): highly toxic-less than 50 mg/kg; moderately toxic-from 50 to 500 mg/kg; less toxic-from 500 to 5000 mg/kg; slightly toxic-more than 5000 mg/kg. *^e^* Not predicted.

## Data Availability

All data presented in this study are available in the article and in the Supplementary Material.

## Supplementary Materials

The following supporting information can be downloaded at: https://www.mdpi.com/article/10.3390/molecules27185949/s1, Scheme S1. Synthetic route of **CAU13**; Figure S1. Binding curves and Scatchard plots (insert) of 1-NPN to ApisOBP9; Figure S2. Competitive binding curves of compounds to ApisOBP9; ^1^H NMR and ^13^C NMR Spectrums of compounds **CAU13** and **5a**–**5x**; HRMS Spectrums of compounds **5a**–**5x**.

## Sample Availability

Samples of the compounds **5a**–**5x** are available from the authors.
