# Supplementary material for: Discovery of Novel Potential Aphid Repellents: Geranic Acid Esters Containing Substituted Aromatic Rings
Source: Molecules. 2022 Sep 13;27(18):5949. doi: 10.3390/molecules27185949 (PMC9502202; doi:10.3390/molecules27185949)

Supporting information

Discovery of Novel Potential Aphid Repellents: Geranic Acid Esters Containing Substituted Aromatic Rings

Shixiang Pan , Wenhao Li , Yaoguo Qin , Zhaokai Yang , Yan Liu , Zhuo Shi , Cheng Qu , Chen Luo * , and Xinling Yang *

Contents

1. Scheme S1. Synthetic route of **CAU13**……………………………………………3

2. Figure S1. Binding curves and Scatchard plots (insert) of 1-NPN to ApisOBP9 ….4

3. Figure S2. Competitive binding curves of compounds to ApisOBP9………………4

4. ^{1}H NMR and ^{13}C NMR Spectrums of compounds **CAU13** and **5a-5x** …………….5

5. HRMS Spectrums of compounds **5a-5x** ……………………………………………30

1. **Scheme S1**. Synthetic route of **CAU13.**

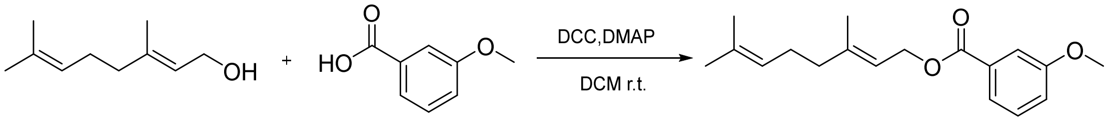

The synthesis of **CAU13** was started by stirring geraniol (5 mmol) with 3-Methoxy-benzoic acid (5 mmol) in DCM (30 mL) under ice bath. Then dicyclohexylcarbodiimide (DCC, 6 mmol) and 4-dimethylaminopyridine (DMAP, 0.6 mmol) were added into reaction stirring for 30 minutes. After that removed the ice bath and the mixture was finished by TLC detected after stirring 6 h, washed with saturated aqueous sodium bicarbonate solution for 3 times, extracted with DCM, dried with anhydrous sodium sulfate, and filtered. The organic phase was concentrated under reduced pressure and purified by column chromatography to obtain it as colorless liquid, 90.1% yield. ^{1}H NMR (500 MHz, Chloroform-*d*) δ 7.64 (dd, J = 7.6, 1.3 Hz, 1H), 7.57 (t, J = 2.0 Hz, 1H), 7.33 (t, J = 8.0 Hz, 1H), 7.09 (dd, J = 8.2, 2.7 Hz, 1H), 5.47 (tt, J = 7.1, 1.4 Hz, 1H), 5.12 – 5.08 (m, 1H), 4.84 (d, J = 7.0 Hz, 2H), 3.85 (s, 3H), 2.14 – 2.06 (m, 4H), 1.77 (s, 3H), 1.68 (s, 3H), 1.60 (s, 3H). ^{13}C NMR (126 MHz, Chloroform-*d*) δ 166.6, 159.6, 142.4, 131.9, 129.3, 123.8, 122.0, 119.3, 114.1, 62.0, 55.4, 39.6, 26.3, 25.7, 17.7, 16.6.

2. **Figure S1**. Binding curves and Scatchard plots (insert) of probe to ApisOBP9

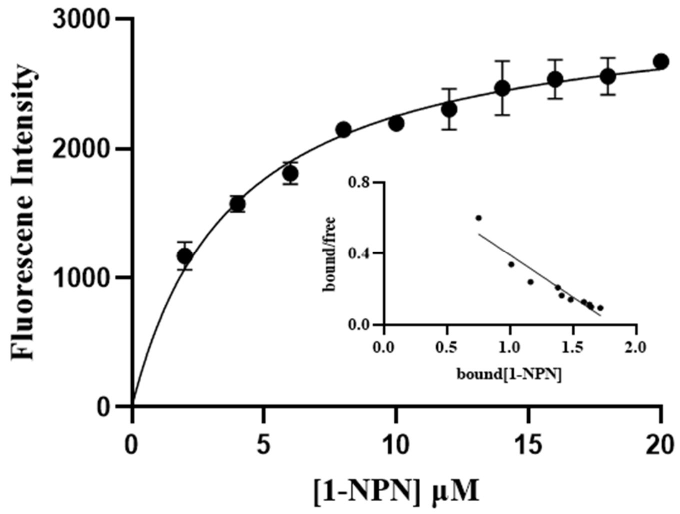

3. **Figure S2**. Competitive binding curves of compounds to ApisOBP9

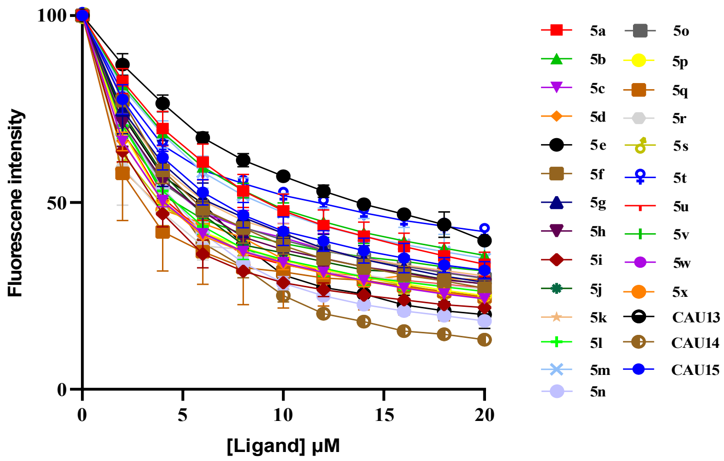

4. ^{1}H NMR and ^{13}C NMR Spectrums of compounds **CAU13** and **5a-5x**

^{1}H NMR of **CAU13**

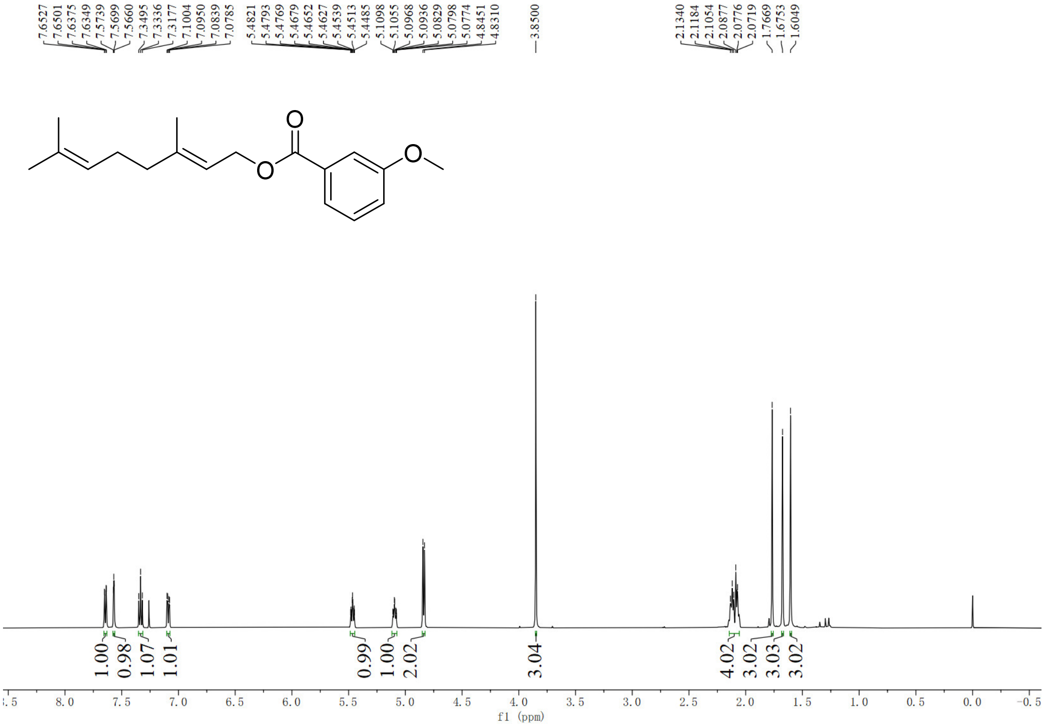

^{13}C NMR of **CAU13**

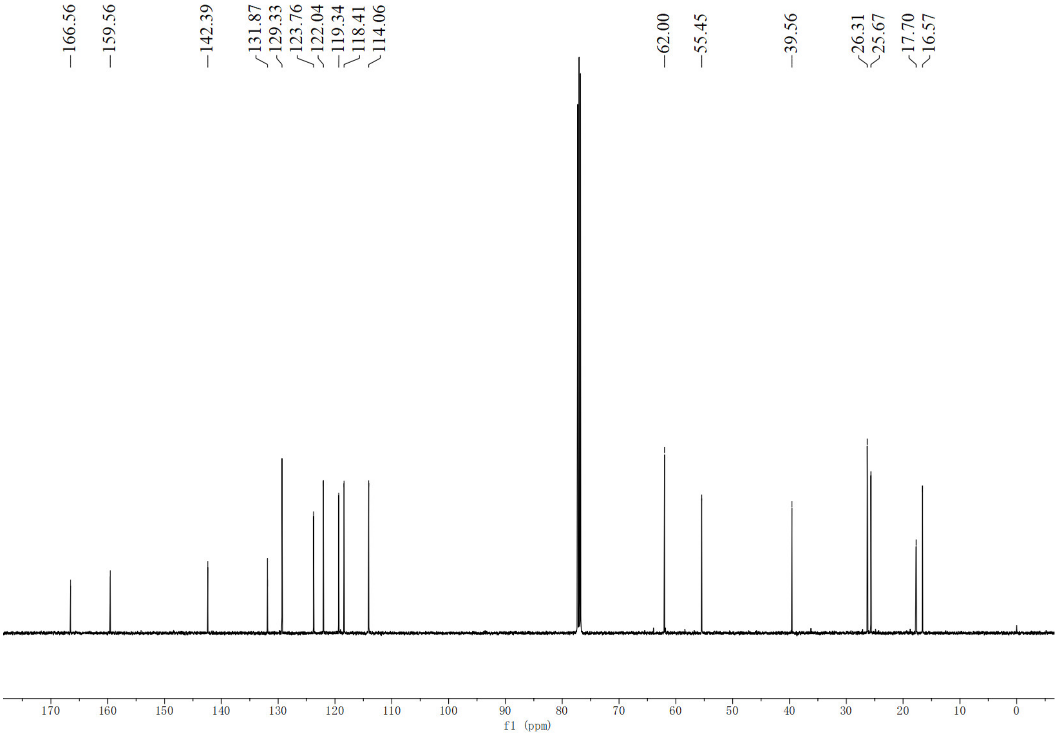

^{1}H NMR of **5a**

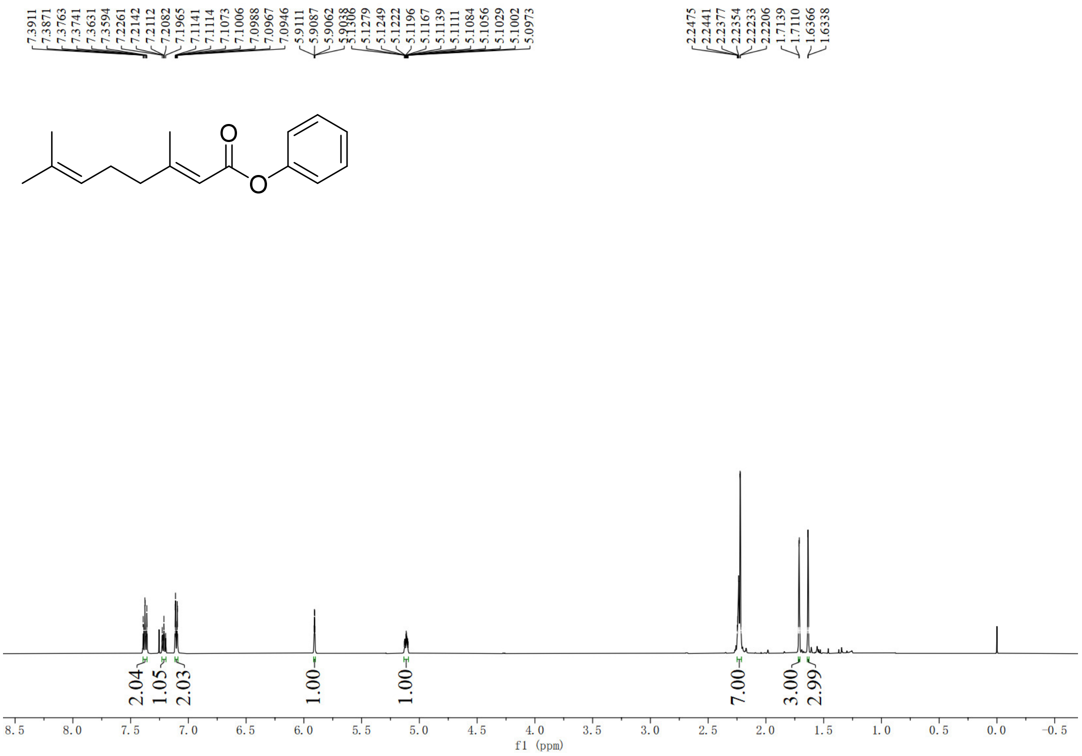

^{13}C NMR of **5a**

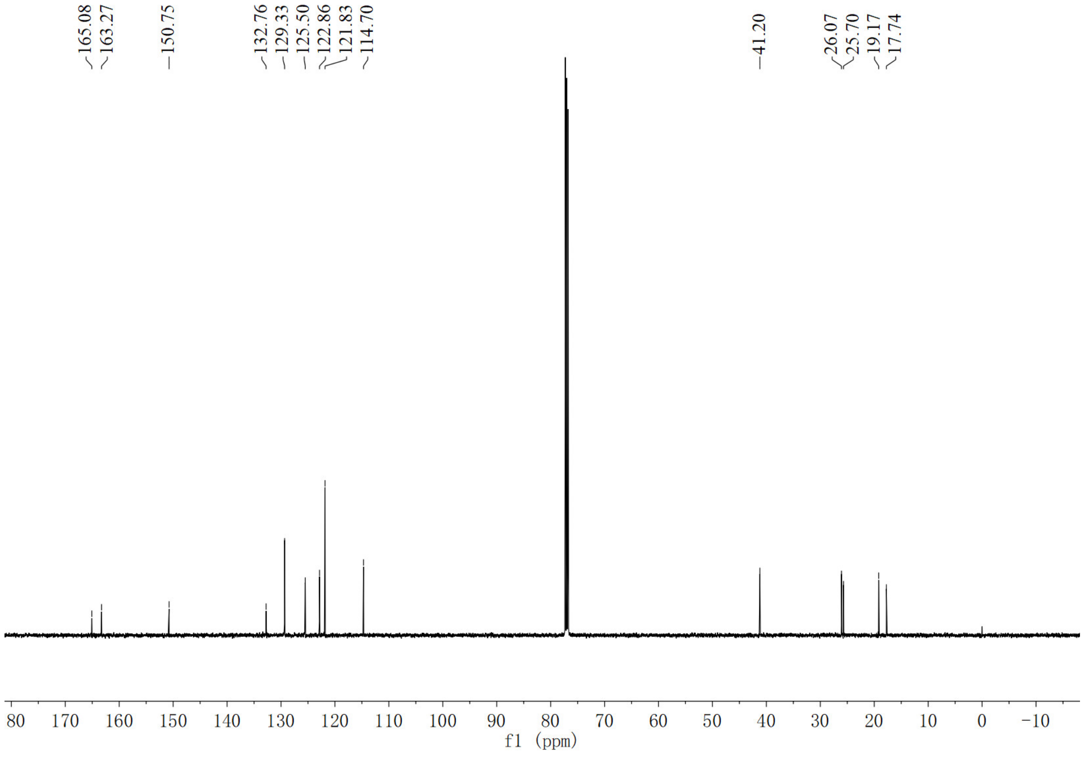

^{1}H NMR of **5b**

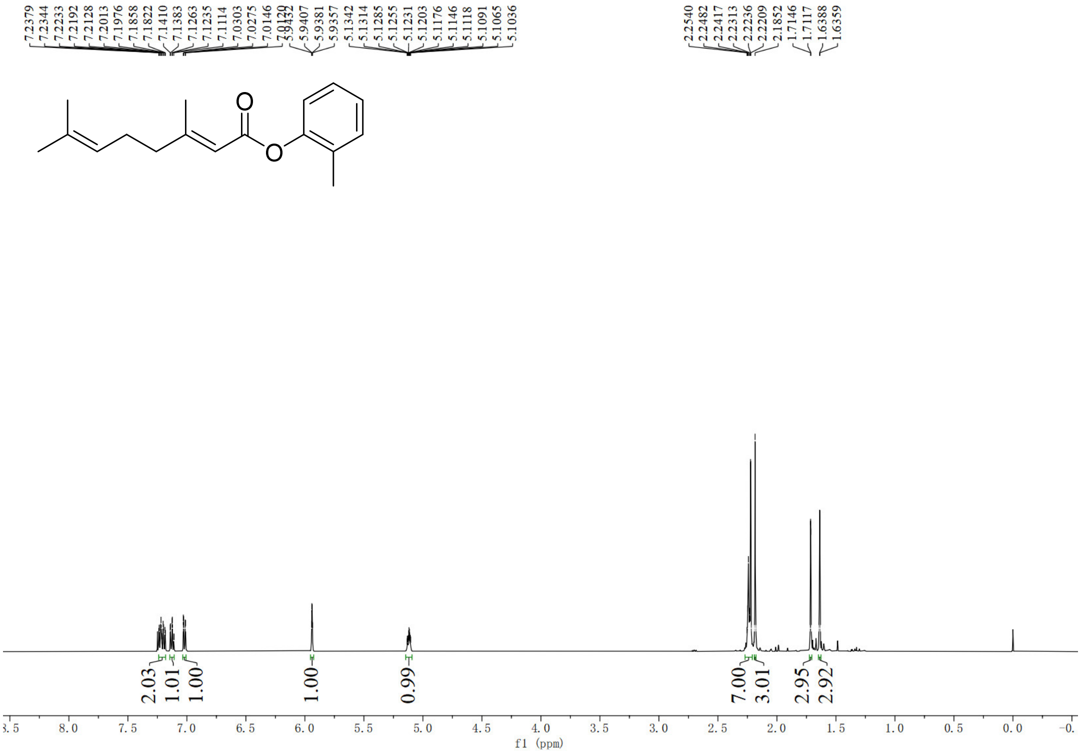

^{13}C NMR of **5b**

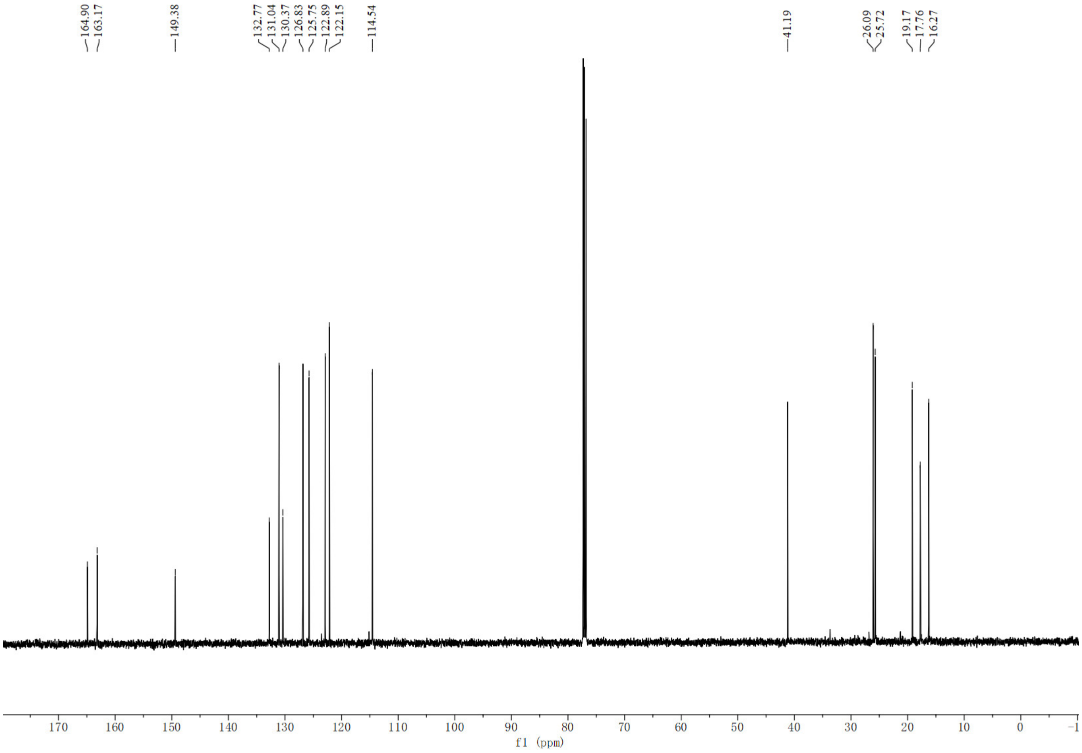

^{1}H NMR of **5c**

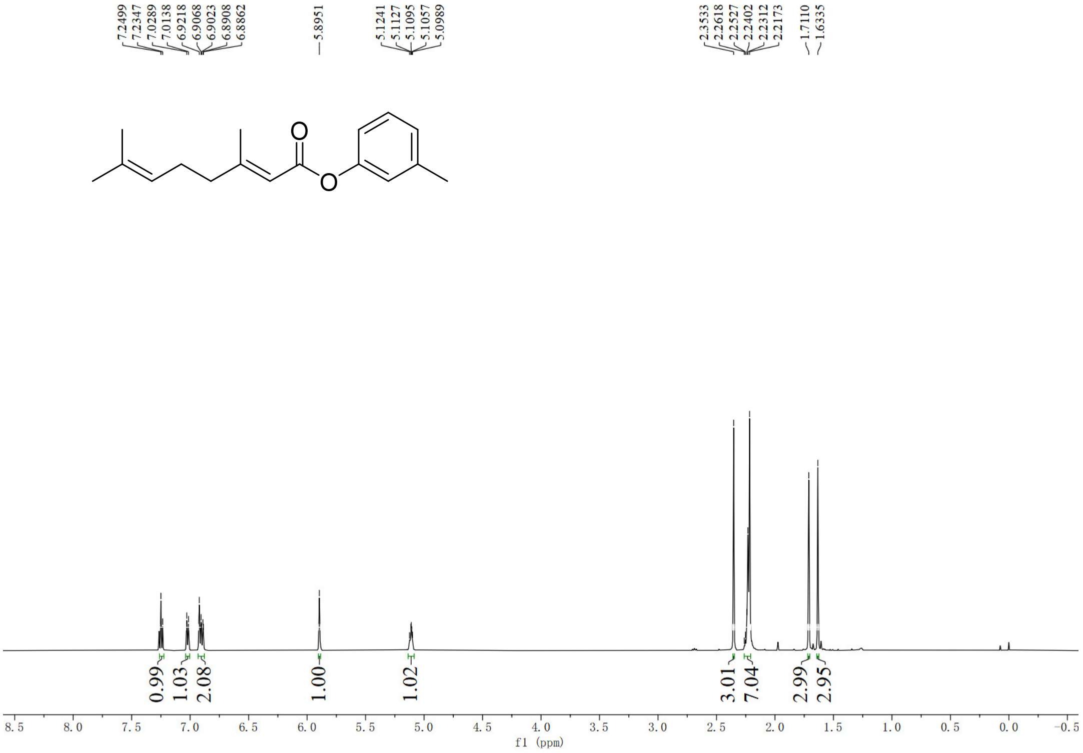

^{13}C NMR of **5c**

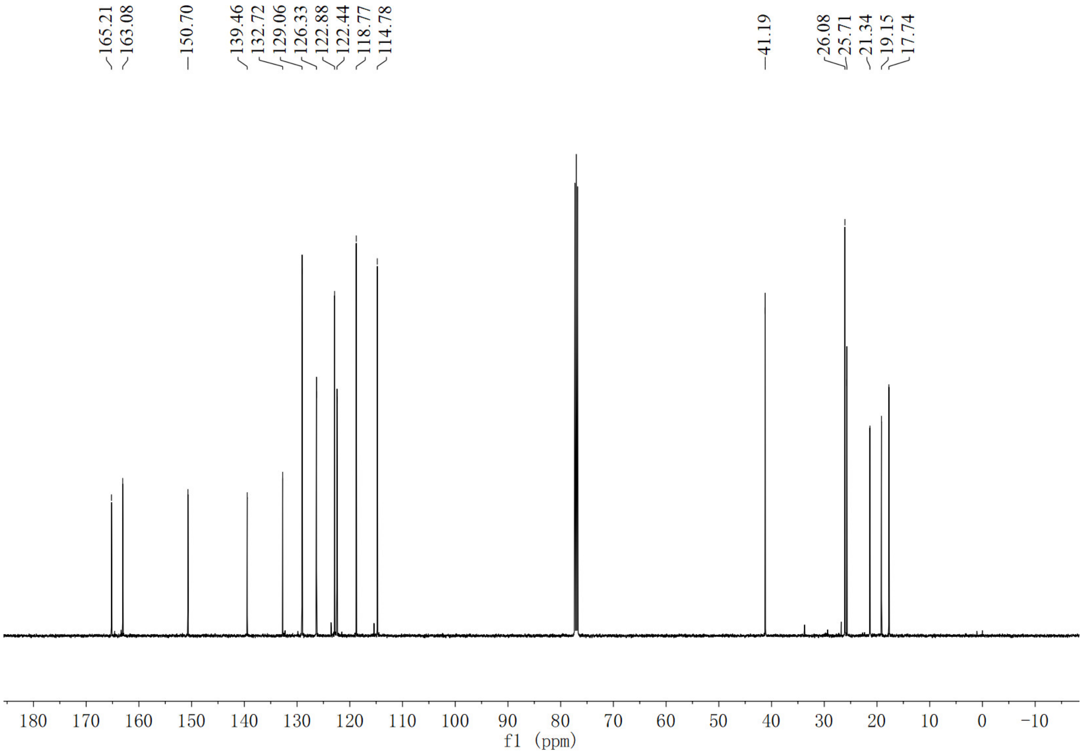

^{1}H NMR of **5d**

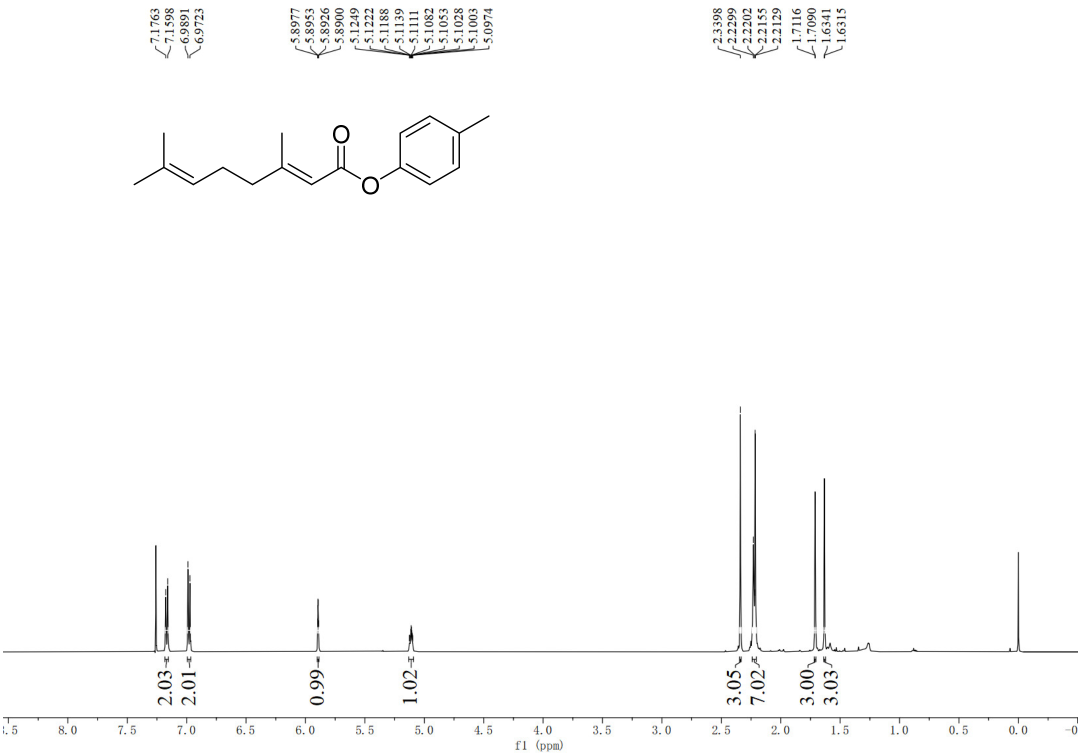

^{13}C NMR of **5d**

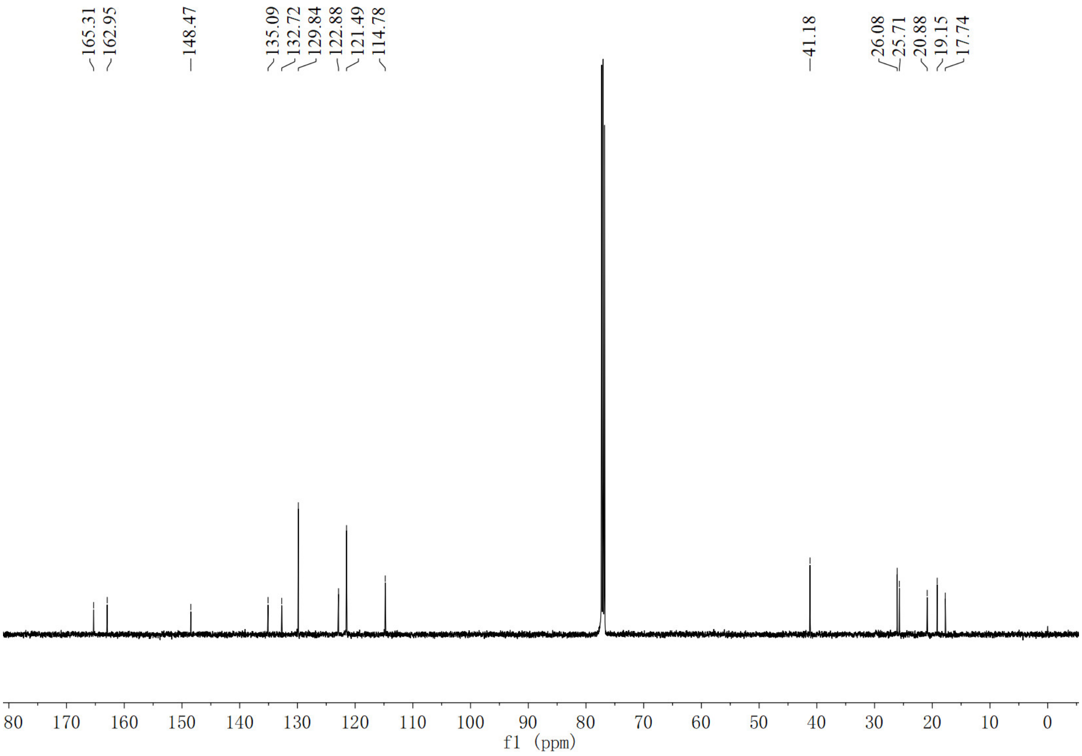

^{1}H NMR of **5e**

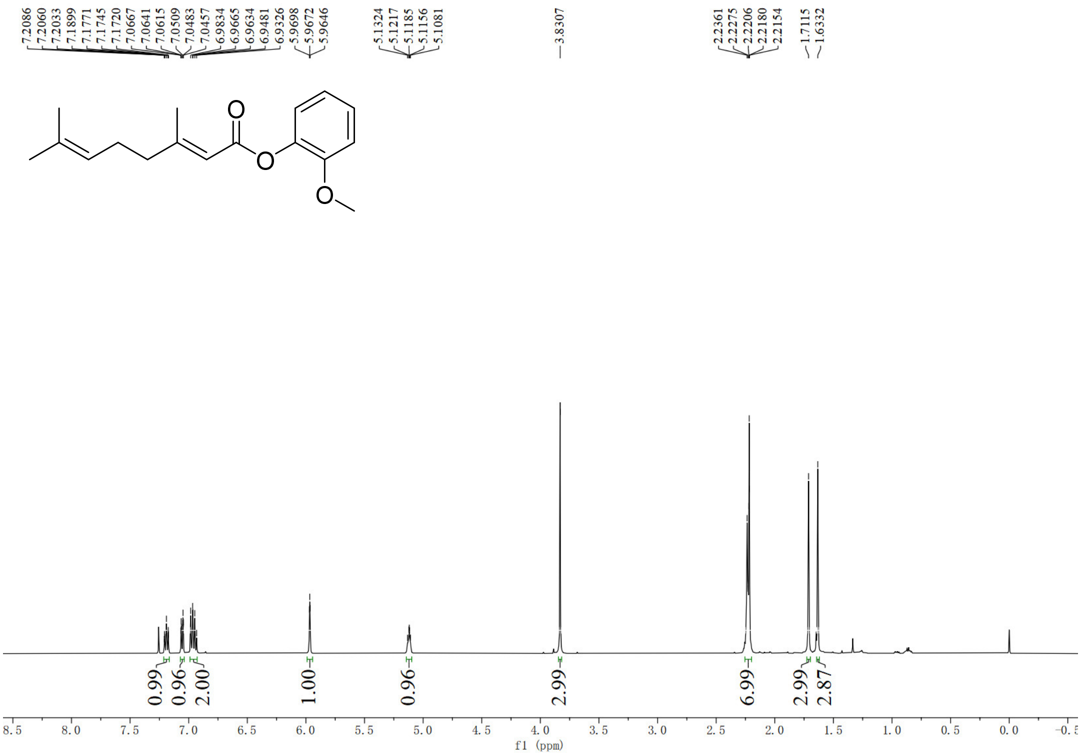

^{13}C NMR of **5e**

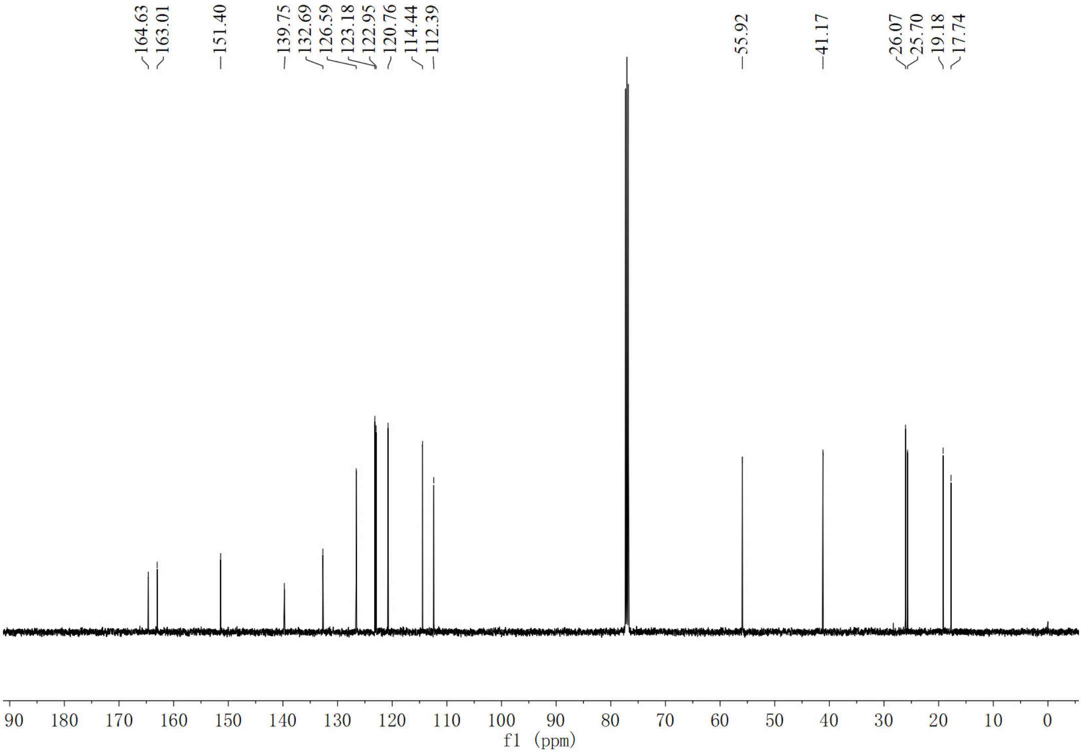

^{1}H NMR of **5f**

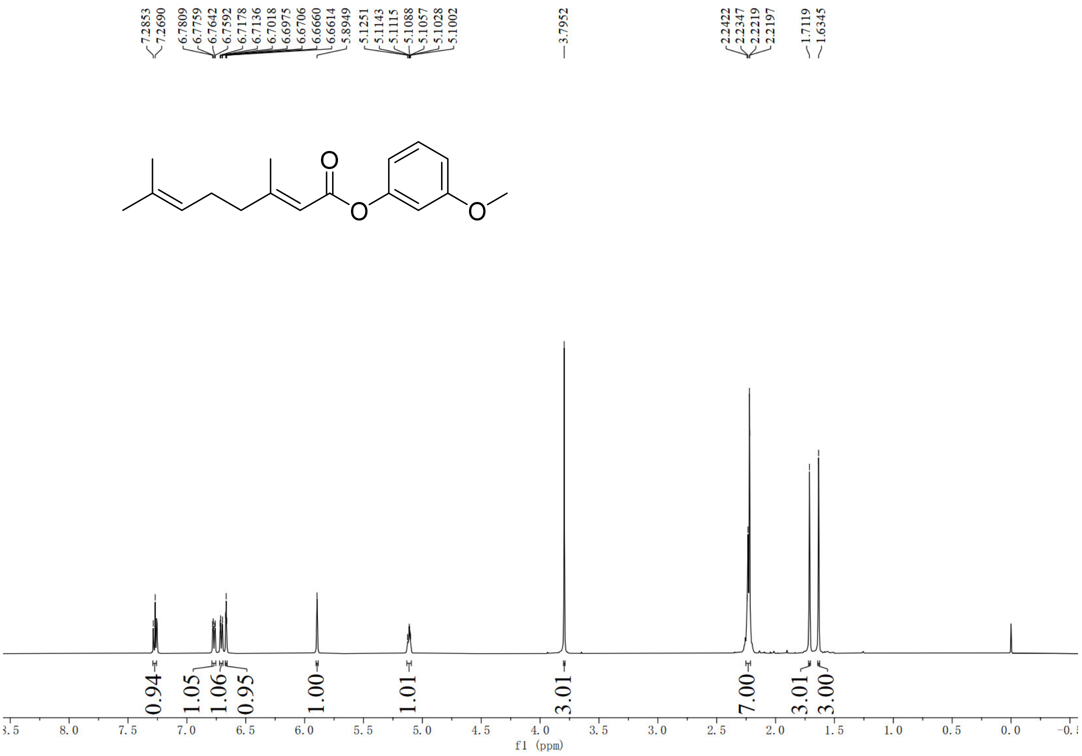

^{13}C NMR of **5f**

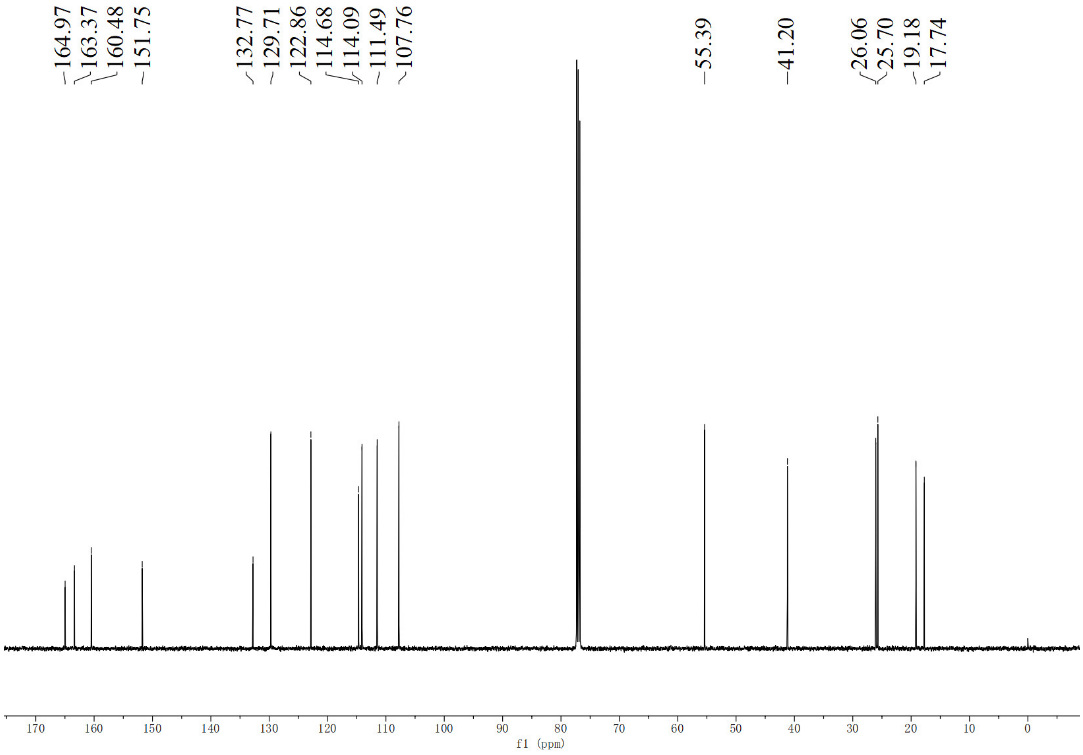

^{1}H NMR of **5g**

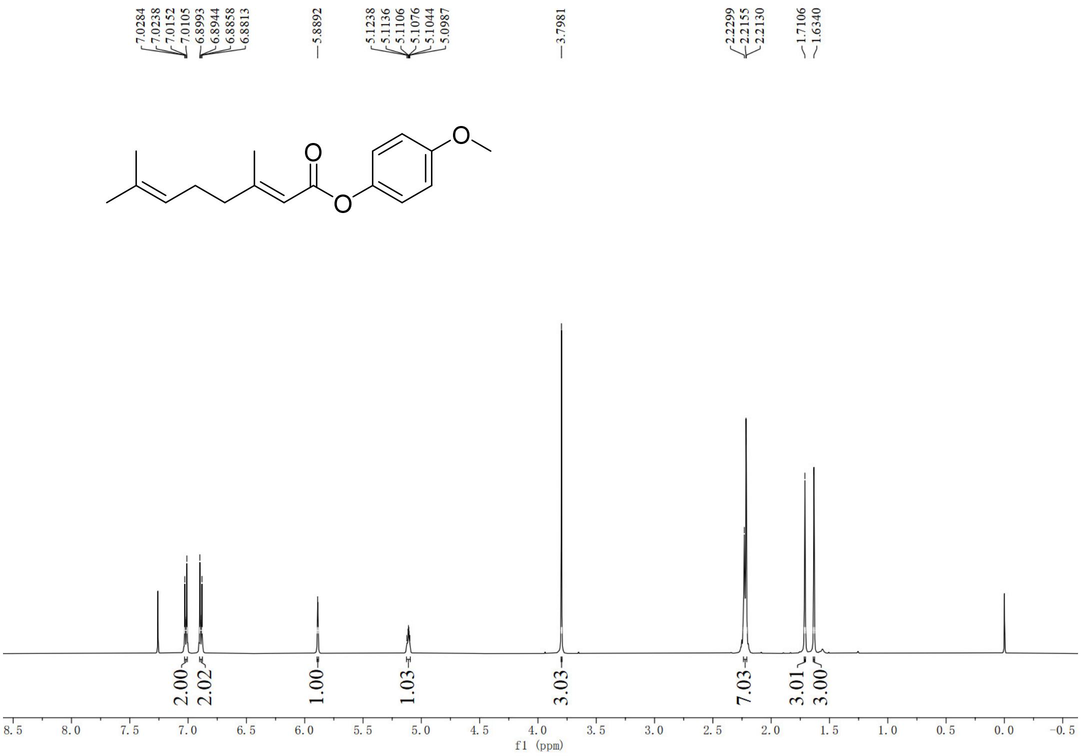

^{13}C NMR of **5g**

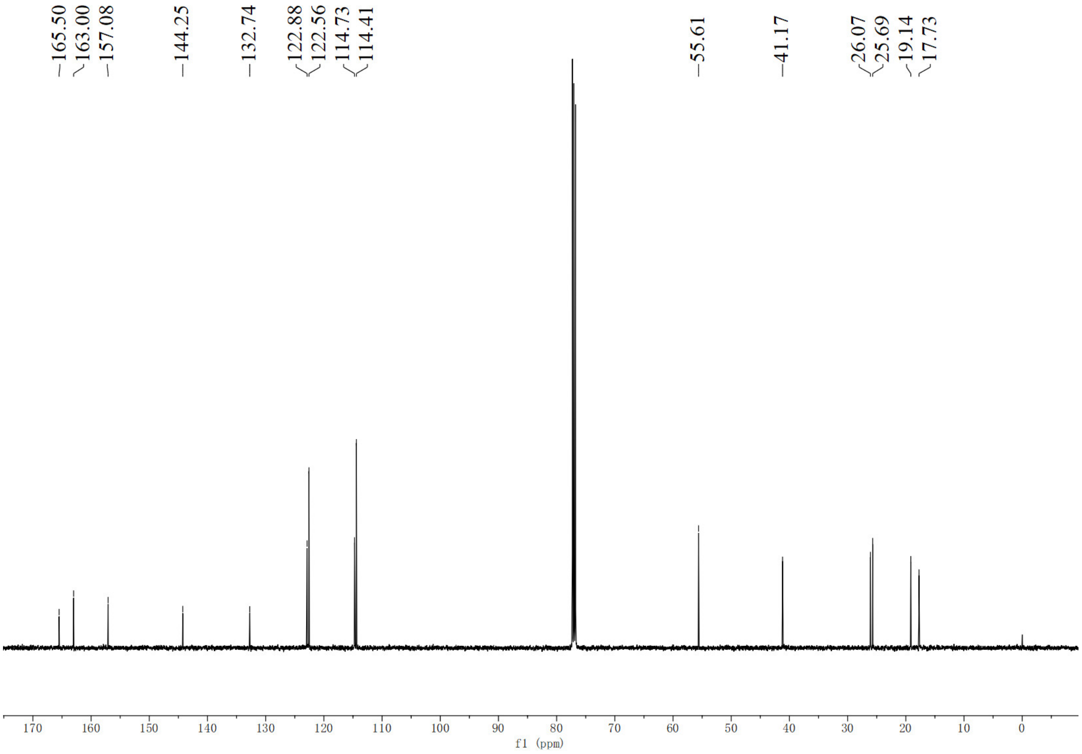

^{1}H NMR of **5h**

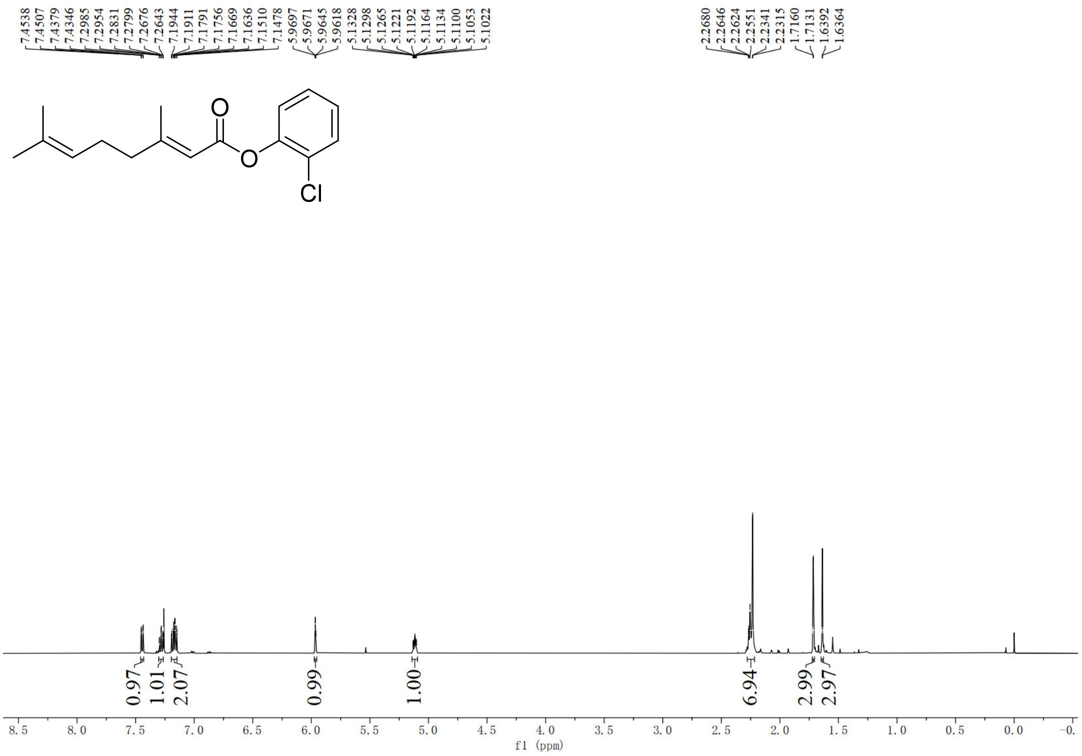

^{13}C NMR of **5h**

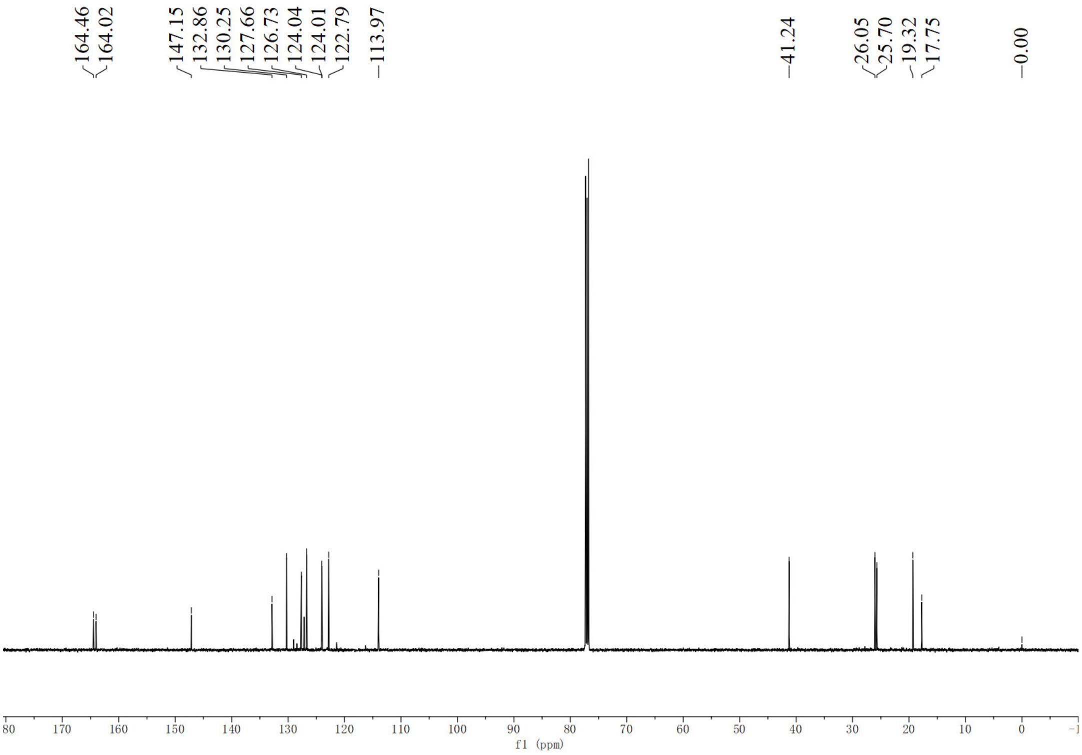

^{1}H NMR of **5i**

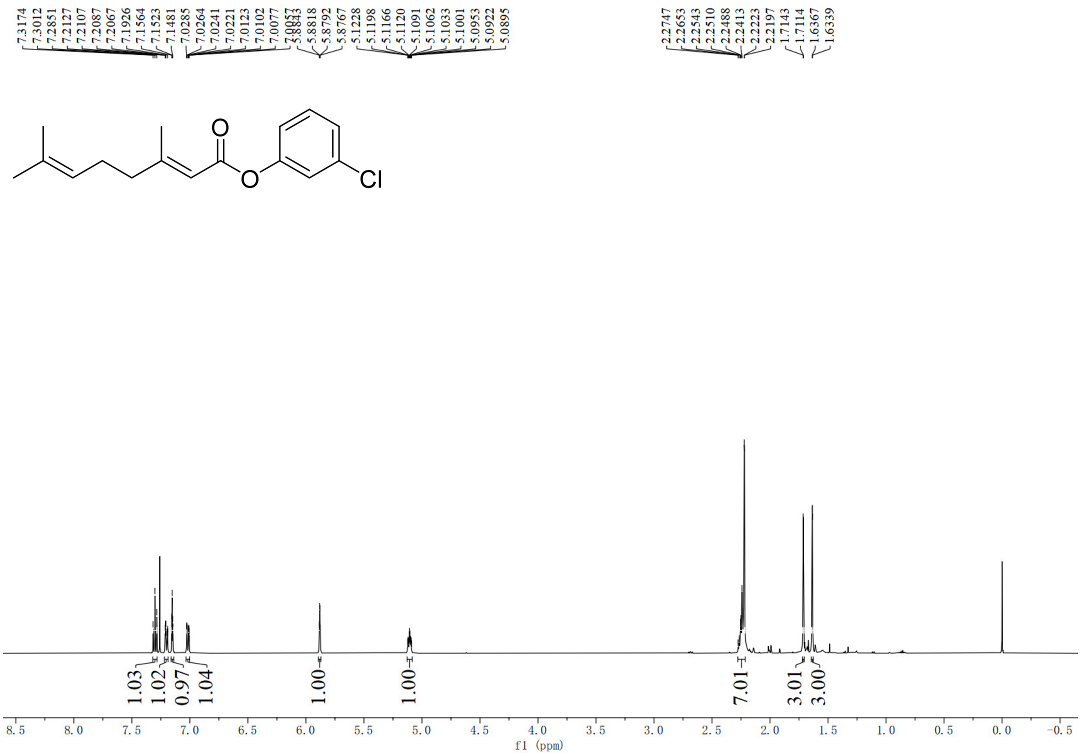

^{13}C NMR of **5i**

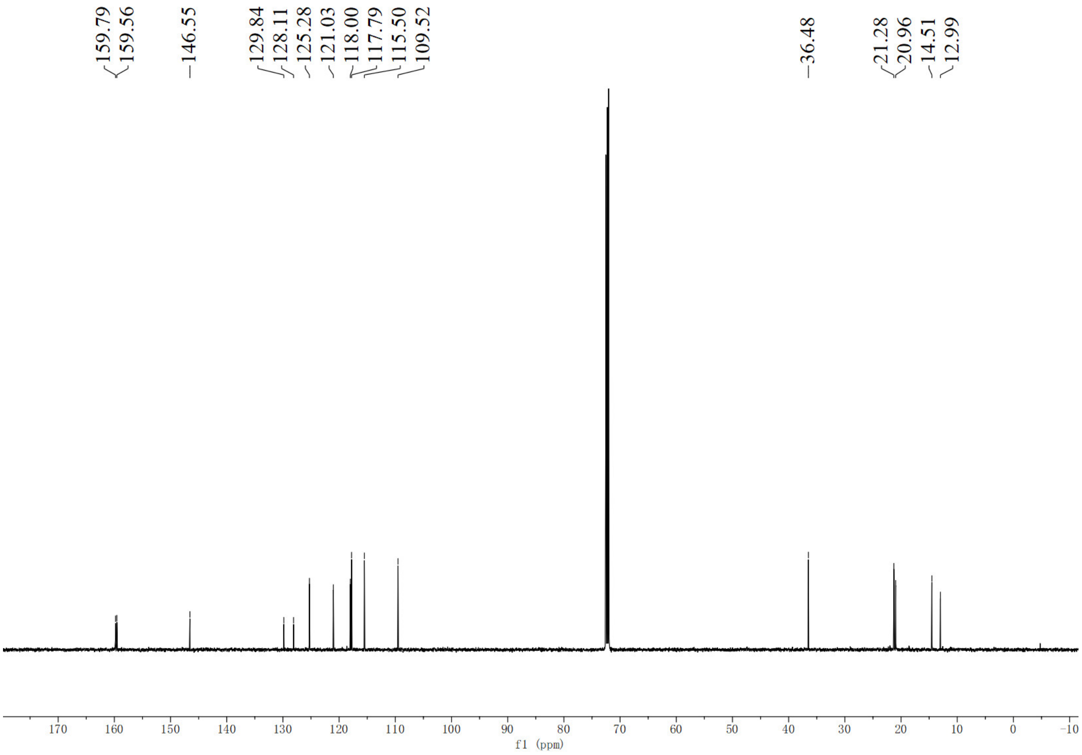

^{1}H NMR of **5j**

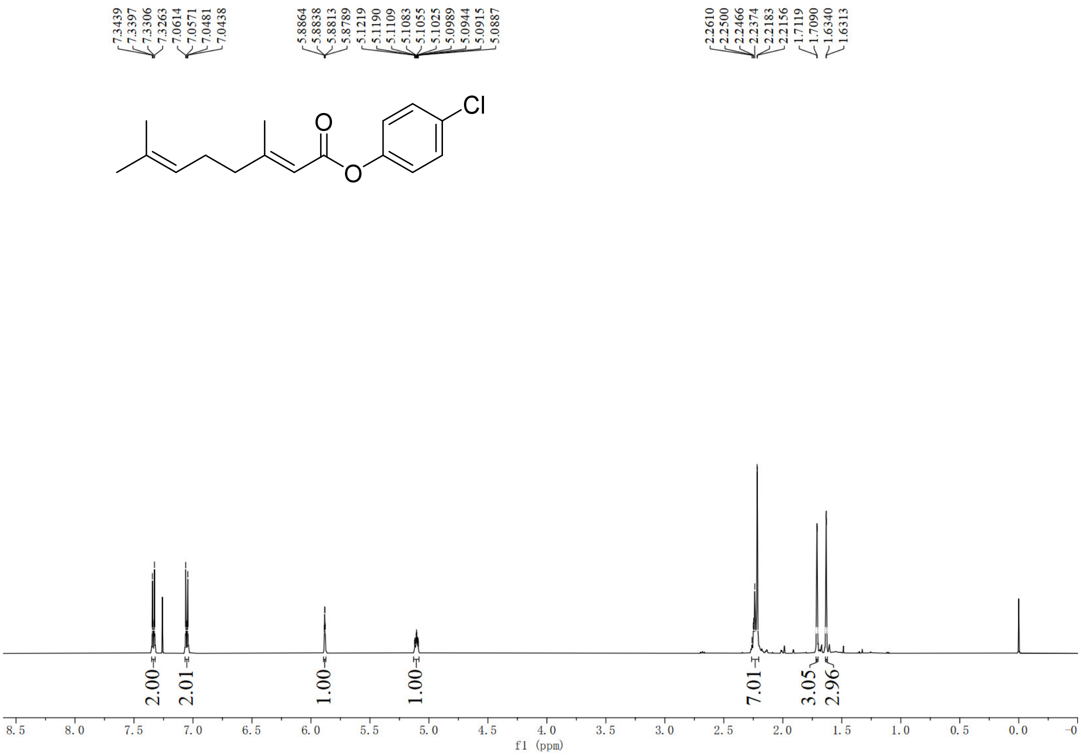

^{13}C NMR of **5j**

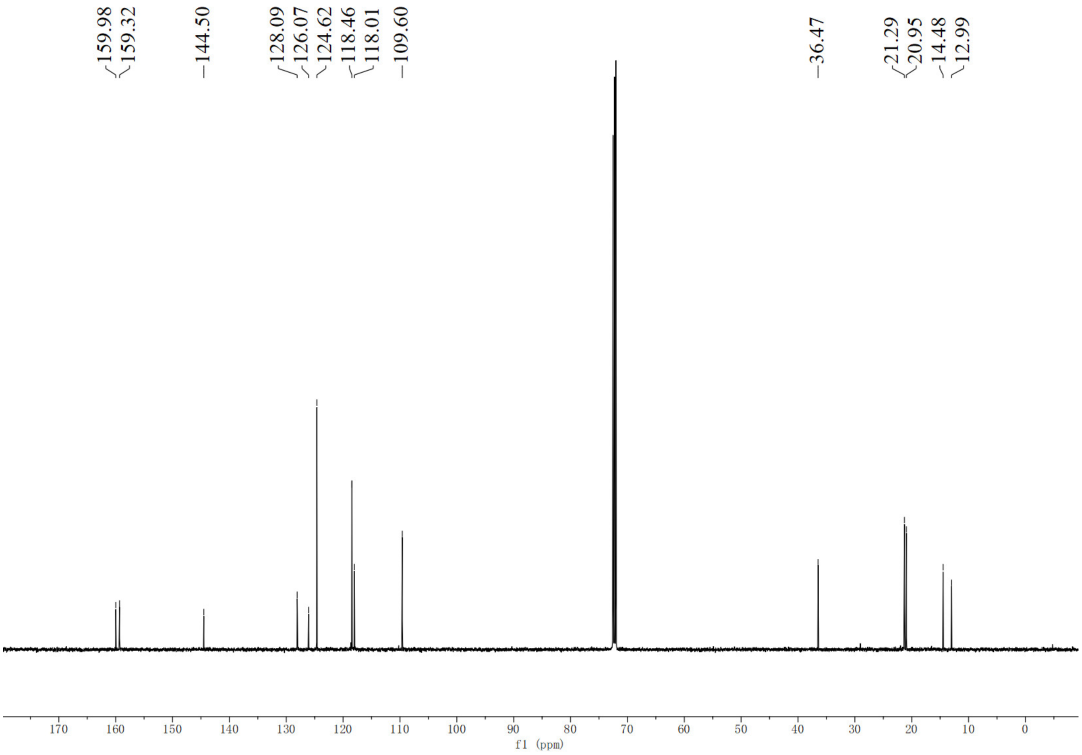

^{1}H NMR of **5k**

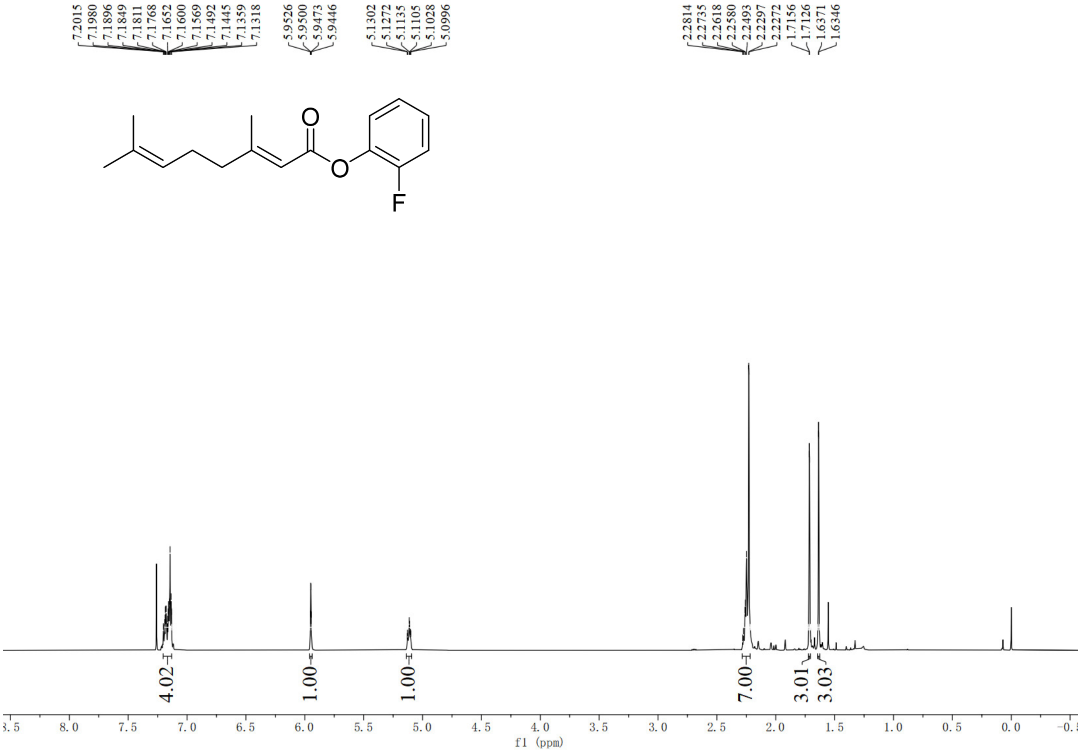

^{13}C NMR of **5k**

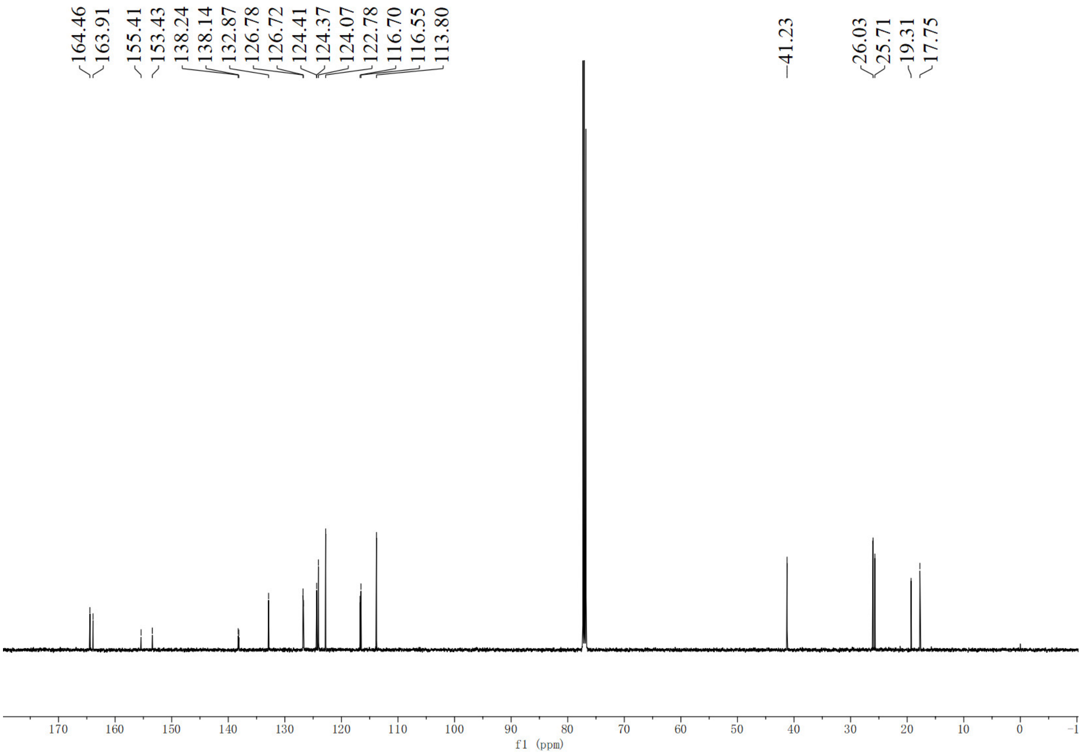

^{1}H NMR of **5l**

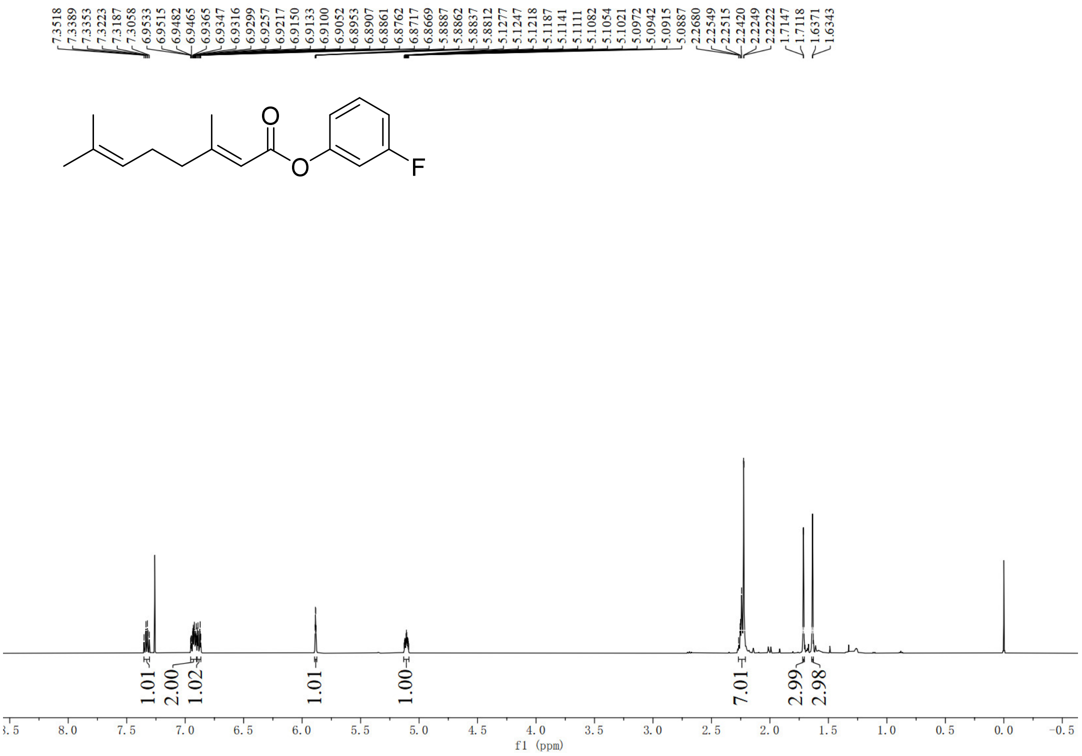

^{13}C NMR of **5l**

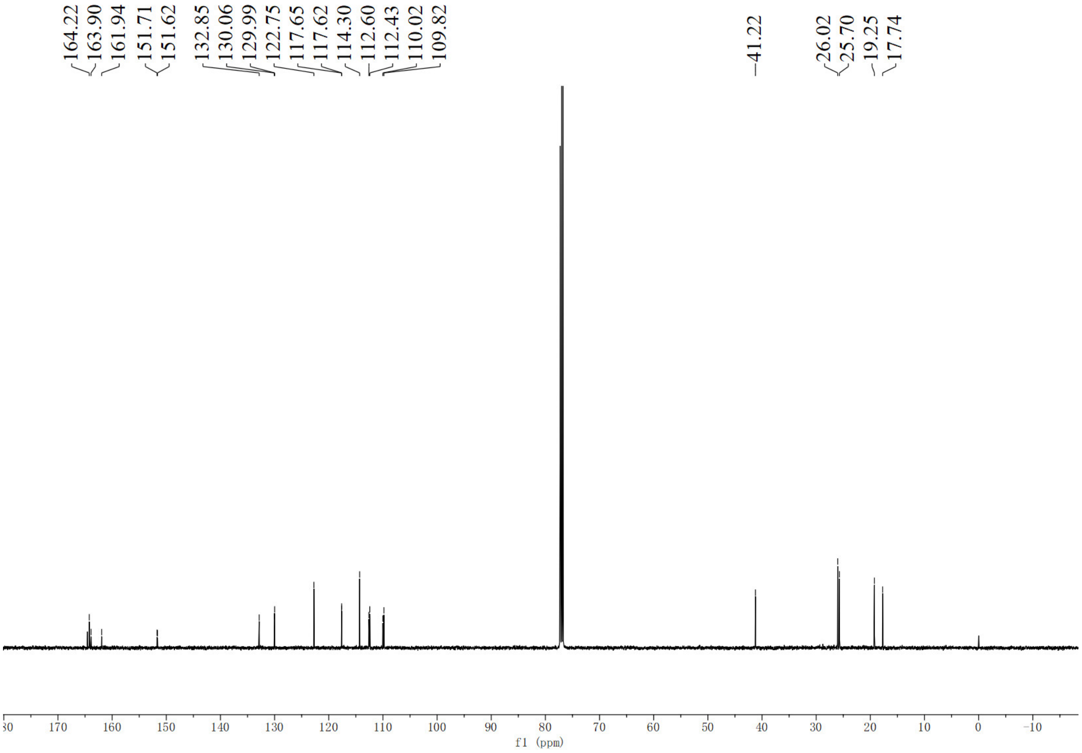

^{1}H NMR of **5m**

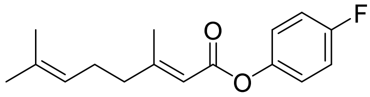

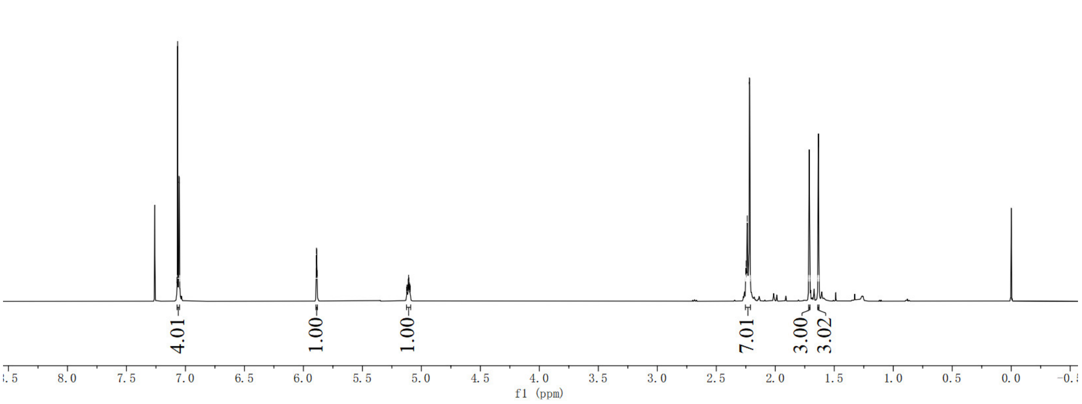

^{13}C NMR of **5m**

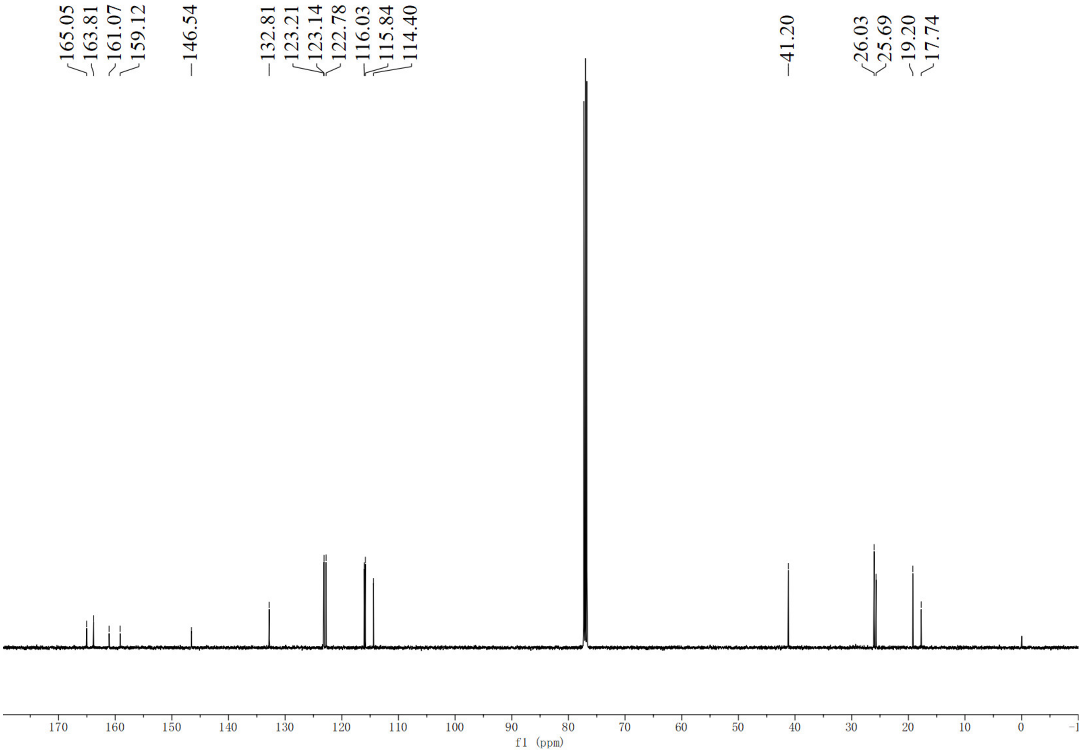

^{1}H NMR of **5n**

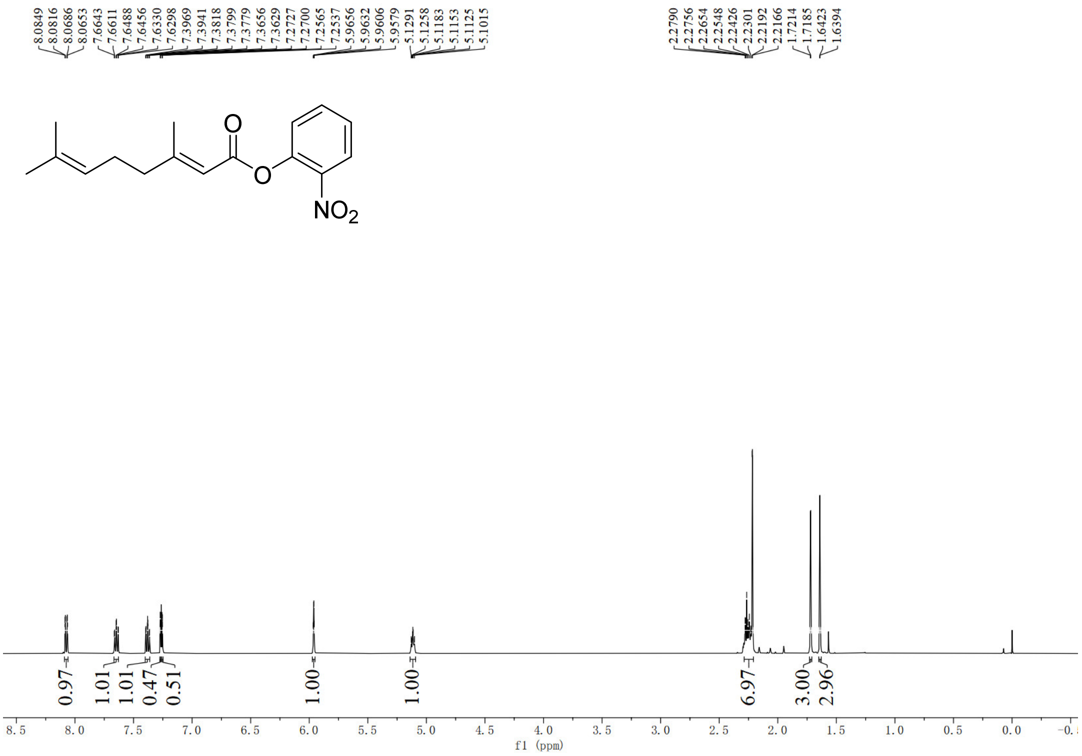

^{13}C NMR of **5n**

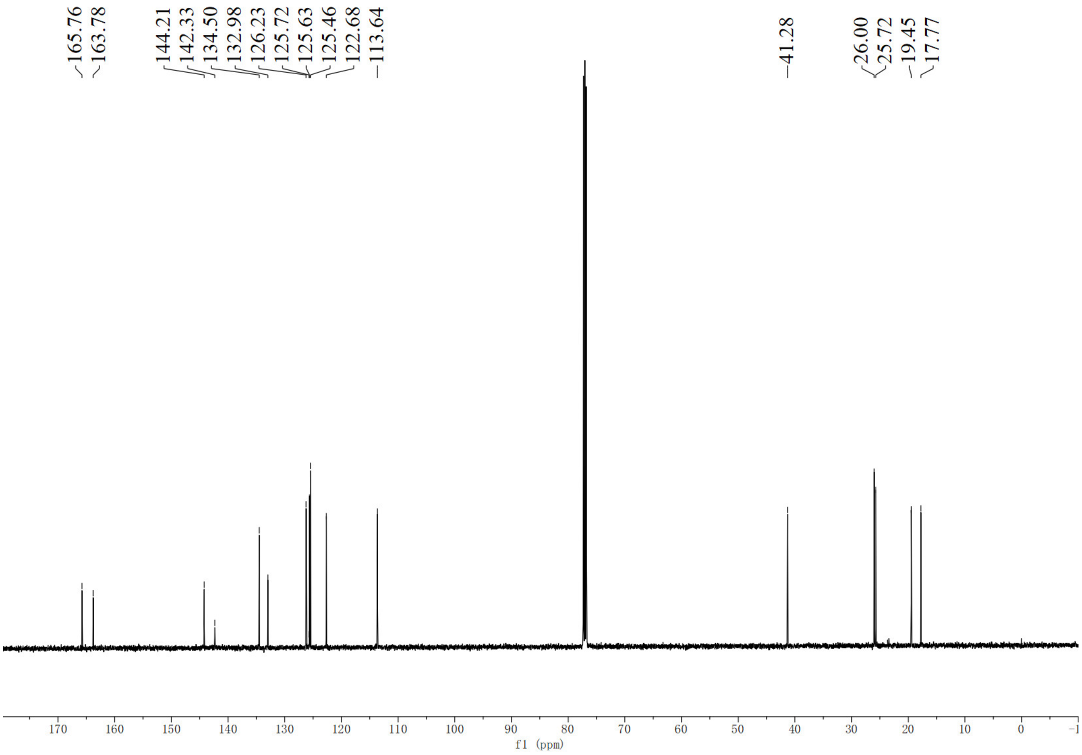

^{1}H NMR of **5o**

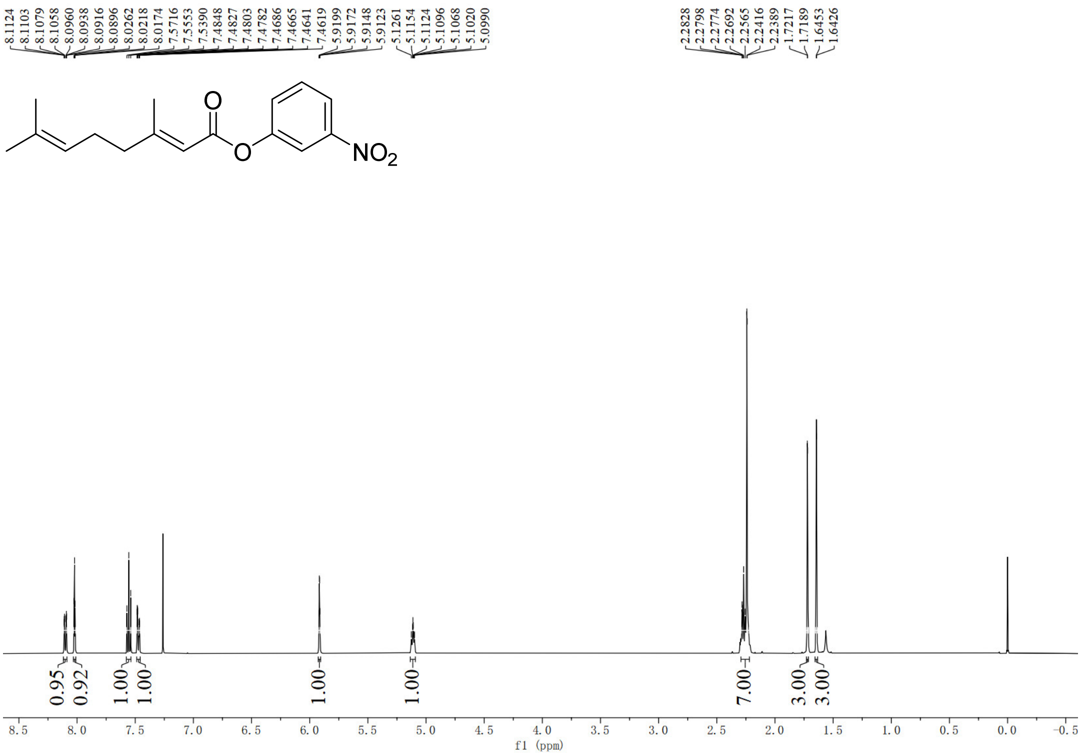

^{13}C NMR of **5o**

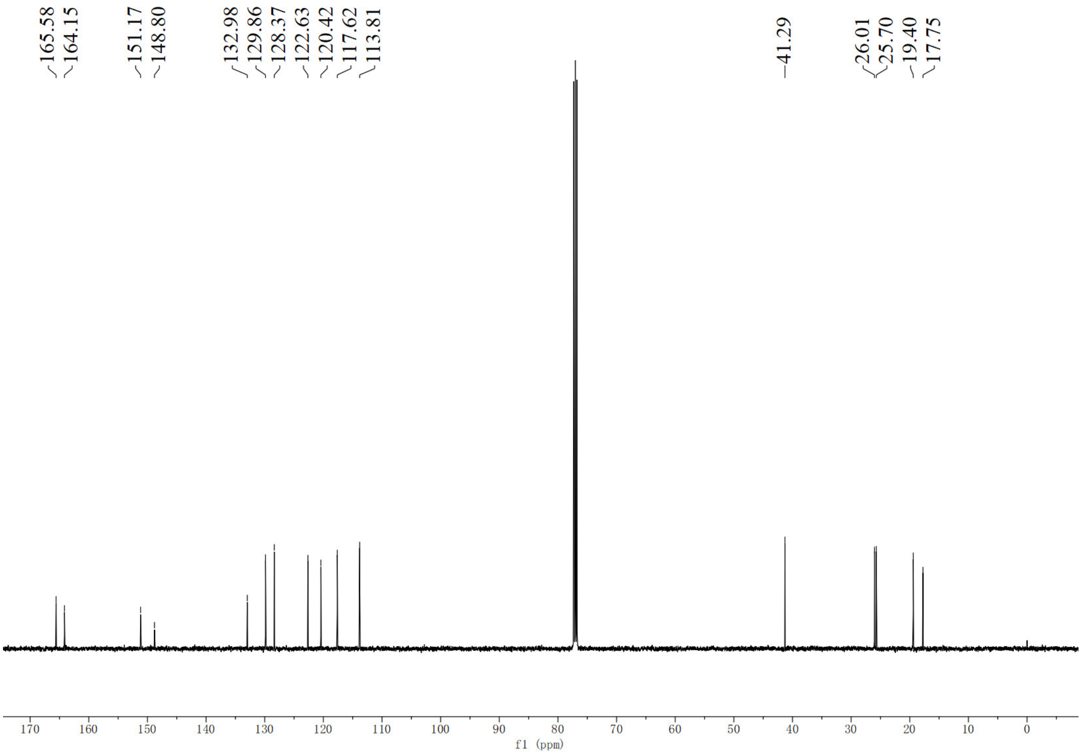

^{1}H NMR of **5p**

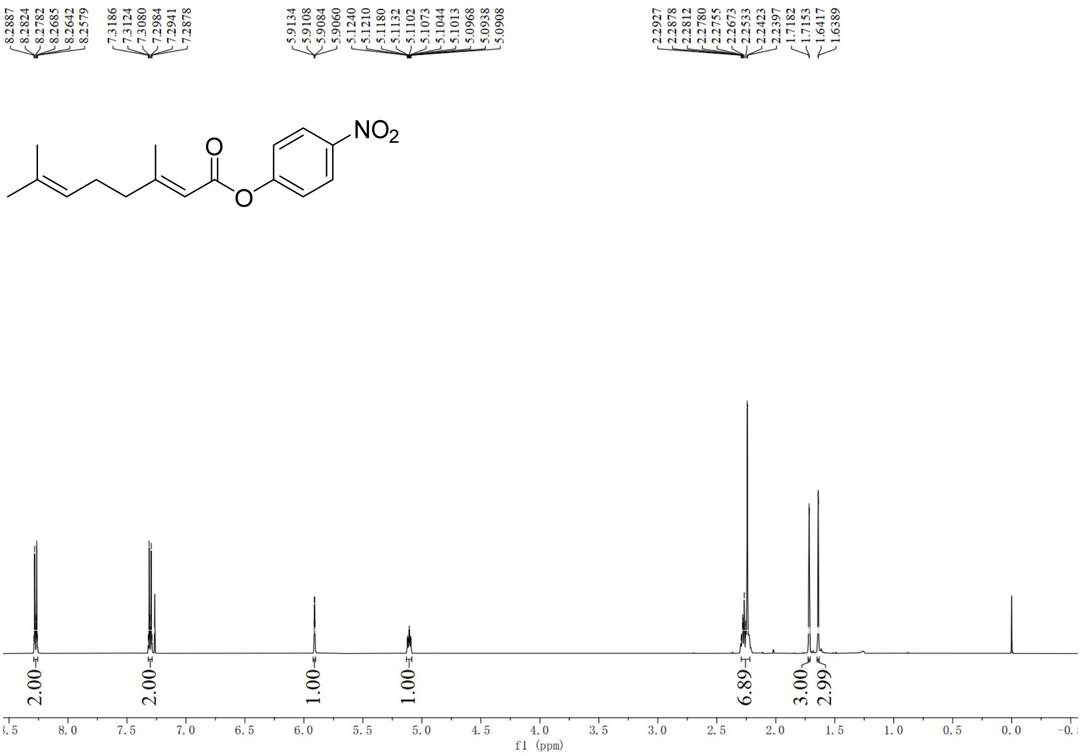

^{13}C NMR of **5p**

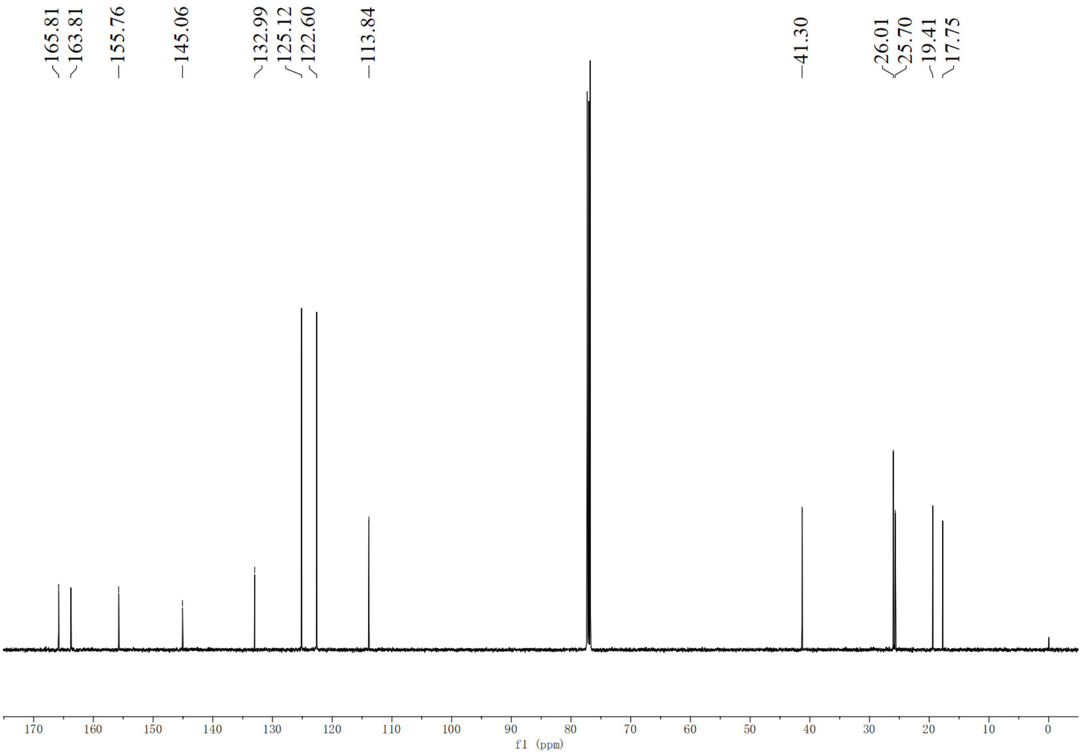

^{1}H NMR of **5q**

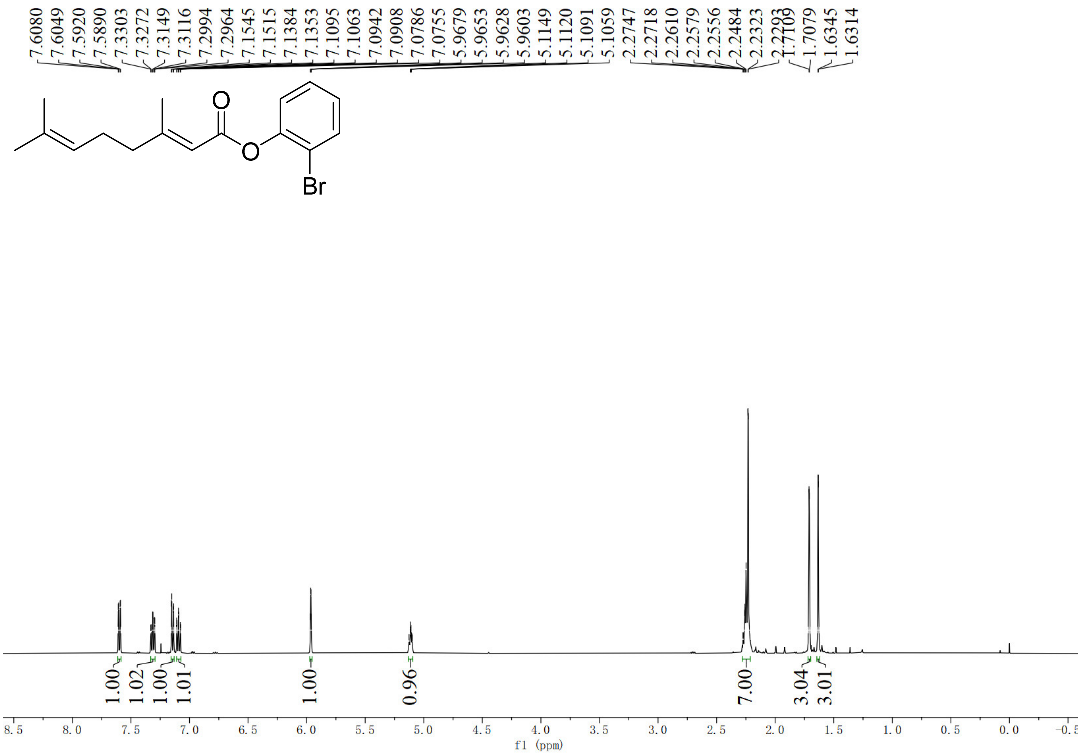

^{13}C NMR of **5q**

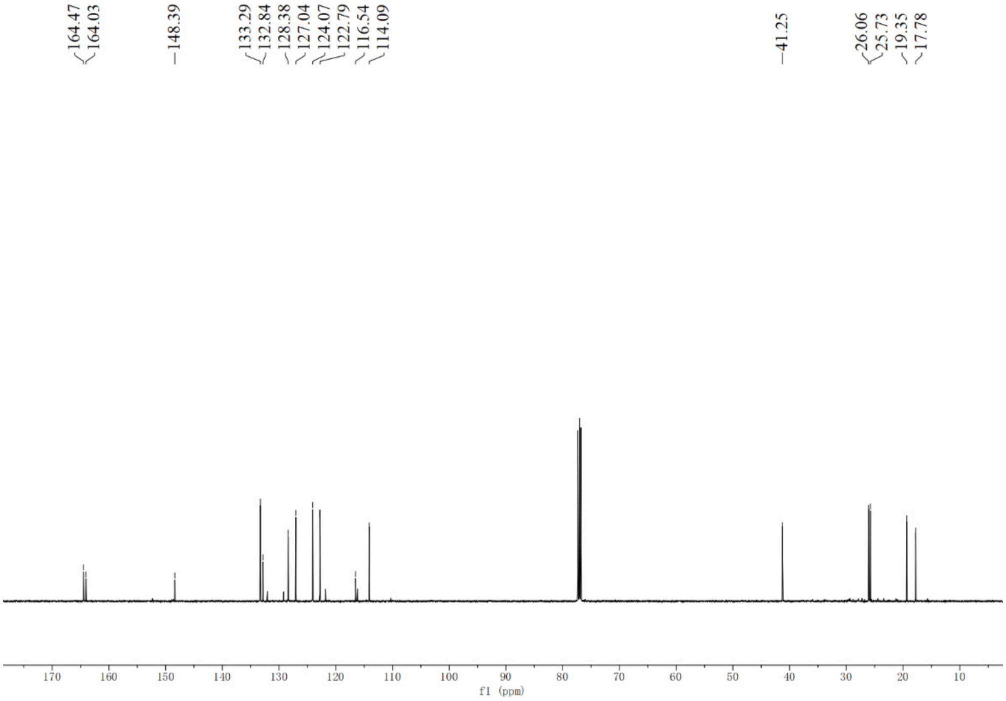

^{1}H NMR of **5r**

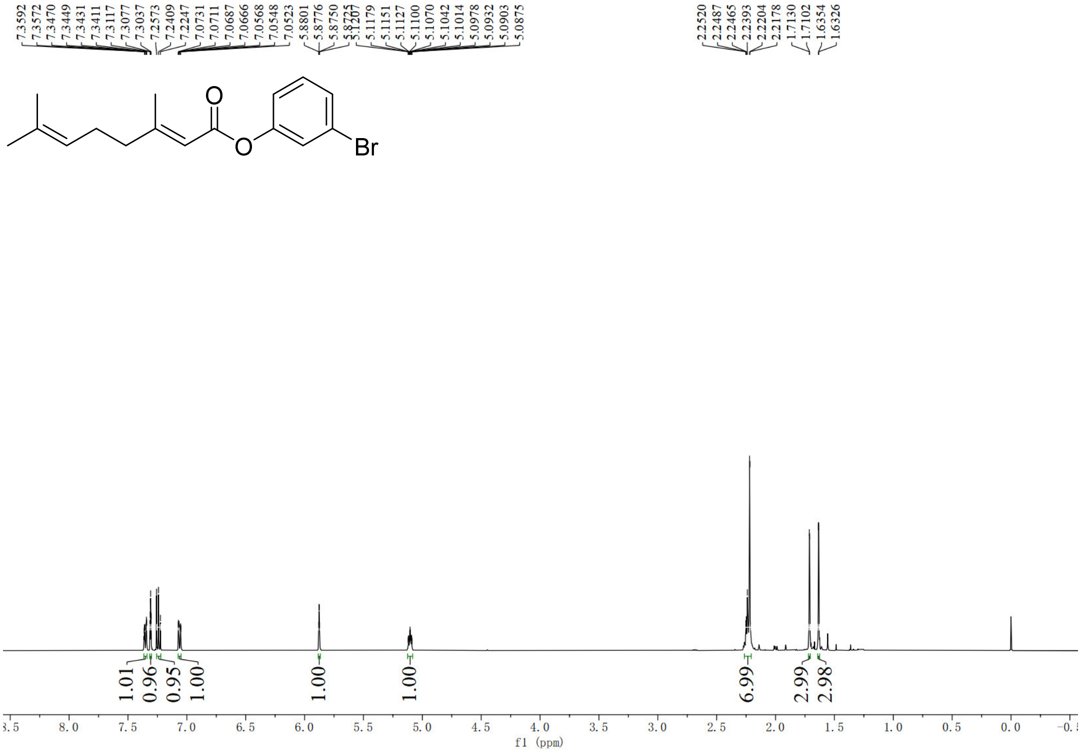

^{13}C NMR of **5r**

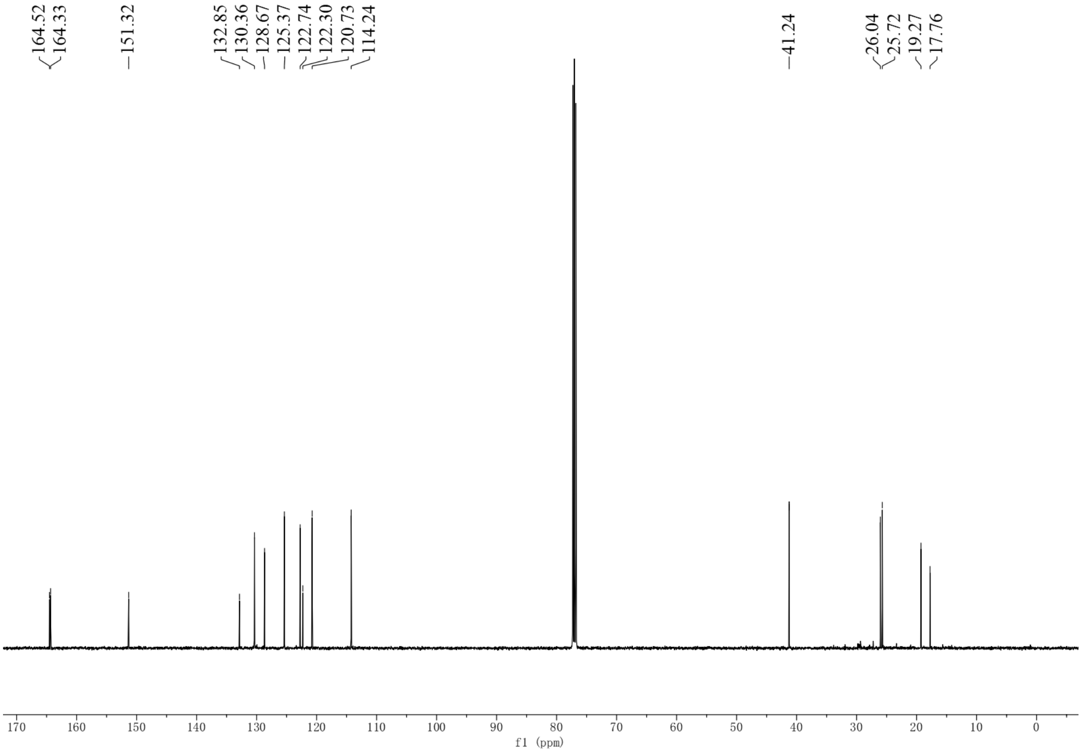

^{1}H NMR of **5s**

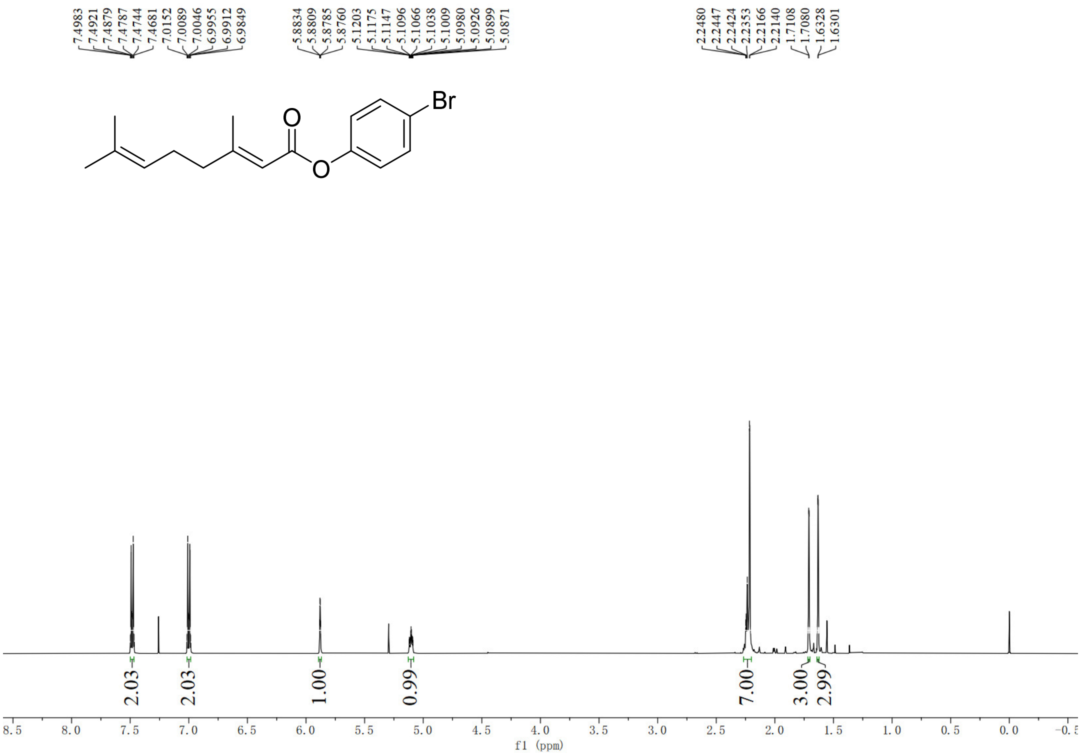

^{13}C NMR of **5s**

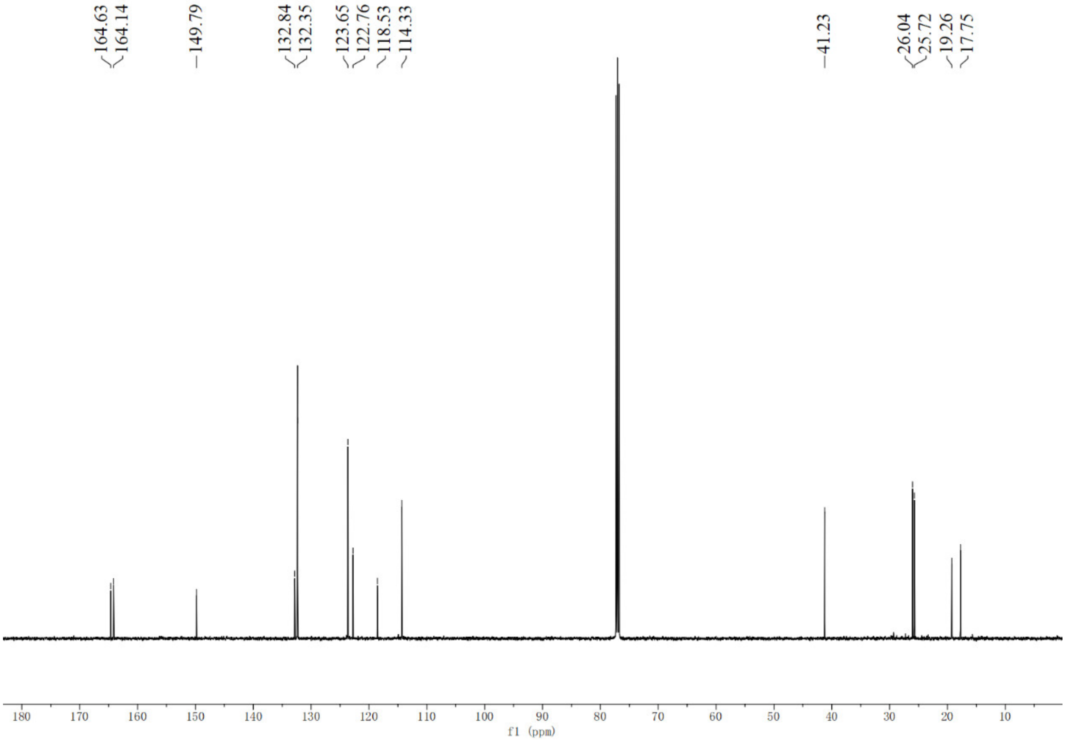

^{1}H NMR of **5t**

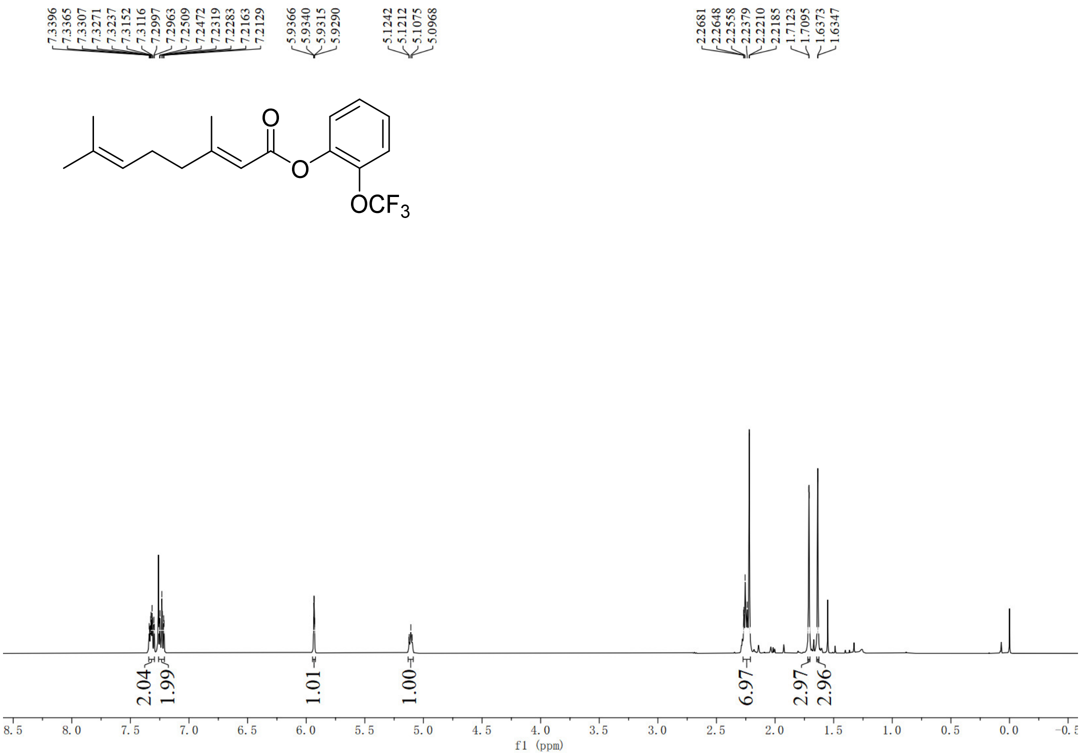

^{13}C NMR of **5t**

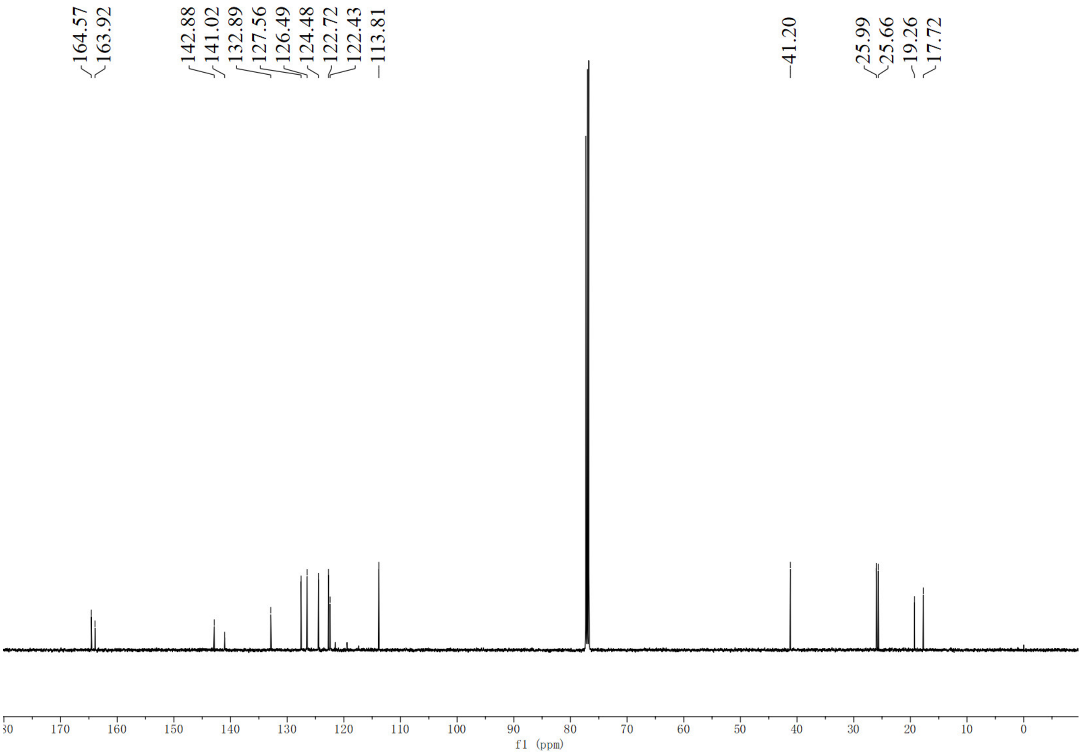

^{1}H NMR of **5u**

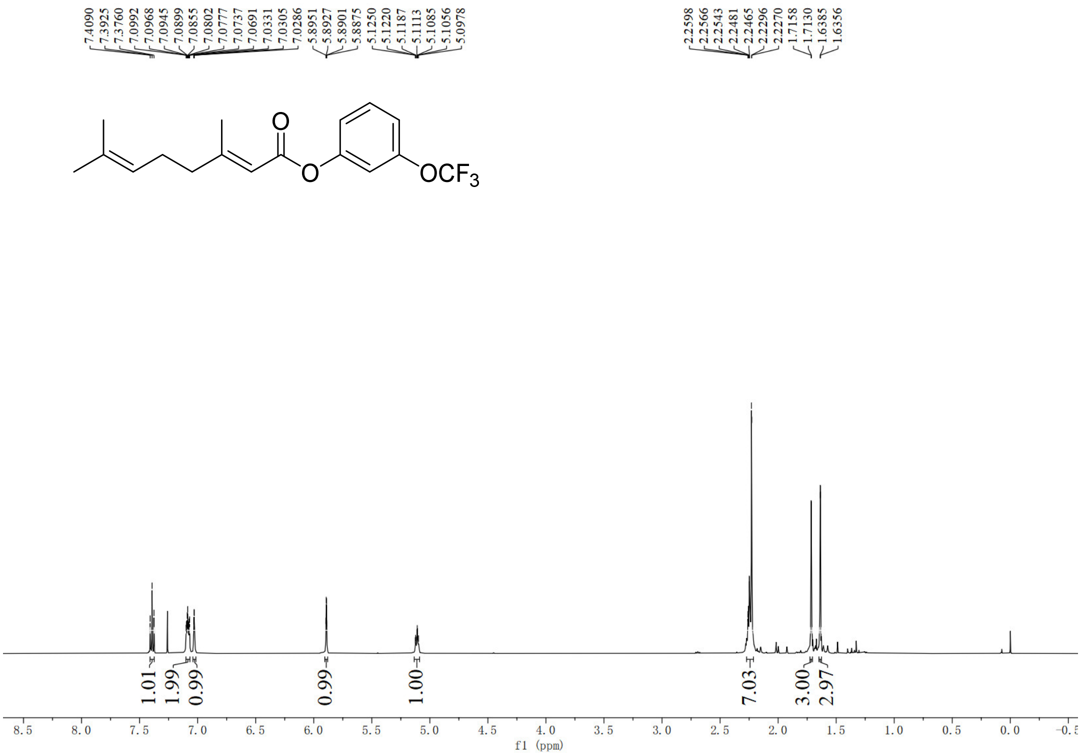

^{13}C NMR of **5u**

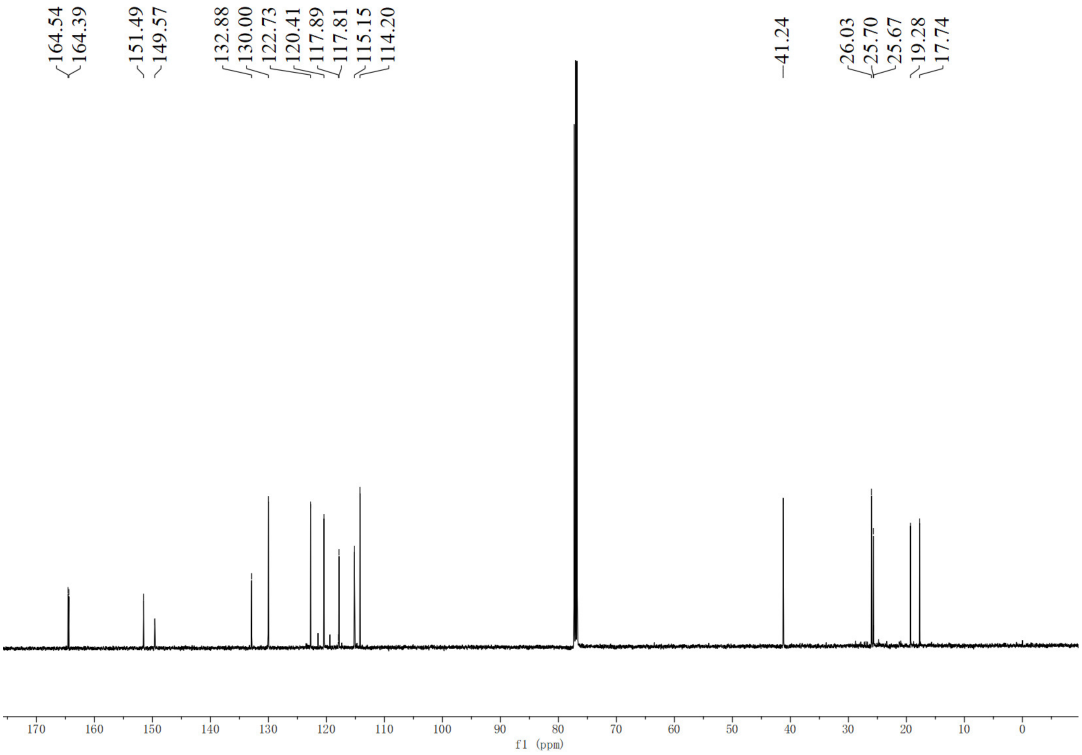

^{1}H NMR of **5v**

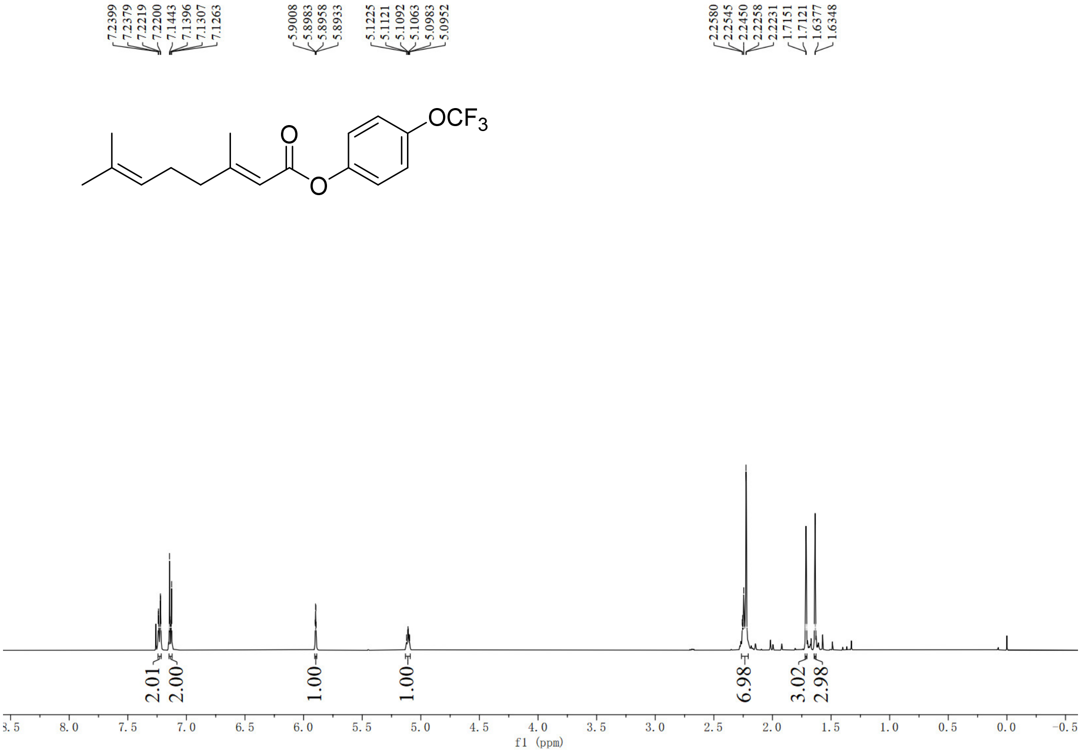

^{13}C NMR of **5v**

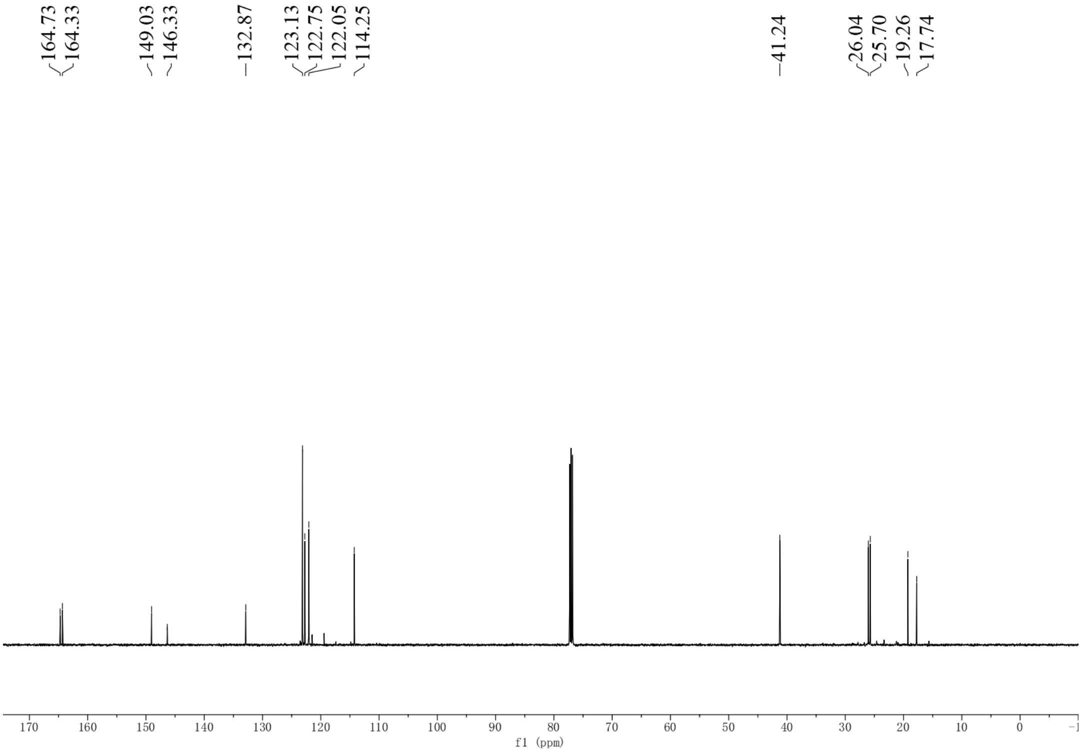

^{1}H NMR of **5w**

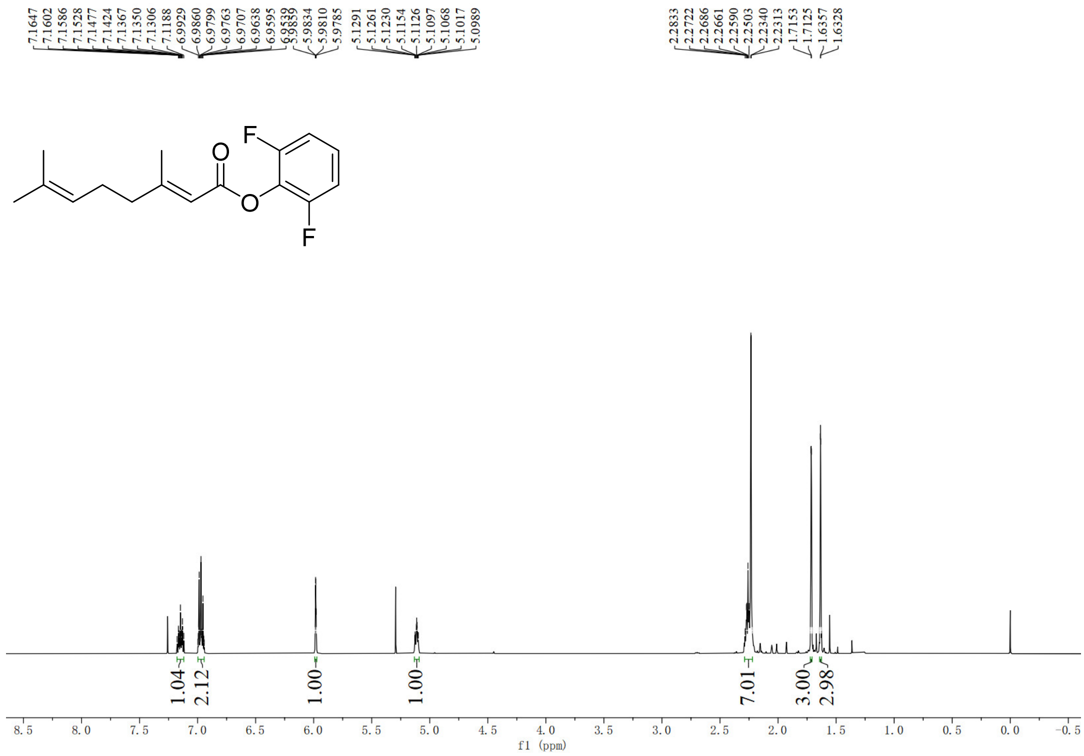

^{13}C NMR of **5w**

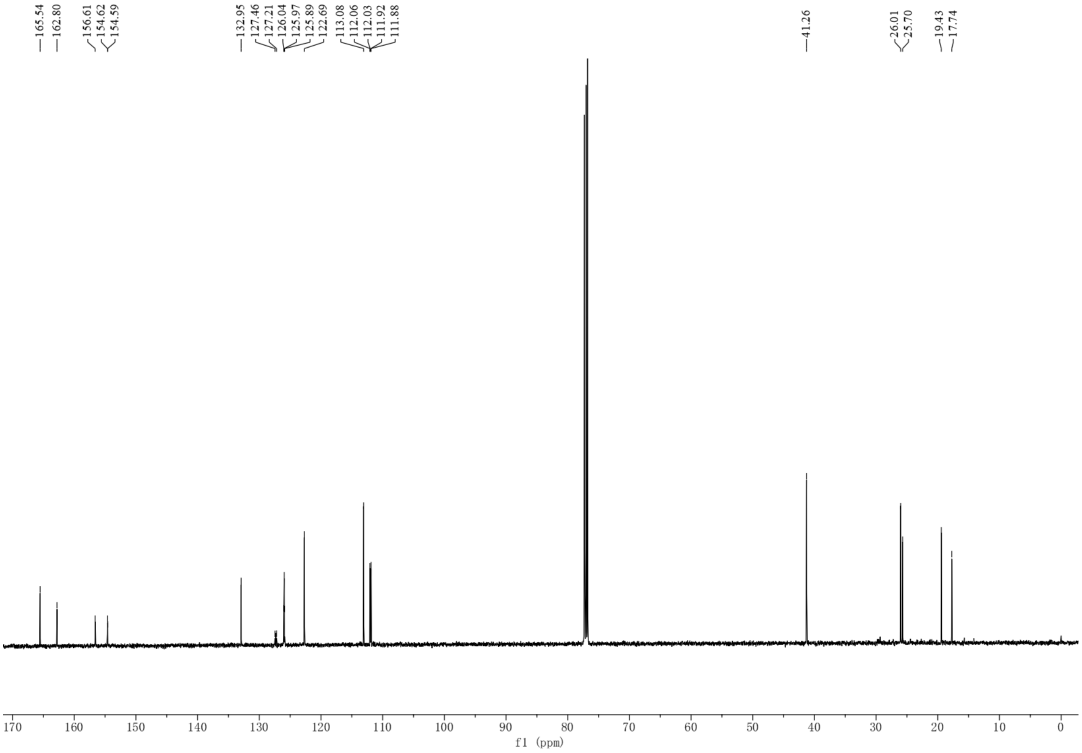

^{1}H NMR of **5x**

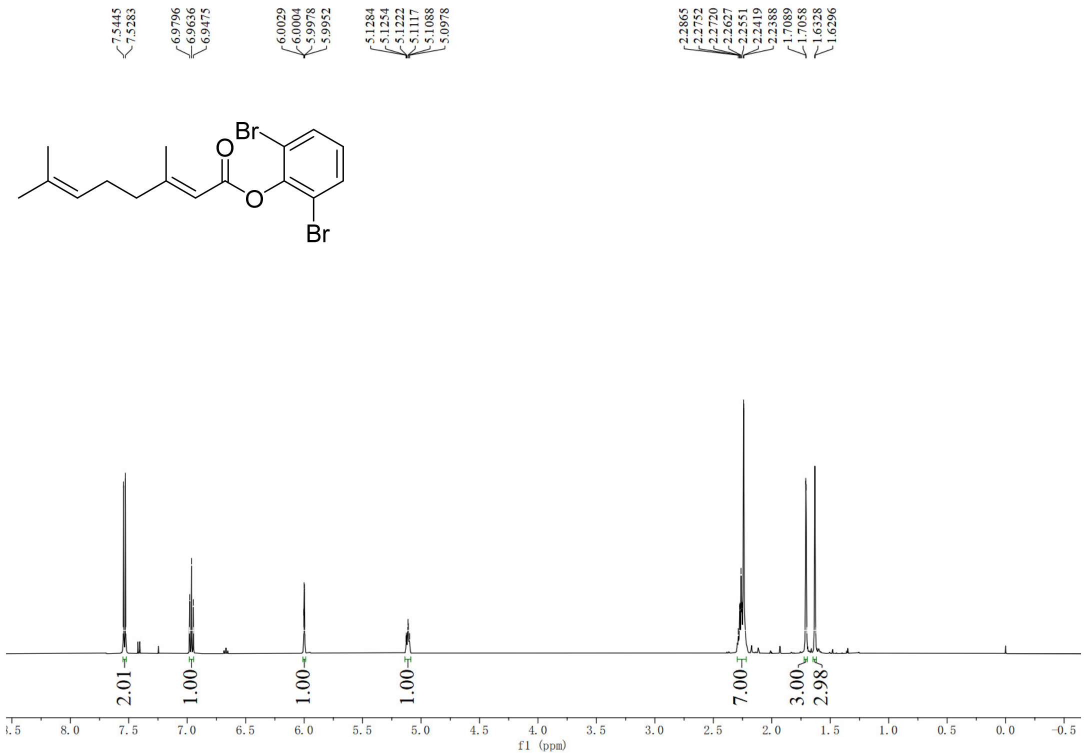

^{13}C NMR of **5x**

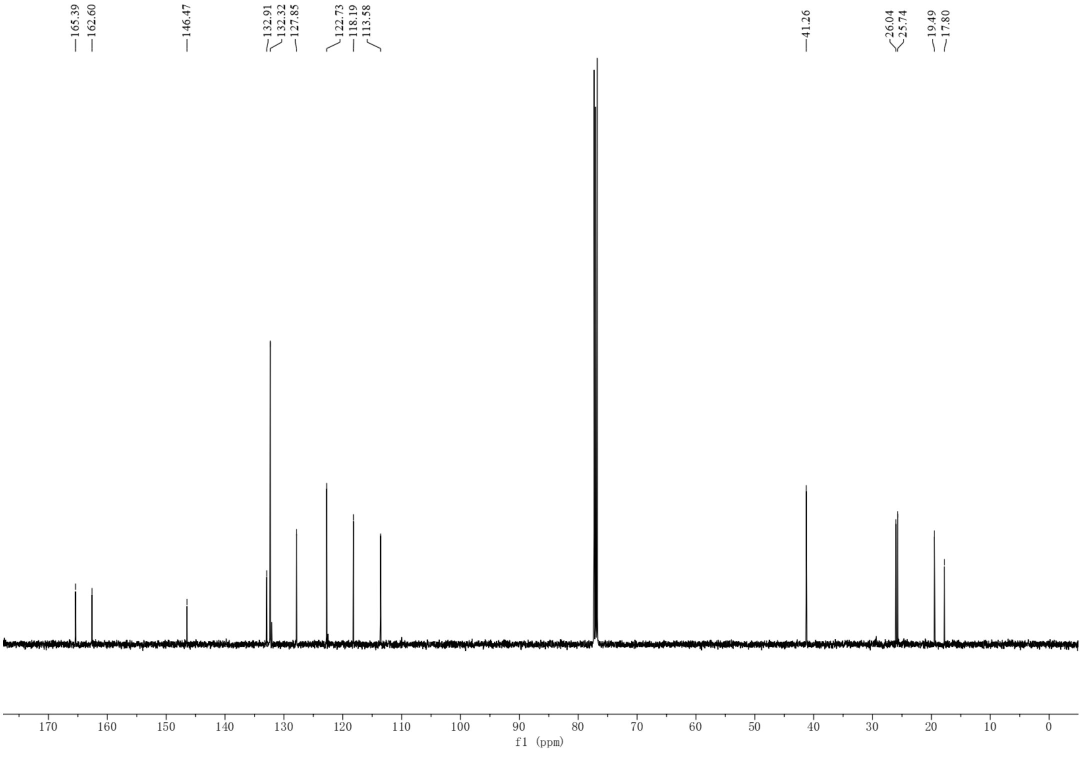

5. HRMS Spectrums of compounds **5a-5x**

HRMS Spectrum of **5a**

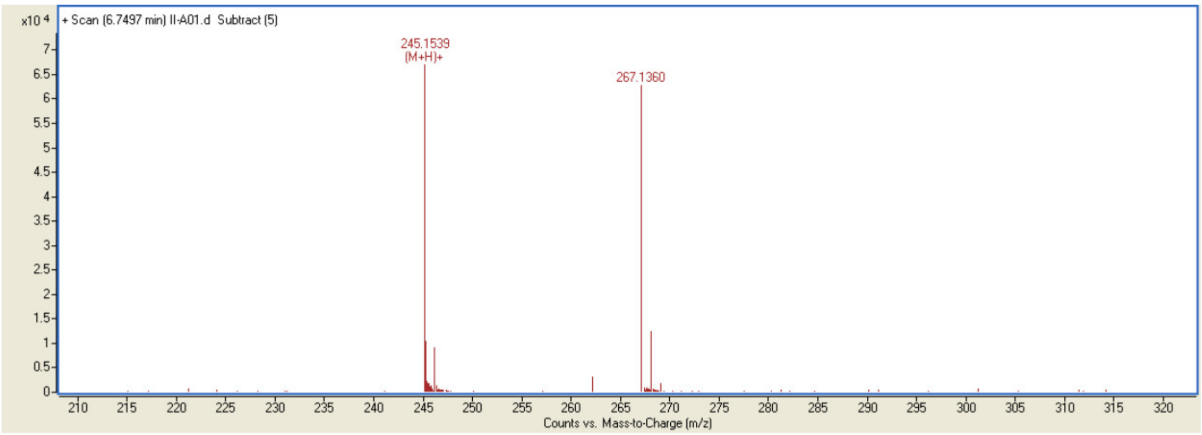

HRMS Spectrum of **5b**

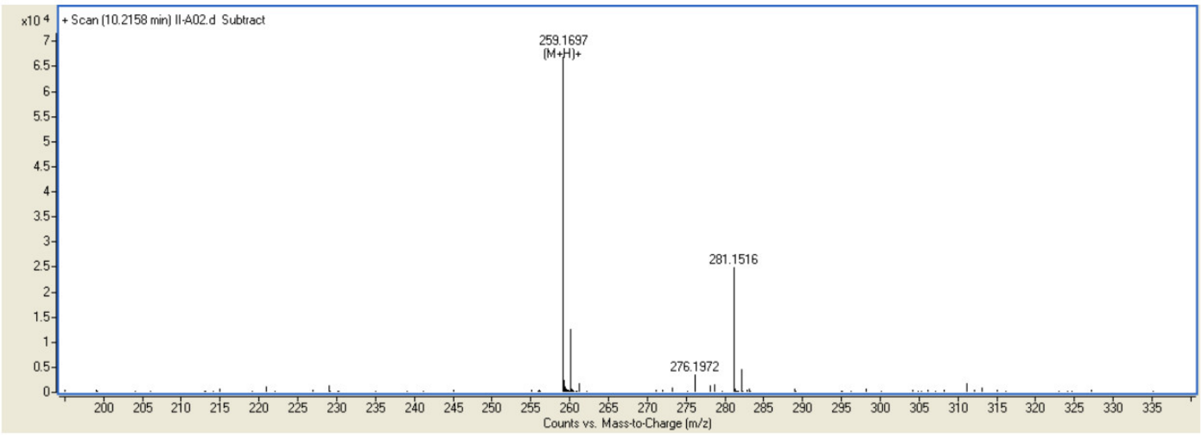

HRMS Spectrum of **5c**

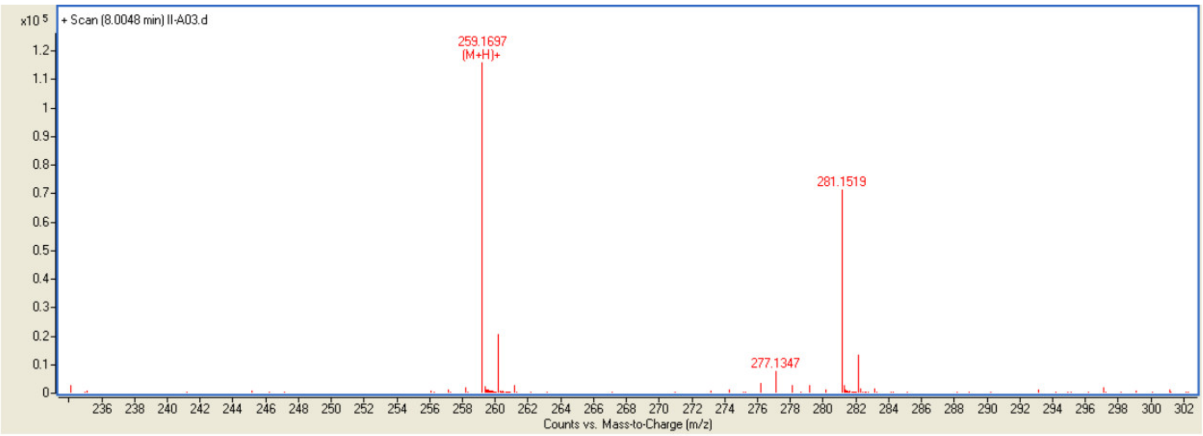

HRMS Spectrum of **5d**

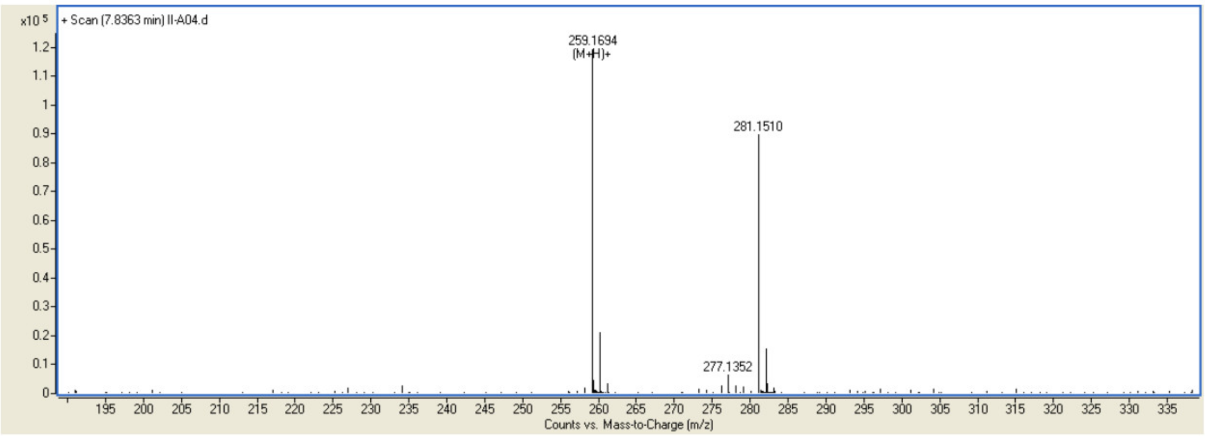

HRMS Spectrum of **5e**

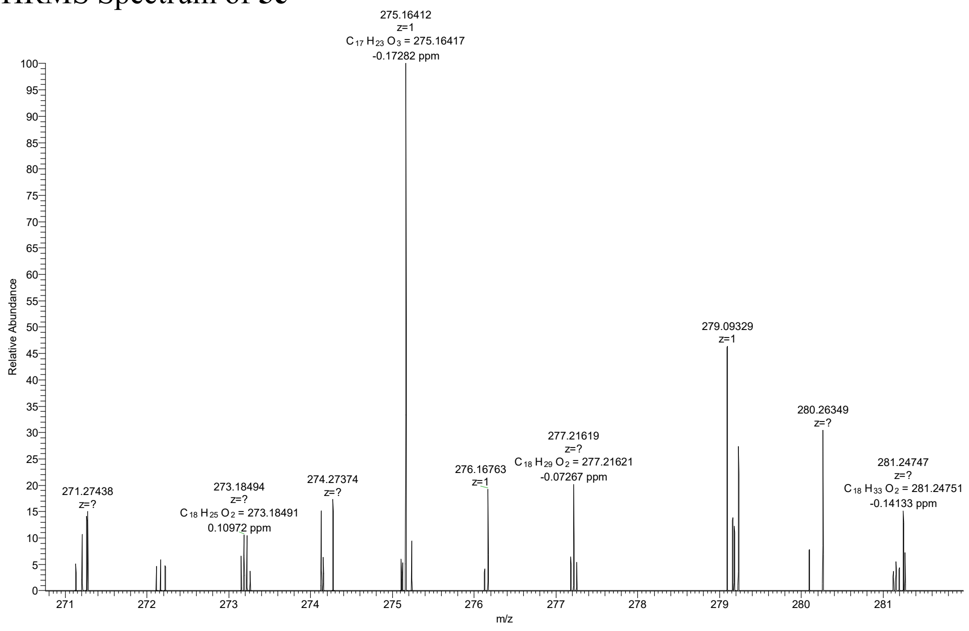

HRMS Spectrum of **5f**

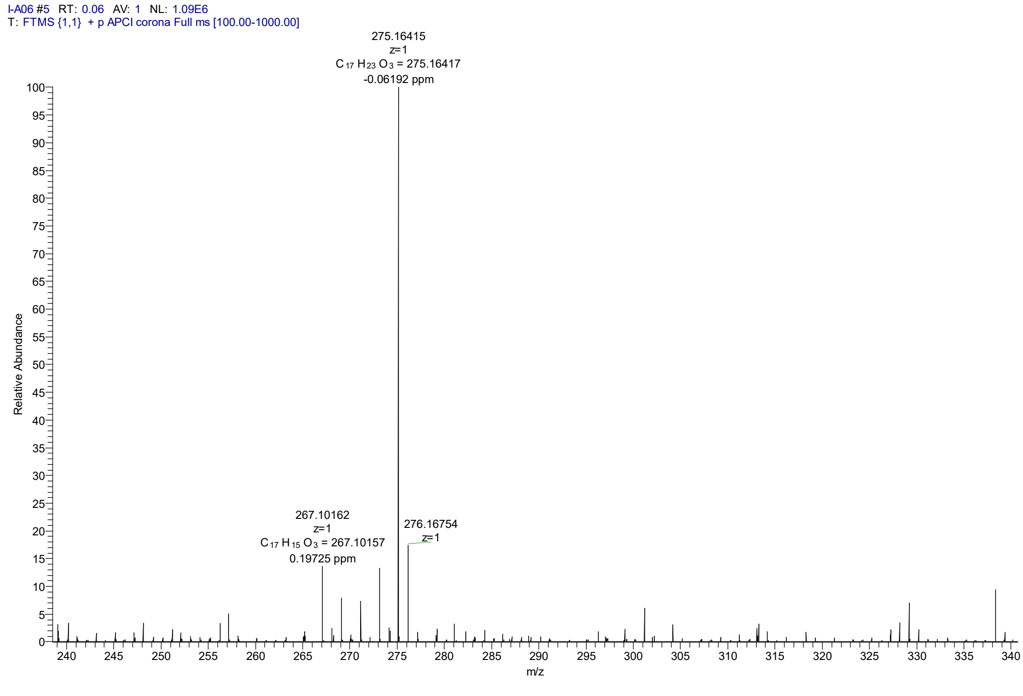

HRMS Spectrum of **5g**

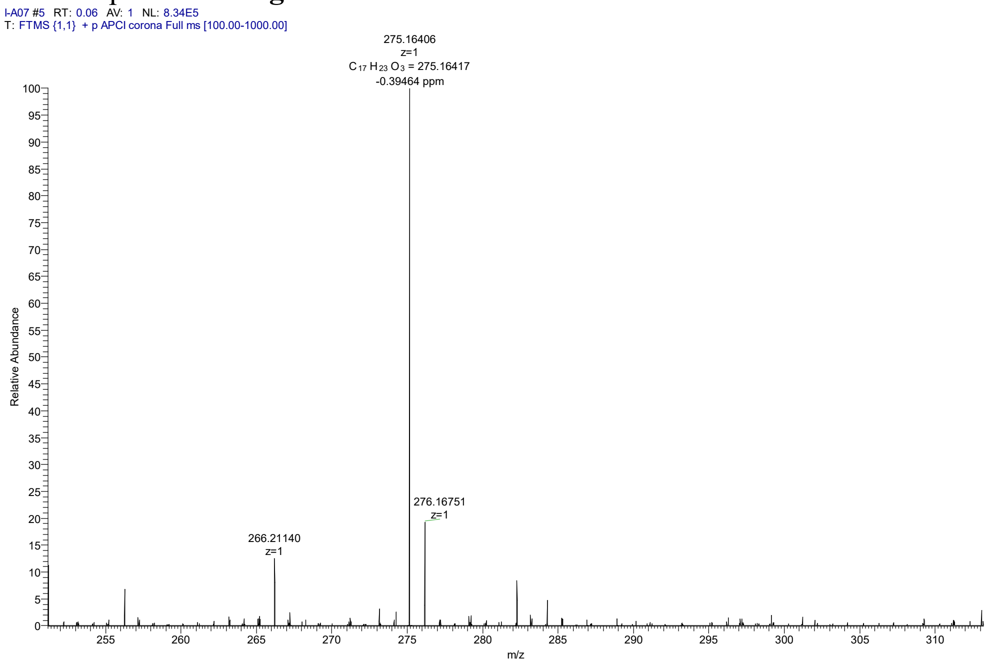

HRMS Spectrum of **5h**

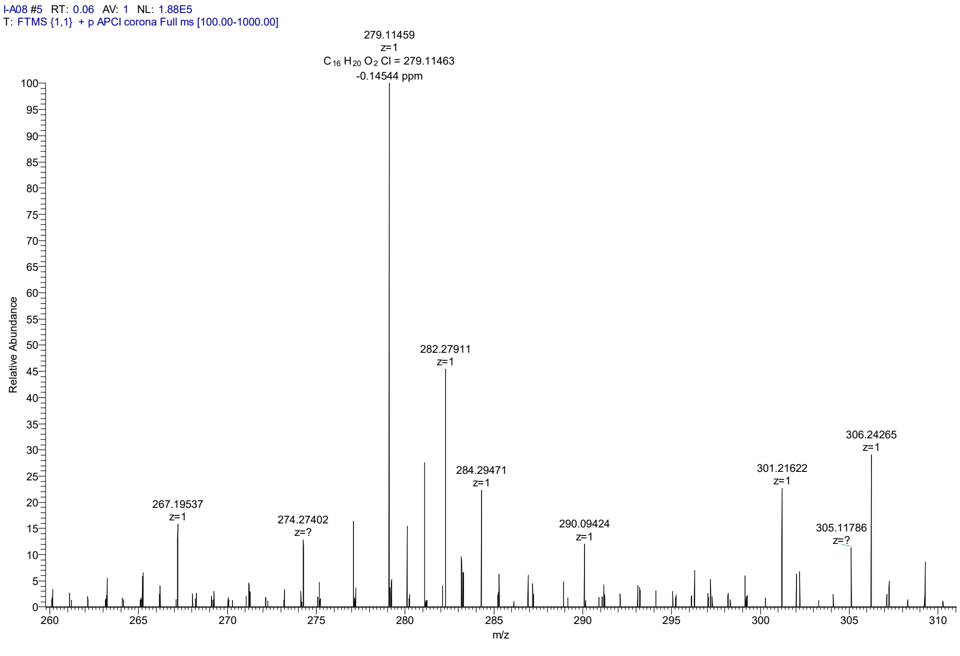

HRMS Spectrum of **5i**

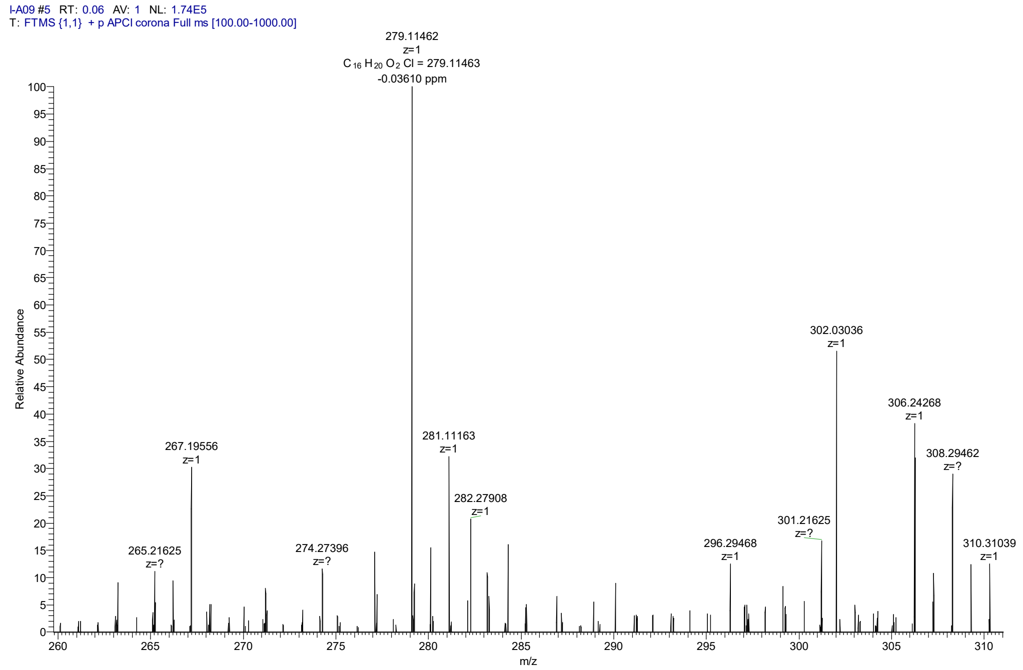

HRMS Spectrum of **5j**

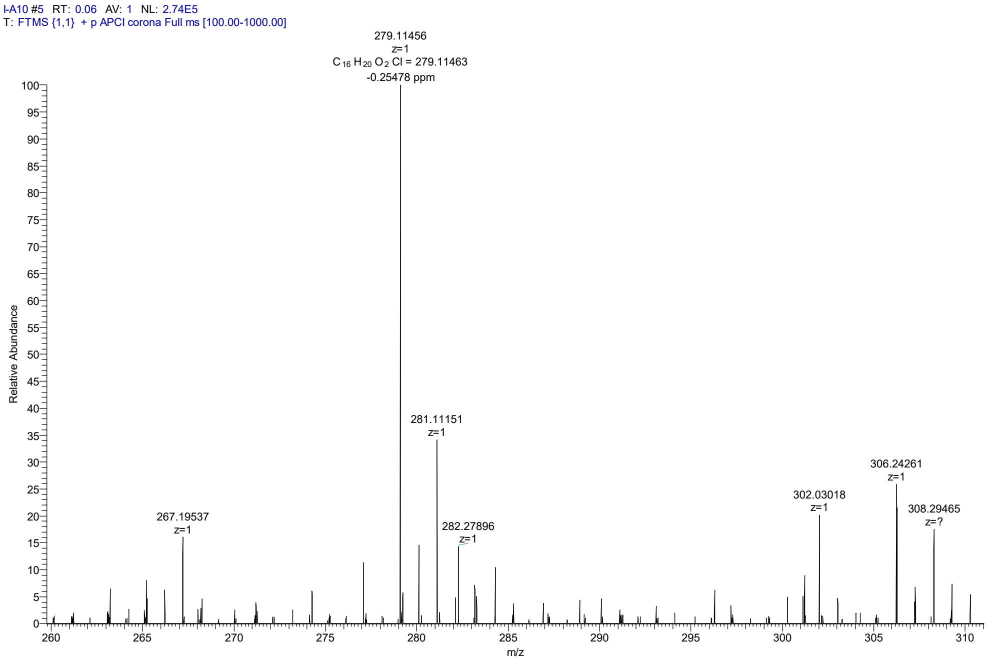

HRMS Spectrum of **5k**

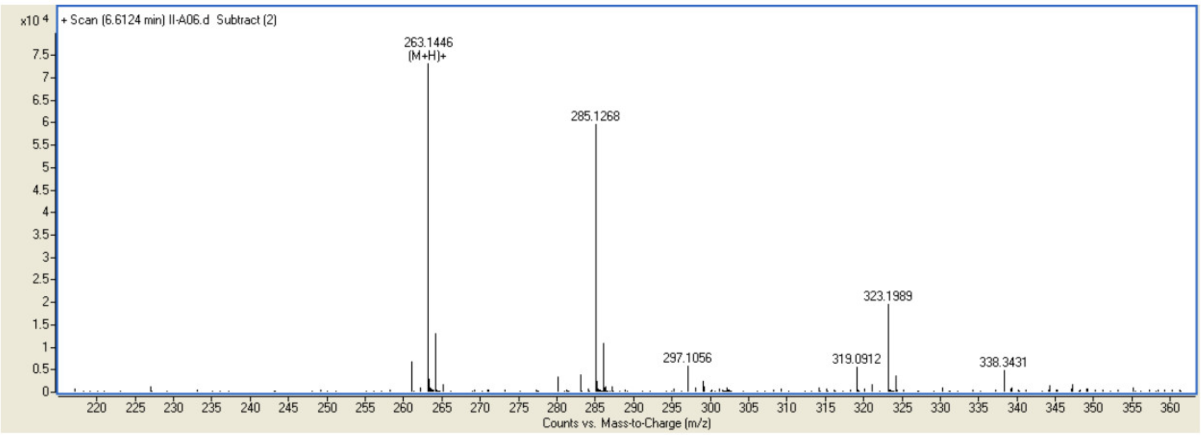

HRMS Spectrum of **5l**

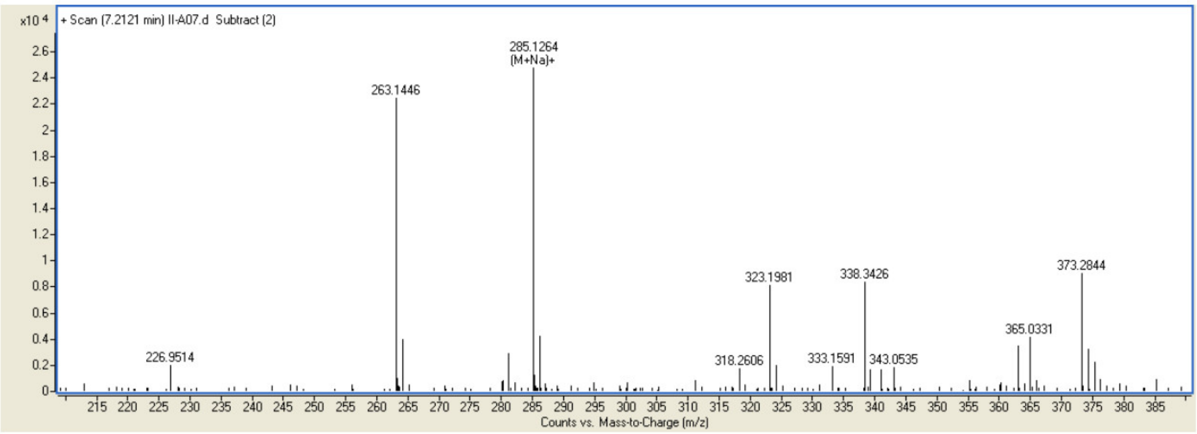

HRMS Spectrum of **5m**

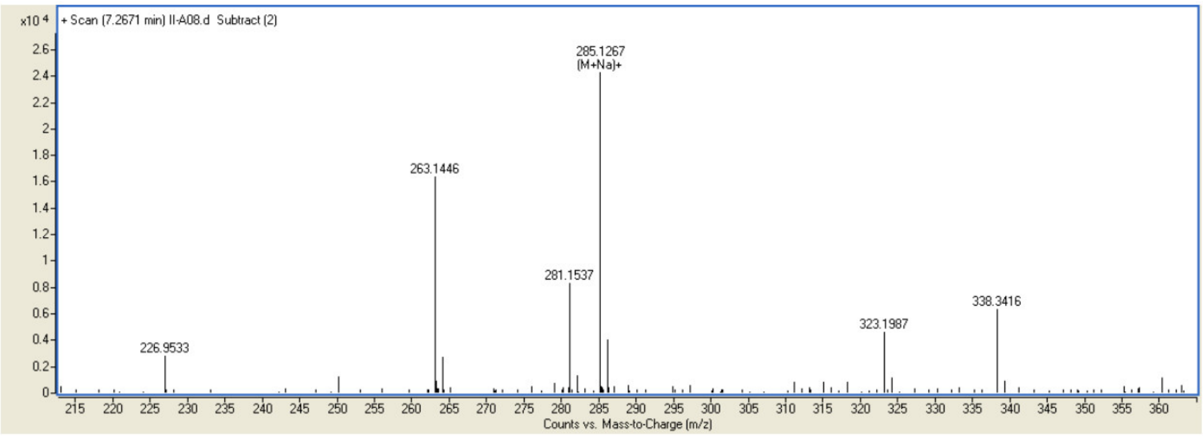

HRMS Spectrum of **5n**

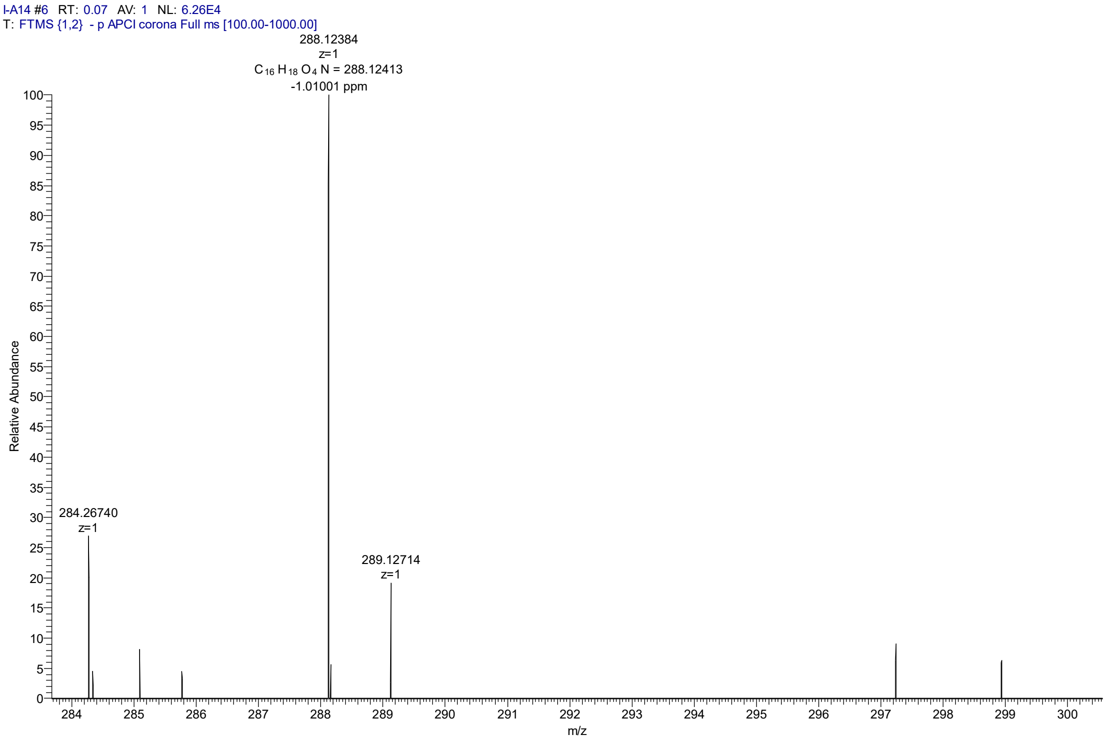

HRMS Spectrum of **5o**

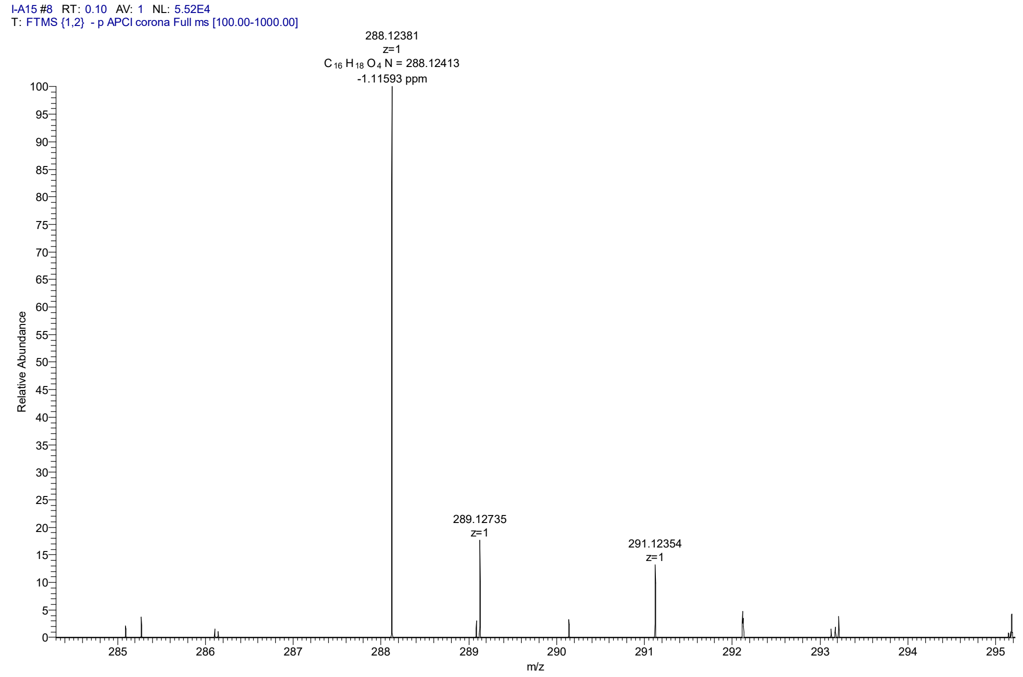

HRMS Spectrum of **5p**

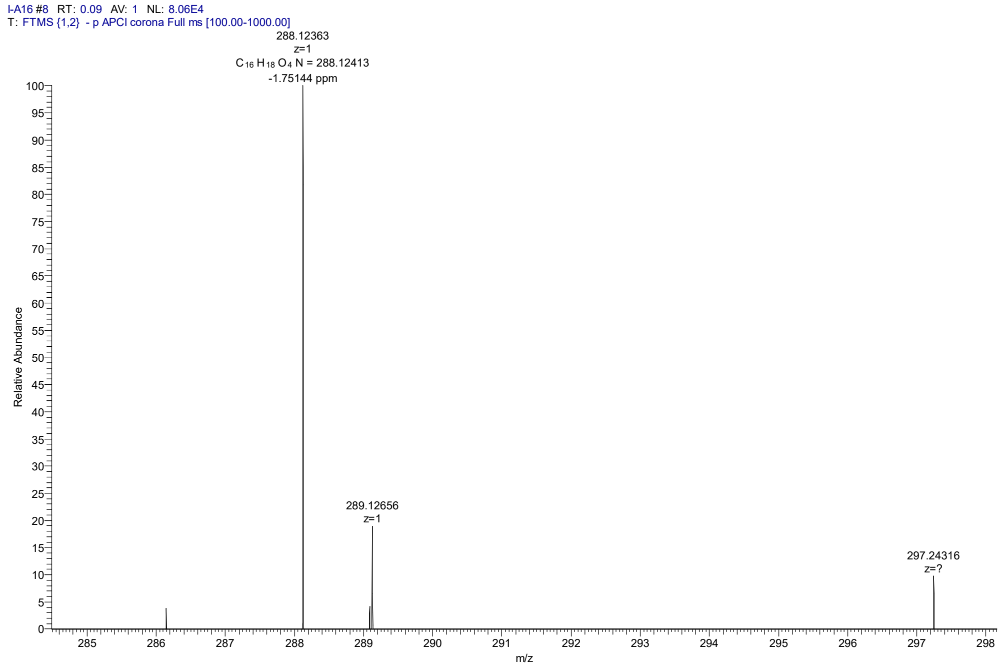

HRMS Spectrum of **5q**

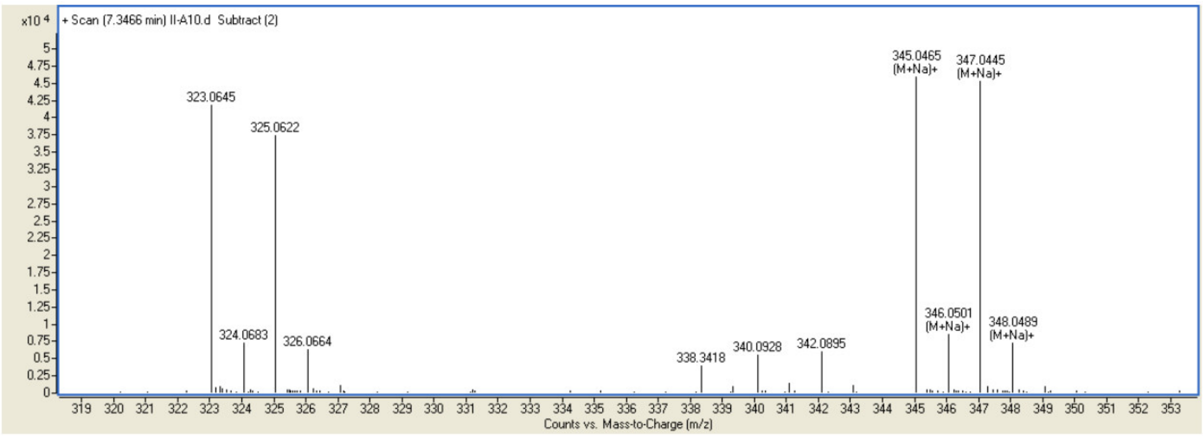

HRMS Spectrum of **5r**

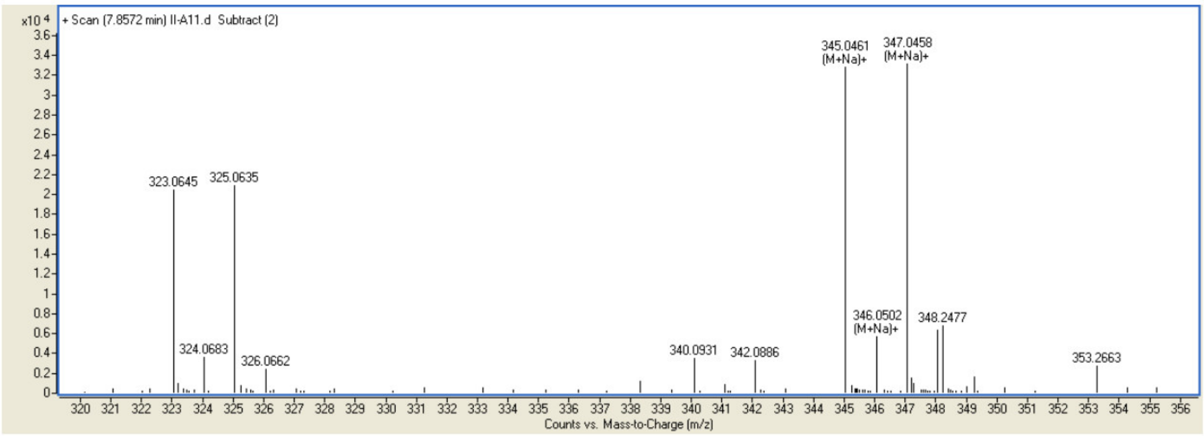

HRMS Spectrum of **5s**

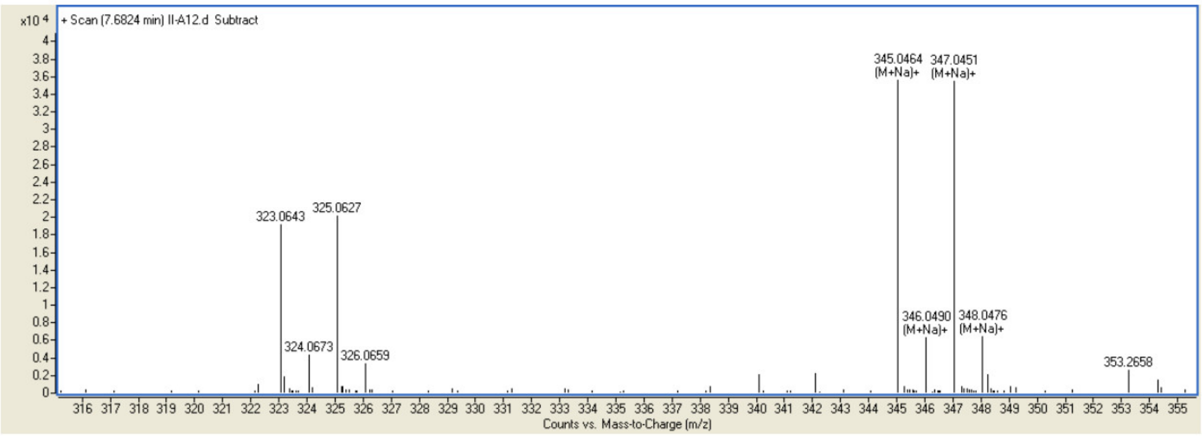

HRMS Spectrum of **5t**

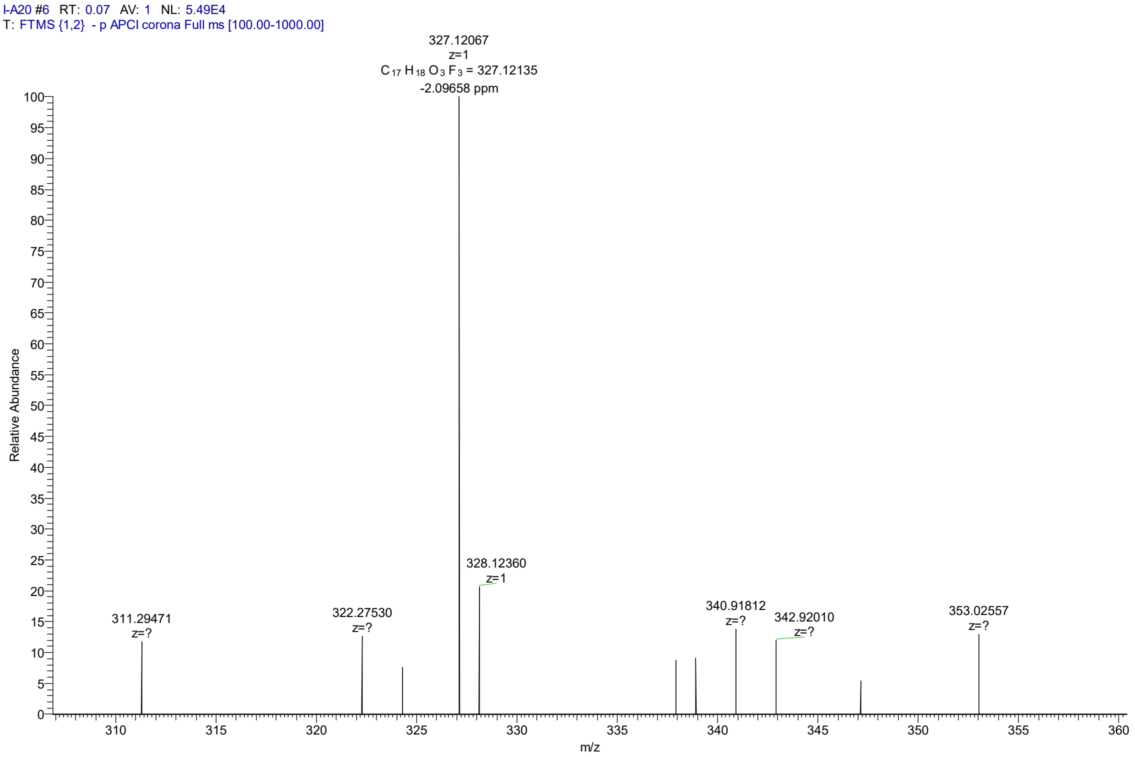

HRMS Spectrum of **5u**

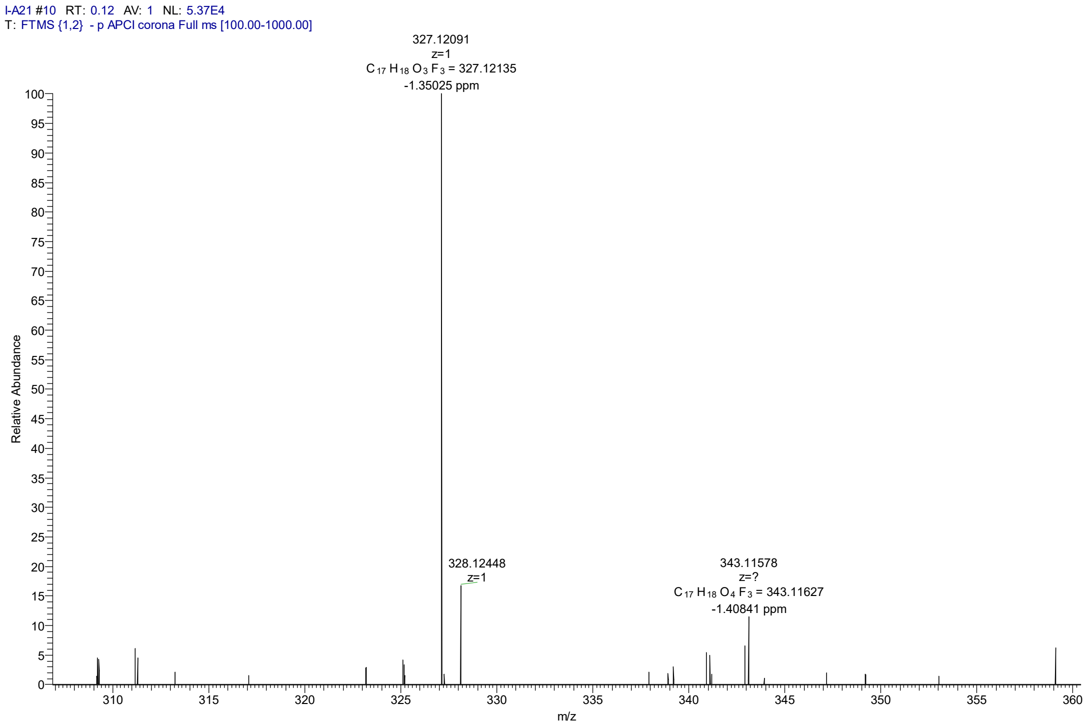

HRMS Spectrum of **5v**

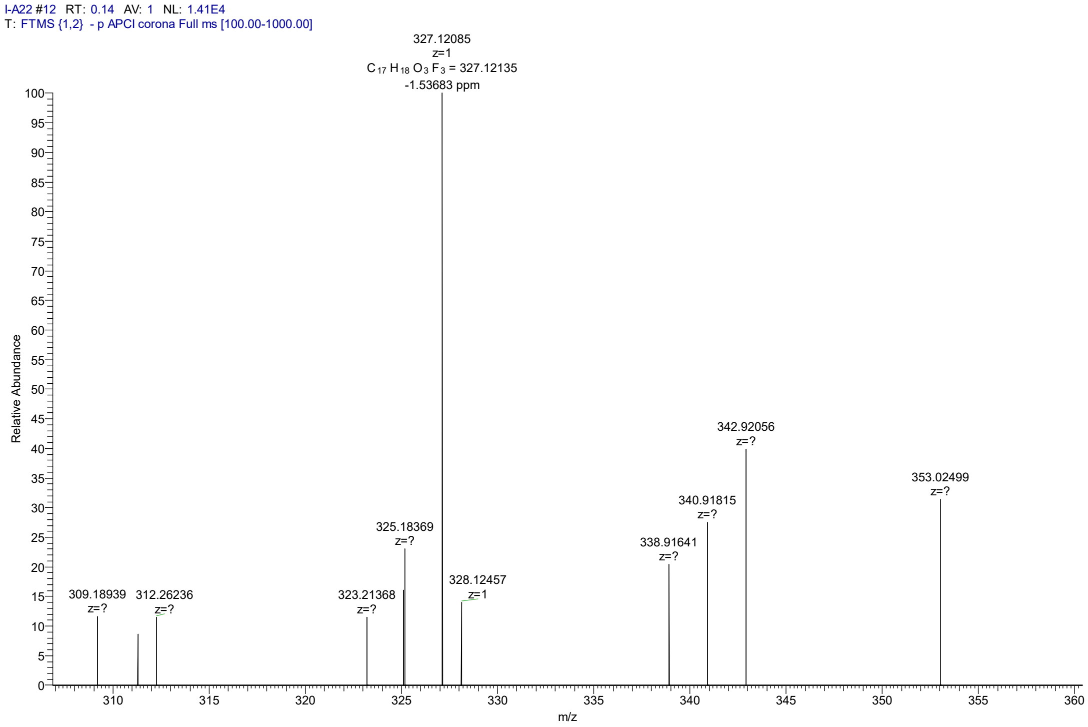

HRMS Spectrum of **5w**

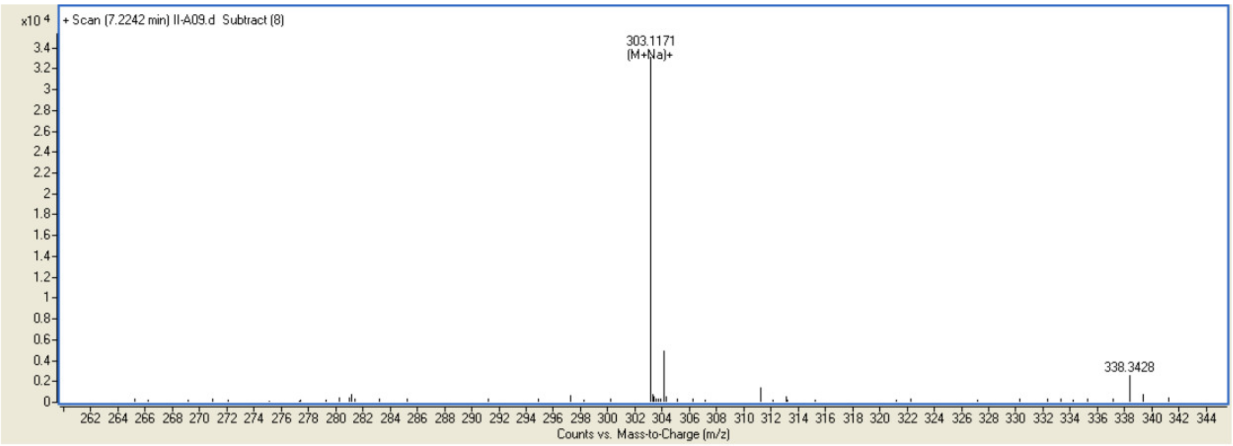

HRMS Spectrum of **5x**

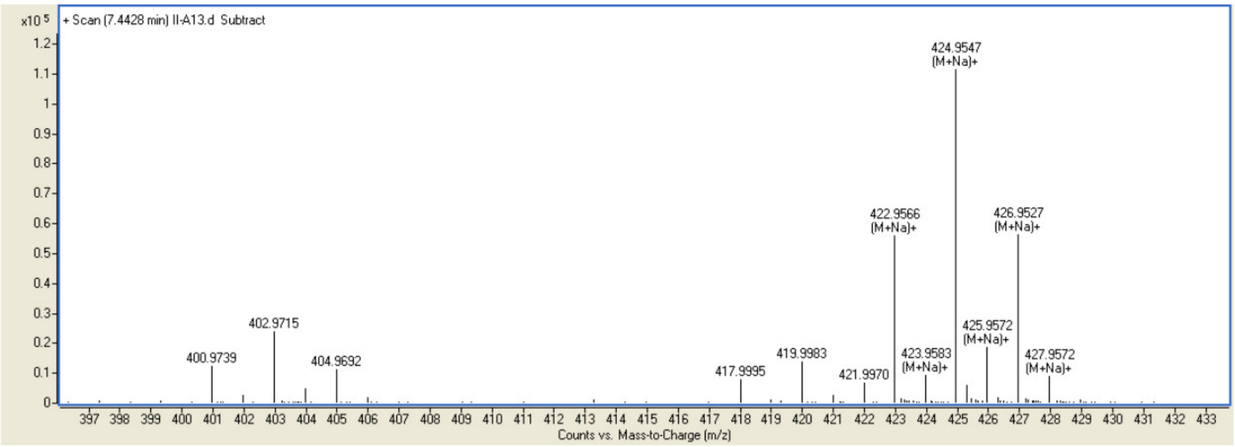

Supplement: Supplementary file 1 [file molecules-27-05949-s001.zip › molecules-1904675-supplementary.pdf]
